# The Encapsulation Strategies for Targeted Delivery of Probiotics in Preventing and Treating Colorectal Cancer: A Review

**DOI:** 10.1002/advs.202500304

**Published:** 2025-04-07

**Authors:** Hao Zhong, Jin Jiang, Muhammad Hussain, Haoxuan Zhang, Ling Chen, Rongfa Guan

**Affiliations:** ^1^ College of Food Science and Technology Zhejiang University of Technology Hangzhou 310014 China; ^2^ Moganshan Institute ZJUT Kangqian Deqing 313200 China; ^3^ Sanya Branch of Hainan Academy of Inspection and Testing San Ya 572011 China

**Keywords:** colorectal cancer, nanoencapsulation, probiotics, protective mechanism, targeted delivery systems

## Abstract

Colorectal cancer (CRC) ranks as the third most prevalent cancer worldwide. It is associated with imbalanced gut microbiota. Probiotics can help restore this balance, potentially reducing the risk of CRC. However, the hostile environment and constant changes in the gastrointestinal tract pose significant challenges to the efficient delivery of probiotics to the colon. Traditional delivery methods are often insufficient due to their low viability and lack of targeting. To address these challenges, researchers are increasingly focusing on innovative encapsulation technologies. One such approach is single‐cell encapsulation, which involves applying nanocoatings to individual probiotic cells. This technique can improve their resistance to the harsh gastrointestinal environment, enhance mucosal adhesion, and facilitate targeted release, thereby increasing the effectiveness of probiotic delivery. This article reviews the latest developments in probiotic encapsulation methods for targeted CRC treatment, emphasizing the potential benefits of emerging single‐cell encapsulation techniques. It also analyzes and compares the advantages and disadvantages of current encapsulation technologies. Furthermore, it elucidates the underlying mechanisms through which probiotics can prevent and treat CRC, evaluates the efficacy and safety of probiotics in CRC treatment and adjuvant therapy, and discusses future directions and potential challenges in the targeted delivery of probiotics for CRC treatment and prevention.

## Graphical abstract

This work summarizes the efficacy and mechanisms of probiotics in CRC treatment, providing a comprehensive overview of various encapsulation methods such as microcapsules, hydrogels, single‐cell nanocoatings, and nanofibers for targeted probiotic delivery to inflammatory sites, with particular emphasis on the potential advantages of emerging single‐cell encapsulation techniques. Additionally, it discusses clinical translational limitations, challenges, and prospects of probiotic encapsulation in CRC therapy.

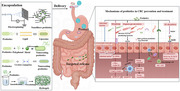

## 1 Introduction

Colorectal cancer (CRC) is characterized by a high incidence and mortality rate. According to the International Agency for Research on Cancer, by 2040, there will be 32 million new cases, resulting in 16 million deaths, representing an increase of 63% and 73·4%, respectively.^[^ ^1^ ^]^ CRC involves both genetic and sporadic, with the majority being of the latter type. Environmental factors such as dietary habits, smoking, obesity, and high levels of alcohol intake significantly contribute to the development of sporadic CRC. In addition, accumulating evidence reported that dysbiosis in the gut microbiota triggered an immune response and increased the risk of CRC.^[^ ^2^ ^]^ The gut microbiota assumes a pivotal role in sustaining the overall health of the host by facilitating immune system equilibrium, energy metabolism, and shaping the intestinal epithelium as well as mucosal homeostasis.^[^ ^3^ ^]^ Hence, the gut microbiota has emerged as a promising therapeutic target for early CRC in recent years.

Research on multi‐cohort samples has identified that pathogenic bacteria, including *Fusobacterium nucleatum*, *Peptostreptococcus anaerobius*, *Parvimonas micra*, and *Peptostreptococcus stomatis*, are significantly enriched in CRC patients, while probiotics such as *Streptococcus salivarius subsp. thermophilus*, *Lactobacillus gallinarum (L. gallinarum)*, *Carnobacterium maltaromaticum*, *Clostridium butyricum* (*C. butyricum*), *and Streptococcus salivarius* were depleted in CRC patients.^[^ ^4^ ^]^ The beneficial modulation of the gut microbiota through the administration of probiotics facilitates the proliferation of health‐boosting bacterial strains, which are currently being harnessed in the advancement of probiotic therapeutic interventions. Studies have shown that treatment with probiotics (e.g., *L. gallinarum*, *Lacticaseibacillus rhamnosus* GG and *Lacticaseibacillus rhamnosus* LS8) enhances commensal probiotics, including *Lactobacillus helveticus* (*L. helveticus*), *Limosilactobacillus reuteri* (*L. reuteri*), *Faecalibaculum*, *Akkermansia muciniphila* (*AKK*), *Roseburia*, while reducing pathogenic species of *Alistipes*, *Allobaculum*, *Dorea*, *Odoribacter*, *Parabacteroides*, and *Ruminococcus*.^[^ ^5^ ^]^ Thus, probiotics signify a pioneering research avenue for the prevention and treatment of CRC.

**Figure 1:**
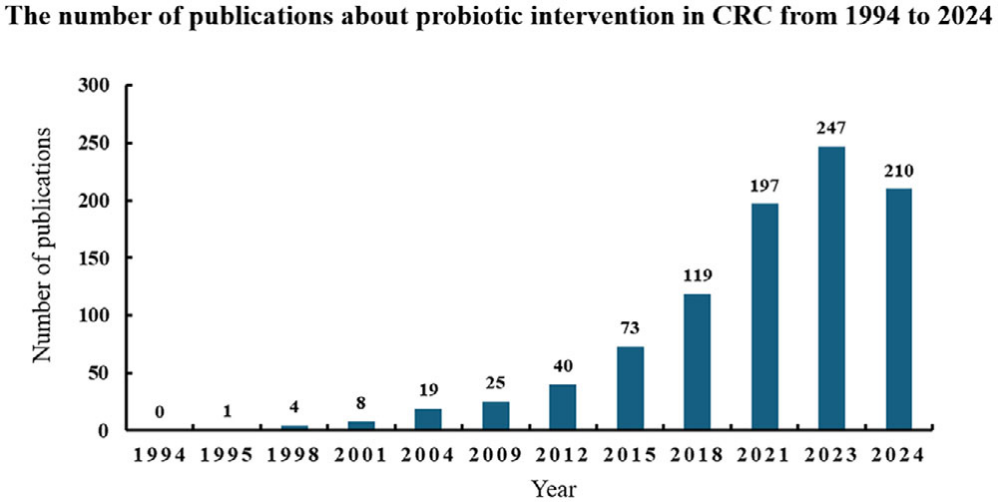
The number of publications about probiotic intervention in CRC from 1994 to 2024 (until December 2024). The data were obtained by searching in Web of Science (https://www.webofscience.com) on the topic of “probiotics” and “colorectal cancer”.

The utilization of probiotics in the treatment of CRC has witnessed notable advancements in recent years.^[^ ^6^ ^]^ (**Figure** **1**) Three cellular pathways have been proposed to elucidate the favorable impacts of probiotics on gut health.^[^ ^9^ ^]^ Initially, probiotics counteract the deleterious effects of pathogenic bacteria by producing bactericidal substances and engaging in competition with pathogens and toxins for adherence to the gut epithelium. Subsequently, they regulate immune responses by enhancing innate immunity and orchestrating the inflammation pathways triggered by pathogens.^[^ ^7^ ^]^ Ultimately, probiotics contribute to the maintenance of intestinal epithelial homeostasis via stimulating intestinal epithelial cell survival, barrier function, and protection.^[^ ^8^ ^]^

Nonetheless, for probiotics to be effective, they must survive the harsh gastrointestinal environment, which includes low gastric pH, enzymatic degradation, and the antimicrobial activity of bile salts. Microencapsulation is an ideal option used to enhance the resistance of probiotics to these unfavorable conditions.^[^ ^9^ ^]^ However, conventional microencapsulation faces inherent disadvantages, such as a lack of size control for microgels, low viability and bioavailability of probiotics, and insufficient targeting of tumor tissues.^[^ ^10^ ^]^ Therefore, recent studies have focused on developing more robust delivery systems. For instance, methodologies for single‐cell encapsulation utilizing liposomes, graphene, polydopamine, and metal‐polyphenol nanoshells have been meticulously developed.^[^ ^11^ ^]^ These encapsulation systems have superior advantages: 1) improved vitality, stability, and bioavailability of probiotics; 2) responsiveness of some materials; 3) a reduction in the size of probiotic carriers, facilitating targeted delivery to the colon through epithelial permeability and retention effects; 4) an augmented mucoadhesive capacity within the colonic environment; 5) preferential adherence and augmented colonization at the disease site. Targeted delivery of probiotics to the disease site and controlled release in response to the pathological microenvironment show great potential in CRC treatment.

This article comprehensively summarizes the effectiveness and safety of probiotics used in CRC treatment and adjuvant therapy. It then systematically reviews the potential mechanisms of probiotics in the prevention and treatment of CRC. This review mainly focuses on describing the current progress in different probiotic encapsulation methods for targeted delivery in CRC treatment. It also analyses the limitations and advantages of current modification strategies for probiotics. It finally anticipates the future development direction and application prospects of probiotics encapsulation systems, aiming to better apply probiotics in targeted CRC therapy in the future. Some recent reviews have been published on similar topics, such as “ Probiotics intervention in CRC: From traditional approaches to novel strategies”^[^ ^12^ ^]^ and “Advances in colon‐targeted nano‐drug delivery systems: challenges and solutions”,^[^ ^13^ ^]^ yet no comprehensive data is available regarding targeted delivery of probiotics for the treatment of CRC. This article is poised to establish a precedent and provide useful guidelines for CRC prevention and treatment.

## 2 The Efficacies of Probiotics in Treatment and Adjuvant Therapy of CRC

### 2.1 The Intervention of Probiotics in Chemotherapy

Presently, the preventive approaches for CRC are predominantly predicated upon lifestyle alterations, screening of high‐risk individuals, the excision of polyps, and the administration of aspirin.^[^ ^14^ ^]^ Clinical strategies for CRC can be broadly divided into three main approaches: surgery resection, chemotherapy, and radiotherapy. Chemotherapy regimens, which comprise 5‐fluorouracil (5‐FU), oxaliplatin (OX), irinotecan (IRI), and capecitabine (CAP), are commonly administered as the initial therapeutic intervention for cancer patients. However, the development of chemo‐resistance in the entirety of CRC patients substantially diminishes the efficacy of anticancer agents, ultimately culminating in therapeutic failure.^[^ ^14^ ^]^ This resistance has the potential to elevate mortality rates by virtue of its deleterious short‐ and long‐term adverse effects, which encompass decrements in body weight, atrophy of skeletal muscle, exhaustion, and psychiatric comorbidities. Additionally, the chemotherapeutic protocols employed for the treatment of gastrointestinal malignancies disrupt gut homeostasis, culminating in pronounced gastrointestinal toxicity that markedly diminishes the overall quality of life for the patient.^[^ ^15^ ^]^ For example, some researchers have observed that the therapeutic regimen of irinotecan (CPT‐11) had the potential to modify the composition of the intestinal microbiota, thereby facilitating the proliferation of β‐glucuronidase‐producing pathogenic bacteria, such as *Escherichia coli* (*E. coli*), *Staphylococcus* and *Clostridium*, which consequently resulted in enterotoxicity.^[^ ^16^ ^]^ Probiotics have a positive effect on alleviating chemotherapy's side effects. Motoori et al. found that the administration of synbiotics, including *Lacticaseibacillus casei* (*L. casei*) strain Shirota, *Bifidobacterium breve* strain Yakult, and galactooligosaccharide, along with enteral nutrition (EN) containing omega‐3 fatty acids during neoadjuvant chemotherapy (NAC), significantly reduced febrile neutropenia and severe diarrhea compared to the antibiotic group.^[^ ^17^ ^]^ Mego et al. also described the incidence of diarrhea and enterocolitis was lower in metastatic CRC patients undergoing irinotecan‐based chemotherapy when they were also given a synbiotic mixture. This mixture contained a variety of *Bifidobacterium* and *Lactobacillus spp*., *Streptococcus salivarius subsp. thermophilus*, maltodextrin, magnesium stearate, ascorbic acid, and inulin.^[^ ^18^ ^]^ An extensive meta‐analysis of 2982 cancer patients suggested that probiotics may be an affordable and safe intervention to reduce infection and diarrhea complications in this population.^[^ ^19^ ^]^ Moreover, probiotic or synbiotic pretreatment has been shown to restore gut function and is correlated with better outcomes, including improved quality of life and long‐term survival.^[^ ^20^ ^]^

Recent studies suggest that probiotics and their specific metabolites could modulate the antitumor efficacy of chemotherapy and immunotherapy by shaping host immunity and balancing the gut microbiota.^[^ ^21^ ^]^ For example, research has discovered that butyrate is capable of directly modulating the anti‐cancer activity of CD8^+^ T cell response and improving the effectiveness of OX through an ID2‐mediated interleukin‐12 (IL‐12) pathway.^[^ ^22^ ^]^ Ren et al. found that *Bifidobacterium animalis subsp. Lactis SF* enhanced the anticancer properties of irinotecan. This was achieved by protecting against intestinal harm through regulating the gut microbiota and decreasing the proportion of pro‐inflammatory bacteria.^[^ ^23^ ^]^ Furthermore, the probiotic combination containing *Bifidobacterium infants*, *Lactobacillus acidophilus* (*L. acidophilus*), *Enterococcus faecalis*, and *Bacillus cereus* effectively reduced chemotherapy‐induced gastrointestinal complications, particularly diarrhea. This combination also increased bacterial diversity in the gut microbiota of CRC patients, and mildly elevated the genus levels of *Bifidobacterium*, *Streptococcus*, and *Blautia*.^[^ ^24^ ^]^ Furthermore, the administration of a blend of probiotics, comprising *Bifidobacterium longum*, *L. reuteri*, and *Lactobacillus johnsonii* led to a marked rise in the presence of macrophages and the levels of IL‐10 in colonic, splenic, and bone marrow samples. This treatment also mitigated oxaliplatin‐induced toxicity in tumor‐bearing mice models and enriched short‐chain fatty acids, which further engaged dendritic cells and macrophages to promote IL‐10 secretion and ameliorate chemotherapy‐induced toxicity.^[^ ^25^ ^]^ These findings underlined the beneficial effects of probiotic interventions as innovative alternatives or complementary strategies in chemoprevention, highlighting their role in enhancing treatment outcomes and patient quality of life.

### 2.2 The Intervention of Probiotics in Radiotherapy

Radiotherapy plays an essential role in cancer treatment, with an estimated 50–60% of cancer patients receiving radiotherapy as part of their treatment.^[^ ^26^ ^]^ Meanwhile, advancements in radiotherapy have significantly lessened its side effects, including damage to surrounding healthy tissues.^[^ ^27^ ^]^ Emerging evidence from preclinical and clinical studies suggests that probiotics can help protect normal tissues during radiotherapy and may even prevent radiation‐induced injuries. For instance, an animal study led by Hua et al.^[^ ^28^ ^]^ demonstrated that supplementation of *L. casei* ATCC334 as a probiotic agent alleviated radiation‐induced intestinal damage. This was achieved by stimulating the proliferation of intestinal stem cells (ISCs), increasing the expression of tight junction proteins, reducing intestinal permeability, safeguarding intestinal barrier integrity, and remodeling gut microbiota structure and metabolic activity. In a subsequent experiment involving animals, male Wistar rats were given a probiotic mixture that included *L. acidophilus*, *L. helveticus*, and *Bifidobacterium spp*. to assess the impact of this mixture on severe intestinal inflammation caused by radiation, with a focus on endotoxemia and bacterial translocation. The findings suggested that probiotics may provide substantial benefits in treating and preventing radiation‐induced intestinal inflammation.^[^ ^29^ ^]^ Moreover, a large‐scale Italian trial, characterized by its double‐blind, placebo‐controlled, and randomized design, enrolled 490 individuals receiving post‐surgical radiation treatment for either colorectal or cervical malignancies. Participants were administered probiotic bacteria comprising various *Lactobacilli*, *Bifidobacteria*, and *Streptococcus*. The findings indicated a marked decrease in the incidence of diarrhea.^[^ ^30^ ^]^ It was concluded that probiotics were increasingly recognized for their potential to avert radiation‐induced toxicity, highlighting their role as a valuable adjunct in radiotherapy treatment protocols.^[^ ^31^ ^]^

### 2.3 Effectiveness of Probiotics in Prevention and Treatment of CRC

Studies have revealed that probiotics can induce apoptosis or inhibit the growth of CRC cells. The role of probiotics in preventing and treating cancer is attributed to multiple pathways, such as the biosynthesis of compounds exhibiting anticarcinogenic properties, modulation of gut microbiota composition, suppression of cellular proliferation and promotion of apoptotic pathways in malignant cells, regulation of mutagenic and carcinogenic factors, immunomodulation, and enhancement of intestinal barrier.^[^ ^32^ ^]^ A summary of these mechanisms for different probiotics is listed in **Table** **1**.

**Table 1:** Probiotics, NGPs, and postbiotics in treatment of CRC.

Items	Effects on CRC	Mechanisms	Refs.
Probiotics			
*Lactobacillus* *gallinarum*	Inhibit tumor formation; Promote apoptosis;	Alter the gut microbiota's composition and induce the secretion of indole‐3‐lactic acid (ILA)	[5a]
*Lactobacillus* *gallinarum*	Attenuat inflammation; Increase anti‐PD‐1 efficacy	*L. gallinarum* and its derived indole‐3‐carboxylic acid (ICA) mitigate the infiltration of regulatory T cells and enhance the cytotoxic function of CD8^+^ T lymphocytes via the regulation of the IDO1/Kyn/AHR metabolic pathway.	[84]
*Limosilactobacillus reuteri*	Inhibit tumor formation	Reuterin induces protein oxidation and inhibits ribosomal biogenesis	[114]
*Lacticaseibacillus paracasei* sh2020	Strengthen intestinal barrier integrity	Upregulate expression of CXCL10 and enhance CD8^+^ T cell recruitment; Modulate gut microbiota;	[78]
*Lacticaseibacillus* *casei* BL23	Attenuate inflammation	Downregulate the IL‐22 cytokine and upregulate caspase‐7, caspase‐9, and Bik	[78]
*Lacticaseibacillus casei*	Attenuate inflammation	Increase in interferon gamma (IFN‐γ), Granzyme B, and chemokine; Enhance CD8^+^ T cell infiltration.	[81]
*Lacticaseibacillus casei* ATCC 393	Induce apoptosis	Induce ROS generation	[54]
*Lacticaseibacillus casei* JY300‐8	Activate apoptosis; Regulate gut microbiota	Activate apoptosis of colon cells and regulate the gut microbiota.	[36]
*Lacticaseibacillus rhamnosus* Probio‐M9	Increase anti‐PD‐1 efficacy Attenuate inflammation	Promote beneficial microbes and metabolites; Promote CD8^+^ cytotoxic T lymphocytes (CTLs) and suppress Tregs.	[34]
*Lacticaseibacillus rhamnosus* GG	Inhibit tumor proliferation	Upregulate CEA gene expression and its protein (CEA).	[41]
*Lacticaseibacillus rhamnosus* LS8	Inhibit tumor formation; Strengthen intestinal barrier integrity	Prevent goblet cell loss and promote the expression of ZO‐1, occludin, and claudin‐1; Inhibit the overexpression of TLR4/NF‐κB.	[5c]
*Lactiplantibacillus plantarum* L168	Attenuate inflammation	ILA accelerates IL‐12 production in DCs to prime anti‐tumor immunity of CD8^+^ T cells; Inhibit expression of Saa3 in CD8^+^ T cells.	[8a]
*Lactiplantibacillus plantarum* YYC‐3	Inhibit tumor formation; Attenuate inflammation	Suppress activation of the NF‐κB and Wnt signaling pathways	[65c]
*Lactiplantibacillus plantarum*‐12	Regulate the intestinal microbiota; Inhibit NF‐κB signaling way; Induce apoptosis	Downregulate TNF‐α, IL‐8 and IL‐1β and upregulate IL‐10; Downregulate PCNA and upregulate Bax	[77]
*Lactobacillus acidophilus*	Induce apoptosis	Induce apoptosis; Evaluate the mRNA expression levels of apoptosis‐related genes (survivin and smac)	[97]
*Lactobacillus acidophilus* CGMCC 878	Regulate gut microbiota	Decrease the intestinal pathogenic bacteria and increase beneficial bacteria	[120]
*Bifidobacterium animalis subsp. lactis SF*	Induce apoptosis	Reduce TGF‐β and inhibit the PI3K/ AKT pathway activation	[23]
*Bifidobacterium longum subsp. longum*	Induce apoptosis; Inhibit tumor proliferation	Induce the tumor suppressor miRNAs (miR‐145 and miR‐15a) expression;	[100]
*Streptococcus salivarius subsp. thermophilus*	Inhibit cell proliferation	β‐Galactosidase induces cell cycle arrest, and promotes apoptosis; Downregulate the Hippo pathway kinases	[119]
NGPs			
*Faecalibacterium prausnitzii*	Regulate gut microbiota	Increase gut probiotics and decrease potential gut pathogens	[88]
*Enterococcus*	Augmented immunotherapy efficacy	NlpC/p60 peptidoglycan hydrolase SagA activates macrophages; Generate conventional type 1 dendritic cells and prime CD8^+^ T cells	[83]
*Leuconostoc mesenteroides*	Promote apoptosis	Upregulate MAPK1, Bax, and caspase 3, and downregulate AKT, NF‐κB, Bcl‐x_L_ expressions and some key oncomicroRNAs such as miRNA‐21 and miRNA‐200b	[91]
*Ruminococcus gnavus* and *Blautia producta*	Inhibit cell proliferation	Degrade lyso‐glycerophospholipids that inhibit CD8^+^ T cell activity	[82]
*Companilactobacillus crustorum* MN047	Inhibit cell proliferation	Suppress the TLR4/NF‐κB pathway; Increase SCFAs and reduce LPS levels.	[65b]
*Clostridium butyricum*	Increased anti‐PD‐1 efficacy	Degradate MYC; Mitigate MYC‐mediated 5‐FU resistance	[8b]
*Clostridium butyricum* ATCC 19398	Induce apoptosis; Modulate the gut microbiota	Suppress the Wnt/β‐catenin signaling pathway; Increase the SCFA quantities and activate G‐protein coupled receptors (GPRs), such as GPR43 and GPR109A.	[65b]
*Roseburia intestinalis*	Attenuate inflammation, Inhibit cell proliferation	Butyrate activates CD8^+^ T cells by directly binding to TLR5, thereby triggering NF‐κB signaling	[39]
*Akkermansia muciniphila* Postbiotics	Attenuated inflammation	Activate toll like receptor 2 (TLR2) signaling pathway; Protect gut barrier function	[86]
Lysates of *Lactobacillus acidophilus*	Inhibited tumor formation	Inhibit the M2 polarization and the IL‐10 expressed levels of LPS‐activated Raw264.7 macrophages.	[85]
*Lactobacillus delbrueckii subsp. bulgaricus* OLL1073R‐1	Increased anti‐PD‐1 efficacy, Attenuated inflammation	Increase CCR6^+^ CD8^+^T cells infiltration and produce IFN‐γ	[112]
*Lactobacillus pantheris* TCP102 EPS	Inhibited tumor proliferation	Induce the production of nitric oxide (NO), TNF‐α, and IL‐6	[113]
*Lactobacillus*‐CLA	Inhibit cell proliferation, Attenuated inflammation	CLA lowerCyclindependent kinase 1 (CDK1) /2/6, PLK1, and SKP2; Downregulate pro‐inflammatory cytokine and upregulate anti‐inflammatory cytokine gene expressions.	[74]
Extracellular vesicles of *Lactiplantibacillus plantarum (LpEVs)*		LpEVs enhance 5‐FU sensitivity in CRC/5FUR cells by reducing PDK2 expression via p53‐p21 metabolic signaling, overcoming drug resistance.	[216]

Various *Lacticaseibacillus* strains have shown robust anti‐tumor properties in CRC, as illustrated by pre‐clinical studies in animal models.^[^ ^33^ ^]^ Notably, *Lacticaseibacillus rhamnosus* (*L. rhamnosus*) Probio‐M9 has exhibited potent inhibitory effects on CRC, particularly when combined with anti‐programmed cell death receptor‐1 (PD‐1) treatment.^[^ ^12^, ^34^ ^]^ This synergistic approach highlights the potential of combining probiotics with immunotherapies. Additionally, *Lactiplantibacillus plantarum* (*L. plantarum)* L168 and its derived metabolite, indole‐3‐lactic acid, have effectively reduced tumor number by 83.3% in mice with colitis‐associated cancer.^[^ ^8a^ ^]^ The anti‐cancer properties of *L. plantarum* S2T10D containing butyrate are further evidenced by its supernatant's ability to suppress the proliferation of HT‐29 cells, halt the cell cycle progression at the G2/M checkpoint, and decrease the levels of cyclin D1 and cyclin B1 expression.^[^ ^35^ ^]^ *L. casei* JY300‐8 has also been demonstrated to significantly reduce the proliferation of Caco‐2 cells to a maximum of 65.27%, as well as that of HT‐29 and HCT‐116 cells, exceeding an 80% reduction.^[^ ^36^ ^]^ Similarly, treatment with *L. casei* ATCC393 for 24 h at a concentration of 10^9^ CFU·mL^−1^ dramatically decreased the viability of CT26 and HT‐29 cells by 52% and 78%, respectively.^[^ ^37^ ^]^

Other microorganisms, including *Lactococcus*, *Streptococcus*, *Enterococcus*, *Bacillus*, and *Roseburia intestinalis* (*R. intestinalis*) are also commonly used as probiotics in CRC.^[^ ^38^ ^]^ For example, *R. intestinalis* administration has been shown to reduce colon tumor incidence by about 60% in Apc^Min/+^ mice.^[^ ^39^ ^]^ These probiotics are selected for their ability to modulate the gut microbiota, enhance immune responses, and directly impact cancer cell biology, offering a multifaceted approach to CRC management. The integration of probiotics into cancer treatment strategies represents a promising avenue for future research and clinical applications.

Probiotics secrete bioactive components such as bacteriocins and polysaccharides, which can exert beneficial effects via direct and/or indirect interaction with cancer cells.^[^ ^40^ ^]^ Researcher have demonstrated that extracellular vehicles (EVs) derived from *L. rhamnosus* GG (LGG) can suppress the proliferation of HT29 and SW480 cells, upregulate the expression of the CEA gene, and increase the production of its protein CEA.^[^ ^41^ ^]^ In another study, *L. paracasei*‐derived EVs significantly reduced the viability of HCT116 cells by 60% to 80% at 200 µg·mL^−1^.^[^ ^42^ ^]^ A therapeutic protein p8 derived from *L. rhamnosus* exhibited potent inhibitory effects on tumor growth, leading to a significant reduction in tumor volume, with reductions as high as 59% relative to the control group.^[^ ^43^ ^]^ Additionally, *Bifidobacterium animalis subsp. lactis SF* was found to produce substantial quantities of exopolysaccharides (EPS). Upon administration to HCT‐8 cells at a concentration of 1600 µg mL^−1^ for 48 h, its inhibition efficacy reached a peak of 41.2%.^[^ ^23^ ^]^

### 2.4 The Safety of Probiotics: Less Drug Resistance and Chronic Toxicity

Probiotics are commonly delineated as “live bacteria that confer health advantages to the host when consumed in sufficient quantities”.^[^ ^44^ ^]^ Traditionally, a wide variety of fermented products like yogurt, kefir, kimchi, sauerkraut, tempeh, miso, and kombucha have been integral to diets, acting as traditional probiotic sources.^[^ ^45^ ^]^ Research into probiotic usage among oncology patients receiving chemotherapy or chemoradiotherapy revealed that 28.5% of them incorporated probiotics into their regimen. Of this group, only 8.5% reported adverse effects such as diarrhea, vomiting, allergy, infection, constipation, and flatulence.^[^ ^46^ ^]^

It is generally believed that probiotics pose minimal risks to patients with typical health profiles. A comprehensive review conducted in 2011 found no substantial elevation in the likelihood of negative outcomes, even severe ones, associated with short‐term probiotic supplementation.^[^ ^47^ ^]^ For example, research indicated that using probiotics or synbiotics around the time of surgery could lessen gastrointestinal issues, such as infections and diarrhea, among patients with CRC. Clinical trials have further supported the safety of probiotics, as cases of sepsis, bacteremia, and infections attributed to probiotics are rare.^[^ ^48^ ^]^ Nonetheless, there are infrequent but potentially severe safety concerns associated with probiotics. For instance, in October 2023, the Food and Drug Administration (FDA) warned against probiotic use in premature infants due to reports of sepsis that resulted in fatalities. This warning is likely due to the immature state of the gastrointestinal tract and immune system in preterm babies, making them more vulnerable to the adverse impacts of probiotics.^[^ ^49^ ^]^ Generally, prebiotics and probiotics seem to be safe for the majority, yet with the scarcity of safety data, additional research is essential to confirm their safety over extended periods, alleviate worries about prolonged use, and identify their suitable applications in various health scenarios.

### 2.5 Probiotic Nanoparticles act as Antibacterial and Anticancer Agents

The application of lactic acid bacteria strains in synthesizing biological nanoparticles has provided a method for developing novel nano‐drug formulations in cancer therapy. Advancements in research have shown that biosynthesized silver nanoparticles (AgNPs) hold promise as both antibacterial and anticancer agents.^[^ ^50^ ^]^ For example, Leila and her colleagues used the probiotic Lacticaseibacillus casei subsp. casei for the synthesis of AgNPs, which were found to inhibit the proliferation of HT‐29, promote apoptosis, and increase nitric oxide (NO) secretion.^[^ ^51^ ^]^ Mousavi et al. synthesized silver nanoparticles combined with L. rhamnosus GG (Ag‐LNPs)^[^ ^52^ ^]^ and discovered that the survival rate of HT‐29 CRC cells was significantly diminished as the Ag‐LNPs concentration escalated. It is suggested that these Ag‐LNPs, synthesized through biological processes, could trigger the production of ROS in the HT‐29 cell line, ultimately resulting in cellular demise.^[^ ^53^ ^]^ Spyridopoulou and colleagues employed the probiotic strain L. casei ATCC 393 to synthesize biogenic Selenium nanoparticles measuring 360 nm in size. Their findings indicated that selenium nanoparticles derived from L. casei, as well as L. casei enriched with selenium nanoparticles, displayed targeted anticancer effects in vitro. These effects encompassed the induction of programmed cell death and an increase in the production of reactive oxygen species (ROS) within malignant cells.^[^ ^54^ ^]^ Bio‐nanomaterials, such as nanoparticles, show immense potential in fighting against multiple drug‐resistant bacterial pathogens, presenting a key tool in tackling the major global issue of drug resistance.^[^ ^55^ ^]^ These nanomaterials can target bacteria through a variety of mechanisms, such as destroying bacterial cell membranes, binding to intracellular components, producing ROS, and more. In addition, nanomaterials can function as drug carriers, improve bioavailability and target drugs, and mitigate the development of bacterial resistance. Studies have shown that with proper surface functionalization, nanomaterials can improve their selectivity against bacteria while minimizing their toxicity to mammalian cells. For example, AgNPs, which are combined with antibiotics, can improve their killing effect on bacteria, while reducing bacterial resistance to antibiotics.^[^ ^56^ ^]^

### 2.6 Engineered Probiotics Improve Therapy Efficacy

In recent years, numerous studies have demonstrated that engineered probiotics are effective in various practical applications. Tang and colleagues engineered a novel probiotic, Ep‐AH, by integrating azurin and hlpA genes into *Escherichia coli Nissle* 1917 (*EcN*). In azoxymethane (AOM)/dextran sodium sulfate salt (DSS)‐treated mice, Ep‐AH exhibited potent anticancer properties, significantly reversing weight loss (*p* < 0.001), fecal occult blood (*p* < 0.01), and colon shortening (*p* < 0.001), while reducing tumorigenesis by 36% (*p* < 0.001) compared to the model group.^[^ ^57^ ^]^

For efficient delivery of engineered *EcN*, some studies have combined it with coating materials. *ECN*‐pE, engineered to overexpress catalase and superoxide dismutase, was coated with chitosan (CS) and sodium alginate (SA) via layer‐by‐layer assembly to enhance gastrointestinal bioavailability. Remarkably, *ECN*‐pE(C/A)_2_ mitigated the severity of DSS‐induced colitis by substantially reducing weight loss, preventing colon shortening, and restoring intestinal barrier integrity.^[^ ^58^ ^]^ In another study, Gu et al. developed a novel probiotic formulation (GM‐*EcN*) by encapsulating *EcN* within intracellularly gelated macrophages (GM). The hydrogel protected *EcN* from gastric digestion, while GM acted as a macrophage‐like carrier that sequestered and neutralized inflammatory cytokines through receptor‐ligand interactions and inflammation‐related membrane proteins. In vivo, GM‐*EcN* effectively mitigated inflammatory bowel disease (IBD) symptoms and promoted the restoration of gut microbiota.^[^ ^59^ ^]^

The click reaction represents an alternative methodology for establishing covalent bonds between nanoparticles and live bacterial cells, exhibiting high specificity, selectivity, and orthogonality. For example, Cao et al. developed self‐adaptive antitumor probiotics by coupling a pH‐sensitive peroxidase‐like nanozyme regulator B‐FeAu with clinically relevant probiotics via click chemistry between phenylboronic acid groups on the nanozyme and bacterial polysaccharides. Mice treated with *Bifidobacterium longum* 9999@B‐FeAu (BL999@B‐FeAu) significantly suppressed tumor growth, reduced tumor nodules, and decreased tumor sizes compared to BL999+B‐FeAu combination therapy or monotherapy with BL999 or B‐FeAu alone.^[^ ^60^ ^]^ These findings highlight the potential of engineered probiotics to treat colitis through synthetic biology, coating, and other methods to develop more effective probiotics and improve targeting.

## 3 Mechanisms of Probiotics in the Prevention and Treatment of CRC

### 3.1 The Role of Probiotics Themselves in CRC

#### 3.1.1 Immune Modulation

The immune response plays a critical role in pathogen defense and suppression of malignant proliferation. Macrophages trigger inflammation by phagocytosing foreign substances and secreting inflammatory molecules upon detection via specific receptors like the Toll‐like receptor 4 (TLR‐4).^[^ ^61^ ^]^ However, prolonged inflammation and overproduction of pro‐inflammatory cells can lead to immune system imbalances, fostering an inflammatory environment that supports the survival and spread of CRC cells. The TLR4/MyD88/NF‐κB signaling pathway in intestinal cells contributes to inflammation‐associated CRC.^[^ ^62^ ^]^ Lipopolysaccharide (LPS) activates TLR4,^[^ ^63^ ^]^ which triggers a cascade involving IκB and p65 phosphorylation and subsequent IκB‐α degradation, allowing nuclear factor kappa (NF‐κB) to translocate to the nucleus. This process initiates the transcription of genes including inducible nitric oxide synthase (iNOS), cyclooxygenase (COX)‐2, and inflammatory cytokines such as interleukin‐1β (IL‐1β), interleukin‐6 (IL‐6), and tumor necrosis factor‐α (TNF‐α).^[^ ^64^ ^]^

**Figure 2:**
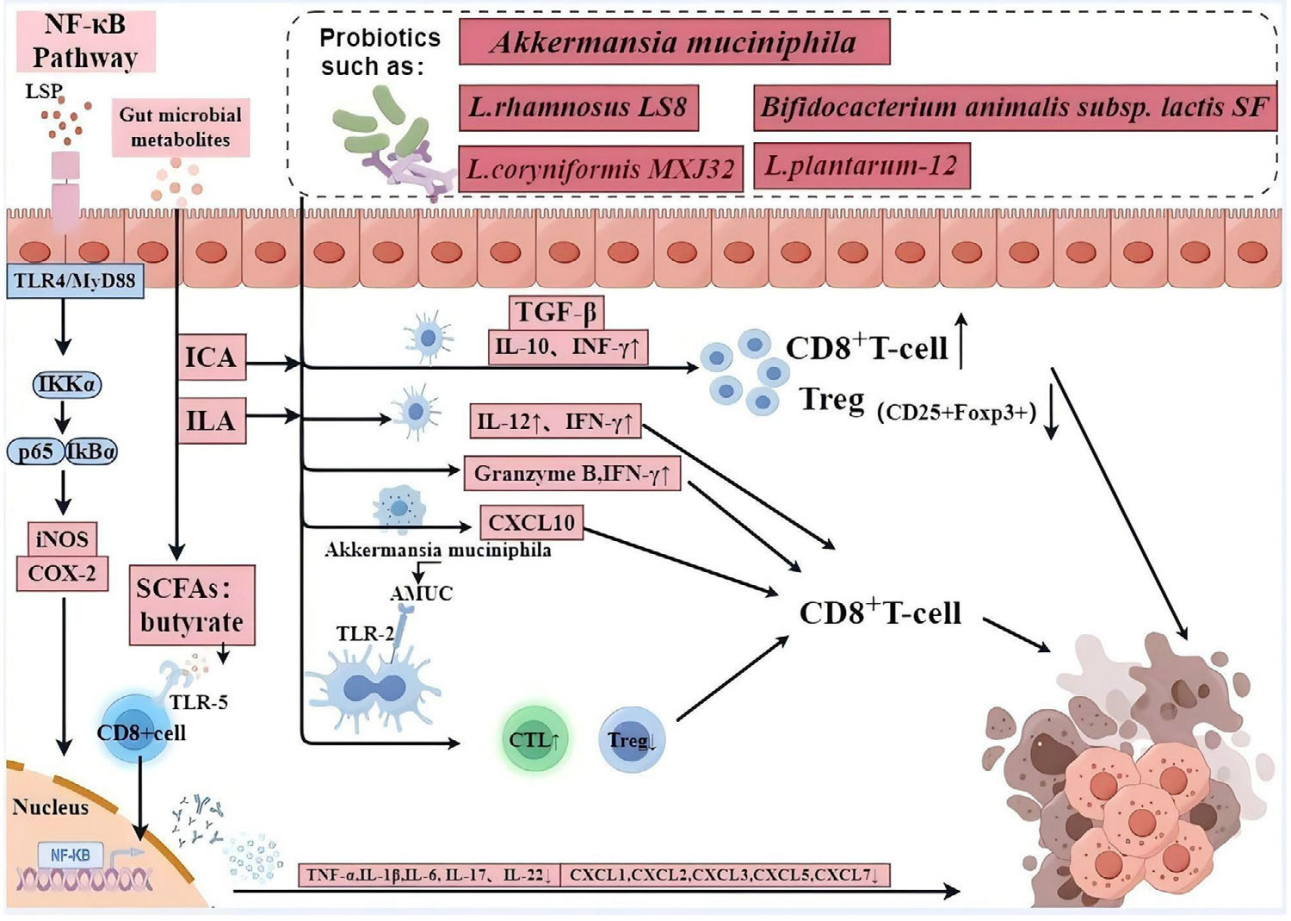
Immune mechanism of probiotics and its metabolite in CRC.

Nevertheless, probiotics have been shown to possess a significant capacity to reduce inflammation and modulate the immune system by maintaining the equilibrium between pro‐ and anti‐inflammatory factors (**Figure** **2**). Data from several studies have revealed that probiotics supplementation, such as with *L. rhamnosus* LS8, *Loigolactobacillus coryniformis* MXJ32 (*L. coryniformis*), *Companilactobacillus crustorum* (*C. crustorum*) MN047, *L. plantarum* YYC‐3, *Limosilactobacillus fermentum* (*L. fermentum*) CQZS40, *C. butyricum* ATCC 19 398, and *Bifidobacterium animalis subsp. lactis SF*, inhibited the increase of serum LPS, attenuated the overactivation of TLR4/MyD88/NF‐κB pathway, and further inhibited the secretion of pro‐inflammatory cytokines (i.e., IL‐6, TNF‐α, interferon (IFN)‐γ, IL‐22 and IL‐1β), and chemokines (CXCL1, CXCL2, CXCL3, CXCL5, and CXCL7).^[^ ^5^, ^7^, ^23^, ^65^ ^]^ Not only that, multi‐strain probiotics mixture also significantly inhibits tumorigenesis and inflammatory response. For example, a mixture of *L. plantarum* KX041, *L. rhamnosus* LS8, *L. coryniformis* MXJ32, and *C. crustorum* MN047 could decrease serum LPS and pro‐inflammatory cytokines.^[^ ^66^ ^]^ Probiotics like *L. acidophilus*, *L. rhamnosus*, and *Bifidobacterium bifidum* reduced inflammatory pathway activity by decreasing phosphorylated IKK and TNF‐α expression while increasing IL‐10 levels.^[^ ^67^ ^]^

Cytokines such as IL‐6 and TNF‐α are key in early tumorigenesis.^[^ ^68^ ^]^ Their effects include disrupting the intestinal mucosal barrier, leading to high intestinal permeability.^[^ ^69^ ^]^ Later, it can aggravate mucosal inflammation and induce an immune response. It is believed to be the key to initiating intestinal diseases, including cancers, triggered by infection and immune factors.^[^ ^70^ ^]^ COX‐2, activated by pro‐inflammatory cytokines and ROS,^[^ ^71^ ^]^ is closely related to NF‐kB and significantly upregulated in colon carcinogenesis.^[^ ^72^ ^]^ Conjugated linoleic acids (CLAs) play crucial roles in probiotics' anti‐inflammatory and anti‐carcinogenic properties.^[^ ^73^ ^]^ Dietary *Lactobacillus* and CLA treatment suppressed the activity of NF‐κB and modulated the expression of cancer progression‐related elements COX‐2 and prostaglandin E2 (PGE2), reducing pro‐inflammatory cytokine levels and Th17‐related inflammatory cytokine genes in CRC cells.^[^ ^74^ ^]^ These effects align with the observed suppression of intestinal mucosa inflammation, infiltration of inflammatory cells, and reduction in pro‐inflammation factors.

Interleukin‐12 (IL‐12) stimulates the expansion of natural killer (NK) and T cells, fostering the secretion of cytotoxic cytokines such as IFN‐γ, crucial for Th1 cell differentiation and anti‐cancer immunity.^[^ ^75^ ^]^ According to previous reviews, the synergistic action of IL‐12, IFN‐γ, CXCL9, and CXCL10 is closely linked to the infiltration of CD8^+^ T cells into CRC and is accompanied by enhanced anti‐tumor activity.^[^ ^76^ ^]^ These findings are confirmed in the following literature. Ma et al. reported that treatment with *L. plantarum* L168 and its metabolite, indole‐3‐lactic acid (ILA), could enhance CD8^+^ T cell‐mediated anti‐tumor cytotoxicity, with ILA potentially hastening IL‐12 synthesis in dendritic cells (DCs), thereby activating the anti‐tumor potential of CD8^+^ T cells.^[^ ^77^ ^]^ Similarly, Zhang and colleagues revealed that *L. paracasei* sh2020 induced an increase in CXCL10 levels within tumors, recruiting more CD8^+^ T cells.^[^ ^78^ ^]^

Previous studies have indicated that the secretion of CXCL10 and CCL5 in tumors is correlated with the aggregation of granzyme‐expressing CD8^+^ lymphocytes and IFN‐γ‐secreting CD4^+^ T cells in CRC, particularly in early TNM stages.^[^ ^79^ ^]^ The accumulation of these T cells, cytotoxic CD8^+^ T cells, and the presence of IFN‐γ and granzyme B, aims to eradicate cancer cells.^[^ ^80^ ^]^ This has been confirmed in studies where *R. intestinalis* and *L. casei* enhanced anti‐cancer effects by activating cytotoxic CD8^+^ T cells and increasing granzyme B, IFN‐γ, and TNF‐α levels.^[^ ^39^, ^81^ ^]^ Certain bacteria from the *Lachnospiraceae* family, Rg and Bp, can also break down lyso‐glycerophospholipids, which in turn raises the levels of granzyme B and IFN‐γ, thereby enhancing CD8^+^ T cell function in combating tumors.^[^ ^82^ ^]^

Furthermore, studies have highlighted the significant role of probiotic supplementation in enhancing the effectiveness of immunotherapies targeting CTL‐associated antigen‐4 (CTLA4), PD‐1, and PD‐L1.^[^ ^83^ ^]^ For instance, *L. gallinarum* synergized with anti‐PD1 therapy by diminishing the presence of Foxp3^+^ CD25^+^ regulatory T cell (Treg) within tumors and bolstering the functionality of CD8^+^ T cells.^[^ ^84^ ^]^ Another study reported that *Clostridium butyricum* (C.B) potentiated the responsiveness to anti‐PD1 therapeutic intervention by enhancing proteasome‐dependent ubiquitination pathways, thereby elevating the expression levels of CD4, CD8, and Granzyme B, which included the degradation of MYC protein and augmented the effectiveness of 5‐FU chemotherapy.^[^ ^8b^ ^]^ Members of the bacterial genus *Enterococcus* have demonstrated the ability to enhance the efficacy of checkpoint inhibitor therapies. Active strains of *Enterococcus* produced and secreted homologs of the NlpC/p60 peptidoglycan hydrolase, SagA, which in turn produced immunostimulatory muropeptides and thereby bolstered the antitumor effects of anti‐PD‐L1 treatment.^[^ ^83^ ^]^ Zhuo and colleagues reported that lysates of *L. acidophilus* could intensify the tumor‐inhibiting capabilities of CTLA‐4 monoclonal antibodies by elevating the counts of CD8^+^T cell and memory T cells (CD44^+^CD8^+^CD62L^+^), while also decreasing Treg (CD4^+^CD25^+^Foxp3^+^).^[^ ^85^ ^]^ Shi et al. reported that concurrent administration of IL‐2 and AKK can lead to superior tumor management, characterized by an increase in tumor‐specific cytotoxic T lymphocytes (CTLs) and a reduction in immunosuppressive Tregs within the tumor microenvironment. The tumor‐inhibiting immune response triggered by AKK is believed to be due to its outer membrane protein Amuc, which activates Toll‐like receptor‐2 (TLR2) signaling pathways, leading to effective tumor regression.^[^ ^86^ ^]^ It was also found that *L. rhamnosus* GG stimulated the activity of CD8^+^ T cells activity through TLR2 receptors present on dendritic cells, subsequently increasing dendritic cell counts and enhancing the response of CD8^+^ T cells.^[^ ^87^ ^]^ Besides, the oral administration of *Faecalibacterium prausnitzii* has been shown to increase the infiltration of CD3^+^ and CD8^+^ T‐cell, as well as the levels of IFN‐γ and TNF‐α in these cells, thus potentiating the effects of dual checkpoint blockades against CTLA4 and PD‐1.^[^ ^88^ ^]^

#### 3.1.2 Induction of Apoptosis

**Figure 3:**
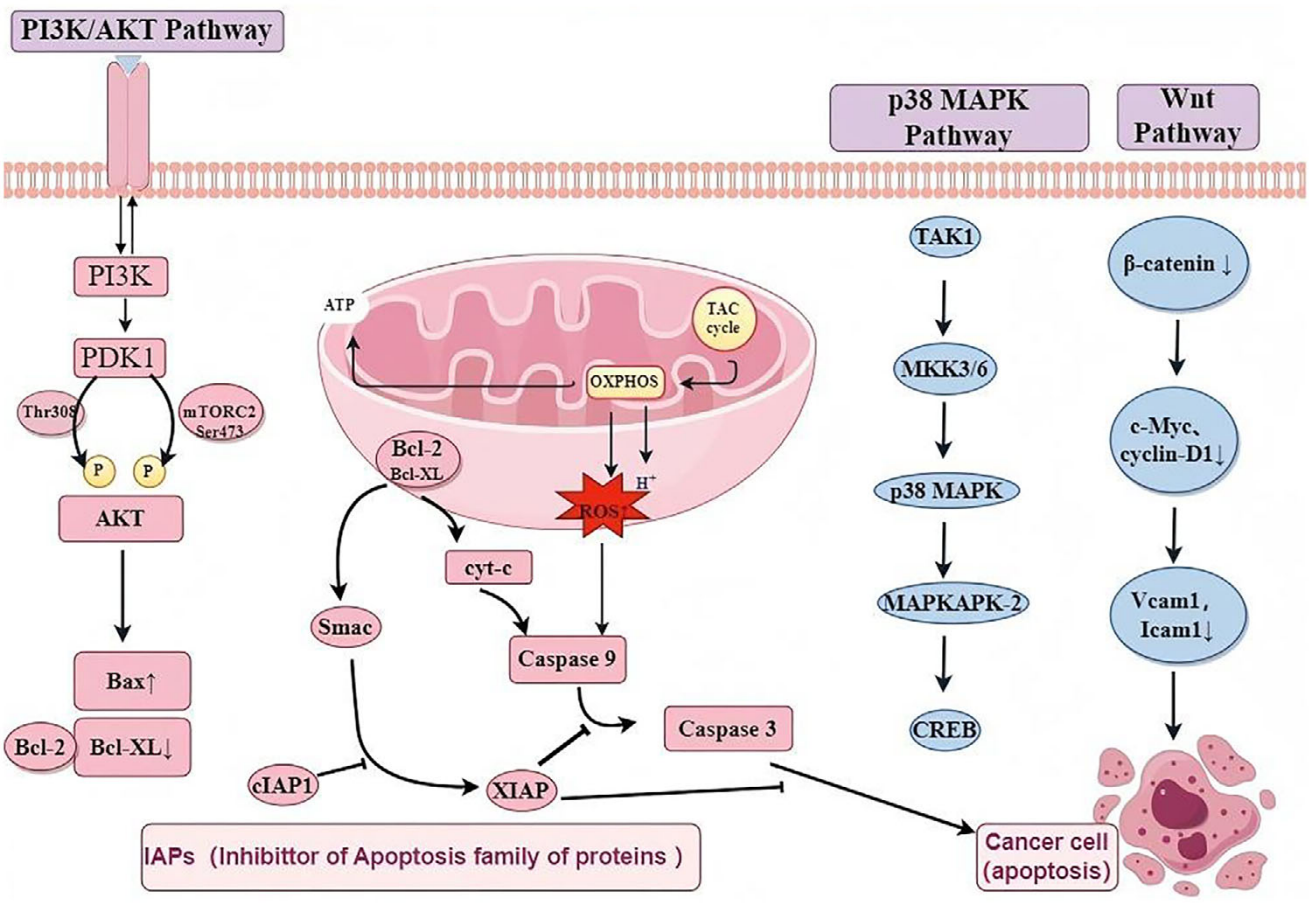
Apoptosis‐inducing mechanism of probiotics in CRC.

Unrestrained cell division and the disruption of programmed cell death mechanisms are key features of cancer. As shown in **Figure** **3**, probiotics can inhibit cancer cell growth and stimulate apoptosis by upregulating pro‐apoptotic and downregulating anti‐apoptotic proteins. In a case study of *L. plantarum* YYC‐3, it strongly reduced the expression level of β‐catenin protein, a key step in the Wnt pathway, leading to decreased expression of genes that promote apoptosis and inhibit proliferation (c‐Myc, cyclin‐D1, Vcam1, and Icam1), thereby preventing CRC in mice.^[^ ^65c^ ^]^ Similar effects have been reported for *Bifidobacterium animalis subsp. lactis SF* ^[^ ^23^ ^]^ and *C. butyricum*.^[^ ^89^ ^]^

B‐cell lymphoma‐2 (Bcl‐2) is a major apoptosis inhibitor, while B‐cell lymphoma‐extra large (Bcl‐xL), an anti‐apoptotic protein, belongs to the Bcl‐2 family. Liu and colleagues found that *L. fermentum* treatment downregulated the expression of Bcl‐x_L_ and then promoted cell apoptosis.^[^ ^65d^ ^]^ B‐cell lymphoma‐2‐associated X‐protein (Bax), a pro‐apoptotic protein, is involved in hastening the process of programmed cell death. The oral administration of *L. plantarum*‐12 significantly ameliorated colon injury in the AOM/DSS‐treated mice by enhancing colonic tight junction protein level and promoting tumor cell death via downregulating PCNA (proliferating cell nuclear antigen) and upregulating the pro‐apoptotic Bax.^[^ ^77^ ^]^ Supplementation of *L. rhamnosus* GG and *L. acidophilus* has been recently shown to contribute to CRC prevention by modifying the gut microenvironment, downregulating the expression of anti‐apoptotic Bcl‐2, proto‐oncogene K‐ras and upregulating pro‐apoptotic Bax as well as tumor suppressor p53.^[^ ^90^ ^]^ Probiotics can also mediate the apoptosis of CRC cells through the phosphoinositide 3‐kinase (PI3K)/protein kinase B (AKT) signaling pathway. Researchers from Zununi Vahed's team found that *Leuconostoc mesenteroides* (*L. mesenteroides*), a dairy‐derived bacterium, suppressed the activation of the AKT pathway and induced a marked rise in the ratio of Bax to Bcl‐xL, as well as enhanced caspase‐3 activity, which resulted in the apoptosis of cancer cells.^[^ ^91^ ^]^ This outcome was consistent with prior research demonstrating that AKT activation promotes Bcl‐2 production while suppressing Bax, consequently lowering the Bax/Bcl‐2 ratio.^[^ ^92^ ^]^ Furthermore, it is known that the elevated expression of Bcl‐2 and Bax can regulate the initiation of caspase‐3, a key enzyme in the apoptotic process, and mediate cell survival or death.^[^ ^23^, ^93^ ^]^

Notably, there are connections between different signaling pathways, such as PI3K/AKT and Wnt pathways.^[^ ^94^ ^]^ *B. lactis SF* enhanced the expression of IL‐10, not only safeguarding the integrity of the intestinal barrier but also preventing the escape of pro‐inflammatory cytokines like transforming growth factor‐β (TGF‐β), thereby inhibiting the PI3K/AKT signaling cascade and suppressing the Wnt signaling pathway, ultimately promoting apoptotic autophagy.^[^ ^23^ ^]^ Researchers have also validated that administrated probiotics can inhibit CRC cell growth via the Notch and Wnt/β‐catenin signaling pathways. The Notch pathway was one of the significant signaling pathways. It could be disrupted during CRC, which was activated at the base of the intestinal crypts and was indispensable for various cellular processes, encompassing normal epithelial cell differentiation, cell‐to‐cell communication, proliferation, and homeostasis. A probiotic mixture consisting of *L. plantarum*, *L. rhamnosus*, *L. brevis 205*, and *L. reuteri* decreased the gene expression of neurogenic locus notch homolog protein (NOTCH), Hes family BHLH transcription factor 1 (HES1), and Musashi RNA binding protein 1 (MSI1) while not affecting the expression of endocytic adaptor protein (NUMB), which inhibited Notch signaling. Concurrently, this probiotic cocktail also decreased the expression of β‐catenin and Cyclin D1 in the Wnt/β‐catenin pathway in a time‐dependent manner.^[^ ^95^ ^]^

The Mitogen‐Activated Protein Kinase (MAPK) /extracellular signal‐regulated kinase (ERK) pathway is significantly involved in a range of biological processes associated with cancer. The impact of MAPK on apoptosis can vary; it may either promote or suppress cell death depending on the specific cell type, the nature of the stimuli, and the variability in the expression and activation levels of different p38 kinase isoforms.^[^ ^96^ ^]^ For example, the probiotic *L. mesenteroides*, isolated from dairy products, promoted CRC cell apoptosis by increasing MAPK1 activity. Additionally, oral administration of *L. plantarum*‐12 has been reported to attenuate CRC in AOM/DSS‐treated mice via inhibiting p38 MAPK signaling pathways.^[^ ^77^ ^]^

Survivin, cIAP1, and XIAP are members of the inhibitor of apoptosis protein (IAP) family. Isazadeh et al. showed that *L. acidophilus* significantly increased smac gene expression levels and decreased survivin gene expression levels in CRC cells. Therefore, *L. acidophilus* enhanced CRC cell death by regulating apoptosis‐related gene expression.^[^ ^97^ ^]^ Spyridopoulou et al. used *L. casei* ATCC 393 to synthesize biogenic selenium nanoparticles. They found SeNp‐treated downregulated Survivin to 0.8‐fold and cIAP1 to 0.6‐fold and upregulated XIAP to 3.8‐fold, which could induce apoptosis and elevate ROS levels in cancer cells.^[^ ^54^ ^]^ The caspase protein family is also a key component of the apoptosis signaling pathway. Once activated by specific apoptotic signals, the Initiator caspase can activate executioner caspase, triggering a cascade of apoptosis. For instance, *L. casei BL23* increased caspase‐7, caspase‐9, and Bik levels, protecting mice from CRC progression.^[^ ^98^ ^]^ Likewise, it was found that *L. casei* elevated apoptotic markers, such as cleaved caspase 3 and poly (ADP‐ribose) polymerase 1 (PARP1) in tumor tissue.^[^ ^81^ ^]^

miRNA‐21 is an antiapoptotic‐miRNA frequently overexpressed in most cancers.^[^ ^99^ ^]^ *L. mesenteroides* significantly reduced miRNA‐21 and miRNA‐200b expression levels.^[^ ^91^ ^]^ Probiotics like *Bifidobacterium longum subsp. Longum* were modulated to enhance tumor‐suppressing miRNAs (miRNA 145, miRNA 15a) and reduce oncogenic miRNAs (miRNA 21a, miRNA 155), thereby inhibiting CRC progression.^[^ ^100^ ^]^

#### 3.1.3 Enhancement of Intestinal Barrier Integrity

The integrity of the intestinal barrier is essential for maintaining gastrointestinal health by preventing the translocation of gut microbiota and luminal contents. Conversely, barrier dysfunction increases intestinal permeability, which promotes bacterial migration and intestinal inflammation, and may ultimately trigger tumorigenesis.^[^ ^101^ ^]^ Goblet cells, which secrete mucin, form a mucosal barrier that protects epithelial cells and prevents the adhesion of pathogenic microorganisms. Regrettably, CRC patients often exhibit reduced production of mucin proteins, which can impair the integrity of the mucosal barrier and contribute to the disease progression.

**Figure 4:**
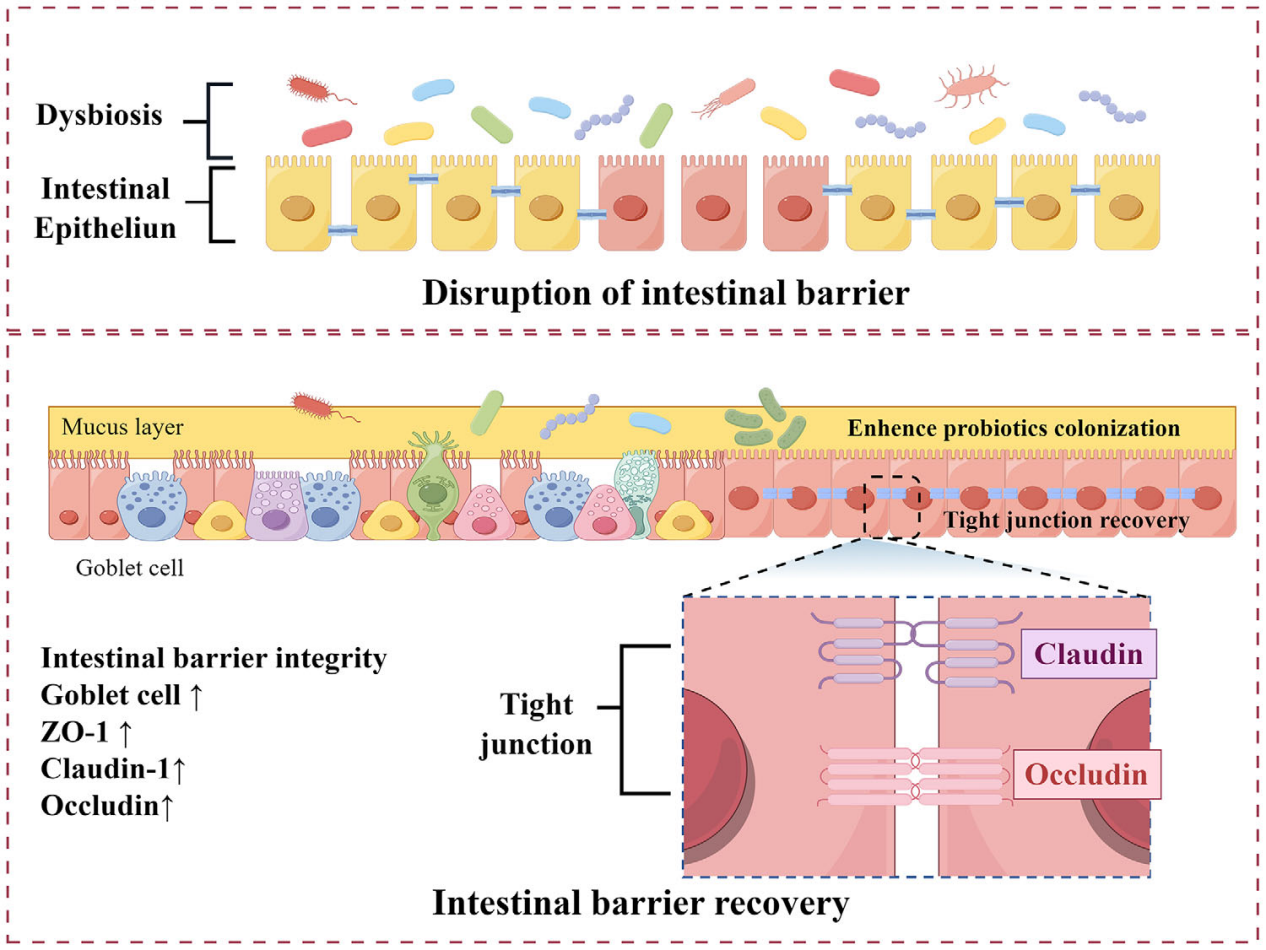
The mechanism of probiotics in repairing intestinal barrier in CRC.

Much research has indicated that probiotics can effectively repair gut barrier damage induced by AOM/DSS, as illustrated in **Figure** **4**. For instance, research conducted by Chen et al. demonstrated that the integrity of the intestinal mucosal barrier could be enhanced through the oral ingestion of commensal bacteria encapsulated with poly(ethylene glycol) (PEG), which promotes their penetration into the mucosal layer. In murine models experiencing an imbalance in intestinal homeostasis, mucus‐penetrating PEGylated bacteria have been shown to exhibit a preferential affinity for the mucosal lining in the distal gastrointestinal tract. These bacteria effectively curtailed the encroachment of pathogenic microorganisms, preserved the equilibrium of the gut microbiota, stimulated mucus production, and triggered the upregulation of tight junction proteins, ultimately averting the onset of colitis and diabetes in rodents.^[^ ^102^ ^]^ Supplementation with *L. plantarum*‐12 not only recovered crypt structure but also increased goblet cell numbers and upregulated the protein expression of Claudin‐1, a key component of tight junctions.^[^ ^77^ ^]^ Treatment with *C. crustorum* MN047 in the AOM/DSS induced CA‐CRC mice preserved goblet cell and increased the mRNA levels of tight junction proteins, thereby preventing pathogen invasion and reducing inflammation.^[^ ^65b^ ^]^ Similarly, *L. rhamnosus* LS8 (LRL) reduced intestinal permeability by protecting goblet cells and increasing the expression of tight junction proteins like Zona occludens 1 (ZO‐1), occludin, and claudin‐1.^[^ ^5c^ ^]^ In mice treated with *R. intestinalis*, the protein expression of tight junction proteins ZO‐1 and claudin‐3 significantly increased in the colon tissues.^[^ ^39^ ^]^ Combined treatment with IL‐2 and AKK effectively maintained intestinal morphology, providing an intact mucosal barrier against infection and colitis.^[^ ^86^ ^]^ *B. bifidum* has been further investigated for its ability to maintain gut barrier function. The research has elucidated that *B. bifidum* mitigated colitis by modulating the expression of occludin and E‐cadherin via nucleotide‐binding oligomerization domain (NOD)‐like receptor family pyrin domain containing 6 (NLRP6)/caspase‐1/IL‐8 signaling pathway, reducing LPS infiltration and subsequent inflammation.^[^ ^103^ ^]^ Therefore, probiotics offer an exciting and promising treatment approach to strengthen intestinal barrier function, reduce the consequential inflammatory response and lower CRC development risk. By targeting the restoration and maintenance of a healthy gut barrier, probiotics have the potential to significantly impact the prevention and treatment of CRC.

#### 3.1.4 Effects of Probiotic‐Derived Metabolites in CRC

Probiotic metabolites promote cell cycle arrest, induce apoptosis, and activate immune responses. Short‐chain fatty acids (SCFAs), especially butyric acid, benefit CRC patients by suppressing the growth of harmful microbes, enhancing mucus secretion and thickening the mucosal layer,^[^ ^104^ ^]^ regulating cellular proliferation,^[^ ^105^ ^]^ inhibiting histone deacetylases,^[^ ^106^ ^]^ promoting the synthesis of tumor suppressors like FAS, p21, and p27,^[^ ^107^ ^]^ and exerting anti‐inflammatory effect.^[^ ^65^, ^108^ ^]^ For instance, *C. butyricum* ATCC 19 398 treatment altered microbial‐derived metabolites such as SCFAs and activated specific butyrate receptors, namely G‐protein coupled receptor 43 (GPR43) and GPR109A. The activation of GPR109A suppressed NF‐κB activation, downregulated cyclin D1, and inhibited cell proliferation, thereby inducing apoptosis.^[^ ^89^ ^]^ Kang et al reported that *R. intestinalis* generated butyrate, which strengthened the efficacy of cytotoxic CD8^+^ T cells by engaging the TLR5‐NF‐κB signaling pathway.^[^ ^39^ ^]^ Besides, butyrate also strengthened tight junctions by activating Amp‐activated protein kinase (AMPK) and preserving intestinal epithelial barrier function.^[^ ^109^ ^]^

Other probiotic metabolites like indole‐3‐carboxylic acid (ICA) modulated antitumor immunity by suppressing Indoleamine 2,3‐dioxygenase (IDO1) expression in tumors and lowering kynurenine production in the tumor microenvironment.^[^ ^84^ ^]^ Interestingly, in another study, *L. gallinarum* was found to convert L‐tryptophan into indole‐3‐lactic acid (ILA), which inhibited CRC cell growth and induced cell apoptosis and patient‐derived CRC organoids.^[^ ^5a^ ^]^ Another study also showed that *L. plantarum* ‐derived ILA accelerated IL‐12 production in dendritic cells, priming CD8^+^ T cell immunity against tumor development and playing a role in the epigenetic modulation of serum amyloid A3 (SAA3) inhibition in cholesterol metabolism within CD8^+^ cells.^[^ ^8a^ ^]^ Postbiotics, which include metabolites, cell‐free supernatants, enzymes, EPS, and other components from probiotics, have been studied extensively.^[^ ^110^ ^]^ *L. acidophilus* lysates inhibited M2 polarization and reduced IL‐10 levels in LPS‐induced RAW 264.7 cells, thereby neutralizing the pro‐inflammatory microenvironment and suppressing tumor progression via limiting monocyte‐derived macrophage accumulation.^[^ ^85^ ^]^ *Akkermansia muciniphila* and Amuc_1100 blunted tumourigenesis by expanding cytotoxic T lymphocytes in the colon and mesenteric lymph nodes, as demonstrated by TNF‐α induction and PD‐1 downregulation.^[^ ^111^ ^]^ Likewise, EPS from several probiotic bacteria has also indicated anti‐cancer potential in studies. EPSs are long‐chain polysaccharides synthesized by microbes, and those derived from LAB have exhibited anti‐cancer effects in CRC cell lines.^[^ ^12^ ^]^ The microbial EPS produced by *Lactobacillus delbrueckii subsp. bulgaricus* OLL1073R‐1 (EPS‐R1) has been shown to enhance the antitumor effects of anti‐CTLA‐4 or anti‐PD‐1 monoclonal antibodies against CCL20‐expressing tumors, with an increase in infiltrating CCR6^+^ CD8^+^ T cells and IFN‐γ production.^[^ ^112^ ^]^ Another research also reported that three exopolysaccharide fractions (EPS1, EPS2, and EPS3) obtained from *Lactobacillus pantheris* TCP102 significantly induced the production of nitric oxide (NO), TNF‐α, and IL‐6 in peritoneal macrophage cells while also suppressing the proliferation of HCT‐116, BCG‐803, and particularly A‐2780 cells.^[^ ^113^ ^]^

In addition to the immune modulation mentioned above, some works have demonstrated that probiotic derivatives can hinder the occurrence and progression of CRC by inducing apoptosis of cancer cells. Bell et al. showed that *L. reuteri* and its metabolite, Reuterin, can combat CRC by causing oxidative stress and hindering protein synthesis.^[^ ^114^ ^]^ They observed an increase in genes crucial for managing oxidative stress (NQO1, GCLM, HMOX1) and genes that function under the control of the nuclear factor erythroid 2–related factor 2 (NRF2) pathway. Ferrichrome, the compound derived from the < 3kDa fraction of *L. casei* ATCC334 culture fluid is a siderophore that sequesters metals and triggers endoplasmic reticulum (ER) stress responses. This leads to selective apoptosis in CRC cells via the c‐jun N‐terminal kinase (JNK)‐CHOP pathway and suppresses CRC xenograft growth in mouse models.^[^ ^115^ ^]^ Additionally, the therapeutic protein p8, derived from *L. rhamnosus*, has been shown to possess potent, strong anti‐proliferative effects in a mouse CRC xenograft model by specifically entering the cytosol of DLD‐1 cells and reducing the levels of Cyclin B1 and CDK1, both of which are pivotal for cell cycle advancement.^[^ ^43^ ^]^

### 3.2 The Mediated Role of Gut Microbiota in CRC

Gut microbiota plays a crucial role in CRC development, which could interact with colonic epithelial cells and immune cells through metabolites and proteins.^[^ ^116^ ^]^ Microbial community dysbiosis weakens the intestinal barrier, which in turn allows for the leakage of microbes and their metabolites. This breach can initiate persistent inflammation, disrupt lipid metabolism, disturb the normal growth patterns of cells, and hinder the capacity of myeloid cells to eliminate mutant, senescent, and malfunctioning cells. These effects can cumulatively contribute to the promotion of tumor development.^[^ ^117^ ^]^ Unfortunately, imbalances in gut microbiota are commonly observed in CRC patients. Studies have confirmed that certain bacteria, like *Fusobacterium nucleatum*, *Streptococcus gallolyticus*, *Escherichia coli*, *Enterococcus faecalis*, and *Bacteroides fragilis* (*B. fragilis*), are more prevalent in CRC patients than in healthy people. Conversely, the presence of beneficial bacteria, such as *Clostridium*, *Faecalibacterium*, *Roseburia*, and *Bifidobacterium* tends to be decreased in these patients.^[^ ^77^ ^]^

**Figure 5:**
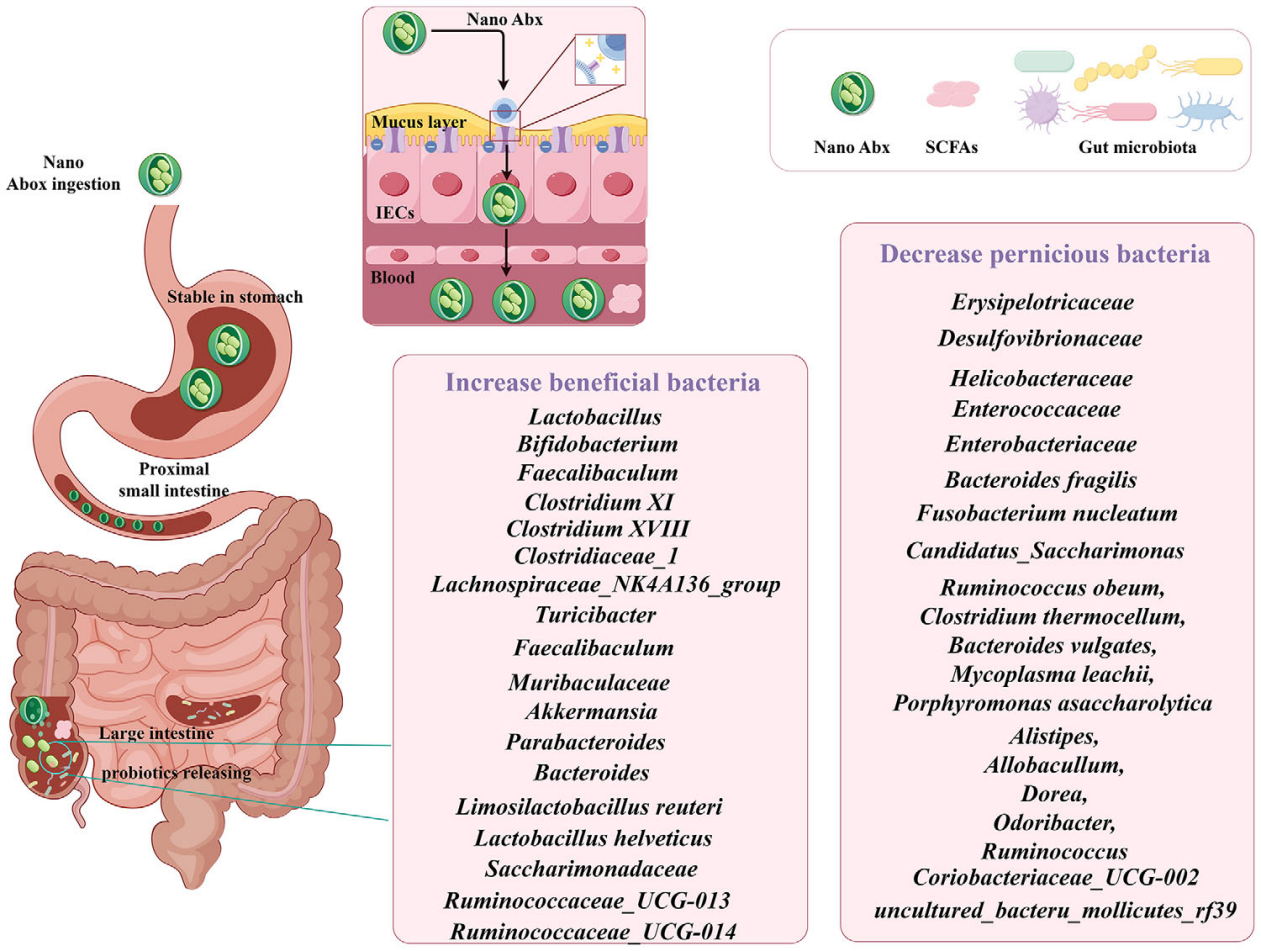
The regulation mechanism of gut microbiota by probiotics in CRC.

In light of these findings, a growing body of research is now focusing on the application of probiotics to regulate gut microbiota. Probiotics have been shown to facilitate the restoration of microbial equilibrium within the gastrointestinal tract, enhancing beneficial bacteria and inhibiting CRC‐associated bacteria growth (**Figure** **5**). They modulate the Firmicutes/Bacteroidetes ratio, crucial for gut homeostasis.^[^ ^118^ ^]^ For instance, it was confirmed that *Faecalibacterium prausnitzii* treatment increased the abundance of Firmicutes and lowered the abundance of Bacteroidetes.^[^ ^88^ ^]^ Similarly, a supplement of *L. coryniformis* MXJ32 was found to decrease the abundance of Bacteroidetes and Proteobacteria, while increasing the abundance of Actinobacteria.^[^ ^119^ ^]^ Interestingly, the Firmicutes/Bacteroidetes ratio in the *Bifidobacterium animalis subsp. lactis SF* groups was also significantly higher than in the CPT‐11 group.^[^ ^23^ ^]^ Yet, accumulating evidence pointed out a decrease in the ratio of Firmicutes/Bacteroidetes, which was also beneficial in inhibiting CRC. Oral administration of *L. plantarum*‐12, for example, reduced the relative abundance of Firmicutes and increased the relative abundance of Bacteroidetes in the AOM/DSS‐treated mice.^[^ ^77^ ^]^ Similarly, a reduction of Firmicutes and an increase in Actinobacteria were observed in mice gavaged with *Streptococcus thermophilus*.^[^ ^119^ ^]^ The relative abundance of Firmicutes and Bacteroidetes is influenced by many factors such as age and lifestyle and thus cannot be generically defined. Given the complexity of the gut microbiota ecosystem, a simple division at the phyla level only provides a basic clue. For a more accurate analysis, it is necessary to focus on the most important core bacteria genera, such as common harmful bacteria and a large number of functional bacteria.

Reports have indicated that various probiotics, including LRL‐treated,^[^ ^5c^ ^]^ *L. plantarum*‐12‐treated,^[^ ^77^ ^]^ *L. casei* JY300‐8‐treated,^[^ ^36^ ^]^ *Streptococcus thermophilus*‐treated,^[^ ^119^ ^]^ *L. coryniformis* MXJ32‐treated,^[^ ^65a^ ^]^ *L. gallinarum*‐treated,^[^ ^5a^ ^]^ and *L. acidophilus* 878‐treated,^[^ ^120^ ^]^ generated an increase in beneficial bacteria of *Bifidobacterium*, *Lactobacillus*, *L. helveticus* and *L. reuteri*, which inhibited carcinogen‐induced colorectal tumorigenesis in rodents. Compelling data suggested that *Bifidobacterium* and *Lactobacillus* not only enhance epithelial barrier function but also play a critical role in modulating short‐chain fatty metabolites as well as the cancer response to immunotherapy.^[^ ^121^ ^]^ For instance, *L. rhamnosus* Probio‐M9 regulated gut microbiota in tumor‐bearing mice by enriching the presence of strains that produce SCFAs (e.g.*, L. reuteri*, *L. murinus*, *L. johnsonii*, *Staphylococcus lentus*, *Enterococcus gallinarum*). This enrichment led to higher acetic, propionic, and butyric acid levels in the gastrointestinal tract. The SCFA‐producing bacteria also elevated the levels of certain metabolites in the mice's bloodstream, including α‐ketoglutarate (α‐KG), N‐acetyl‐L‐glutamic acid, and pyridoxine, which are particularly important for enhancing the activity and infiltration of CTLs and for dampening the activity of regulatory T cells (Tregs) within the tumor microenvironment (TME). Notably, α‐KG has been found to influence the gut microbiota composition, further boosting the population of *Lactobacillus* and the production of SCFAs.^[^ ^34^, ^122^ ^]^ Thus, enhancing SCFAs formation through the regulation of gut microbiota holds promise as a future strategy in anti‐cancer therapy.

Apart from the specifically mentioned *Lactobacillus* and *Bifidobacterium*, some other bacteria from the Firmicutes phylum (e.g.*, Clostridium* and *Faecalibaculum*) as well as some next‐generation probiotics have also shown beneficial effects. For example, it was proposed in previous studies that *C. butyricum* were butyrate‐producing bacteria that decreased CRC cell proliferation and apoptosis as well as decreased pathogenic bacteria and increased SCFAs‐producing bacteria.^[^ ^89^ ^]^ *Akkermansia muciniphila* were important in inducing antigen‐specific T cell responses, which helped maintain host immune homeostasis.^[^ ^123^ ^]^ The presence of these bacteria increases in the gut with probiotics treatment. Mendes et al. highlighted the statistically significant difference at the genus level, noting an increase in *Clostridium XI*, *Clostridium XVIII*, *Akkermansia*, and *Allobaculum* when supplemented with *L. acidophilus*, *L. rhamnosus*, and *Bifidobacterium bifidum*.^[^ ^67^ ^]^ Similarly, *L. plantarum* L168 and ILA administration upregulated the abundance of *Faecalibaculum* and *Bifidobacterium*.^[^ ^8a^ ^]^ A new strain of *L. lactis*, named ‘HkyuLL 10′ and successfully isolated from healthy human stools, has demonstrated the ability to suppress CRC tumorigenesis in mice. This is achieved by enriching the gut with commensal probiotics such as *Propionibacterium freudenreichii*, *Akkermansia muciniphila*, *L. johnsonii*, *L. intestinalis*, and *L. reuteri* and by secreting α‐mannosidase (αMAN).^[^ ^124^ ^]^ The interplay between the administered probiotics and the augmented commensal bacteria has the potential to exert a synergistic effect with the anticarcinogenic properties of the probiotics.

Likewise, the administration of *L. rhamnosus* Probio‐M9 has led to a significant increase in the relative abundance of beneficial bacteria within the Bacteroidetes phylum, such as *Bacteroides intestinalis* (*B. intestinalis*) and *Bacteroides xylanisolvens* (*B. xylanisolvens*), both of which were recognized for their positive impact on the host's health.^[^ ^125^ ^]^ *B. intestinalis*, in particular, can enhance host immunity by producing metabolites or inducing the transcription of interleukin IL‐1β,^[^ ^126^ ^]^ while *B. xylanisolvens* has been positively correlated with cancer treatment outcomes.^[^ ^127^ ^]^ Similarly, another study found that *L. rhamnosus* Probio‐M9 administration significantly increased the relative abundance of beneficial bacteria (e.g., *Bifidobacterium pseudolongum*, *Parabacteroides distasonis*, and some Bacteroides species).^[^ ^128^ ^]^ Treatment with *C. crustorum* MN047 could significantly increase the abundance of *Akkermansia*, *Parabacteroides*, *Clostridium_sensu_stricto_1*, *Bacteroides*, and *Ruminococcaceae_UCG‐013*.^[^ ^65b^ ^]^ Consistent with these findings, the contents of beneficial families like Muribaculaceae, Clostridiaceae_1, and Saccharimonadaceae in the gut microbiota of mice treated with AOM/DSS were elevated by *L. plantarum*‐12.^[^ ^77^ ^]^ Besides, multiple studies have shown the advantageous impacts of non‐toxigenic *B. fragilis* strains on enhancing the host's immune response to immunotherapy, suppressing tumorigenesis, and mitigating inflammation through the secretion of polysaccharides.^[^ ^129^ ^]^ Bacteroides species are capable of decomposing polysaccharides into oligosaccharides or SCFAs. SCFAs‐producing bacteria can increase the concentration of acetic acid, butyric acid, and propionic acid in the intestinal cavity by fermenting oligosaccharides and monosaccharides.^[^ ^130^ ^]^ Thus, the proliferation of beneficial Bacteroides can help maintain a healthy equilibrium within the gut microbiota.

However, some Firmicutes and Proteobacteria are potentially pathogenic. Probiotics reduce harmful bacteria like Erysipelotrichaceae and Desulfovibrionaceae, which are associated with inflammation and CRC risk.^[^ ^131^ ^]^ For example, the relative abundances of Erysipelotrichaceae, a potentially pathogenic bacterial family, significantly decreased after *L. plantarum* 12 administration  .^[^ ^77^ ^]^ Similarly, LRL‐treated could decrease *Ruminococcaceae_UCG‐014* and *Turicibacter* at the genus level.^[^ ^5c^ ^]^ Gao et al. also reported that *Mucispirillum*, *Candidatus Arthromitus*, and *Clostridium* decreased after supplementing with *L. rhamnosus* Probio‐M9.^[^ ^34^ ^]^ These microbial alterations were accompanied by a reduced incidence of colitis, less colonic inflammation, as well as lower expression of inhibitors such as IKKβ and TNF‐α. Besides, some Proteobacteria bacteria, such as Desulfovibrionaceae, which are sulfate‐reducing and endotoxin‐producing, may be associated with an increased risk of CRC by promoting oxidative stress, DNA damage, and hyper‐proliferation in intestinal epithelial cells.^[^ ^132^ ^]^ *L. plantarum*‐12 treatment reduced the relative abundances of Helicobacteraceae and Desulfovibrionaceae.^[^ ^77^ ^]^ Likely, the supplementation of *L. coryniformis* MXJ32, decreased the abundance of *Desulfovibrio* and *Helicobacter* as well, attenuating the overexpression of inflammation and significantly increasing the level of Isovaleric and other SCFAs (e.g., Propionic, butyric acid and isobutyric), which were partly beneficial to the anti‐carcinogenic effect.^[^ ^65a^ ^]^

Additionally, certain bacteria within the *Bacteroides* phylum can be potentially pathogenic, such as Enterotoxigenic *B. fragilis* (ETBF) and *Bacteroides vulgates*. Researchers reported that ETBF strongly induced colonic tumors in mice via promoting chronic inflammation and altering microbial composition at the colorectal site.^[^ ^133^ ^]^ Fortunately, these potential pathogenic could be reduced with the treatment of probiotics. For instance, *L. rhamnosus* Probio‐M9‐treated could decrease *Bacteroides*.^[^ ^5^, ^34^ ^]^ And prolonged administration of *L. acidophilus* 878 could reduce the development of colorectal tumors in rats by modifying intestinal pathogenic bacteria, including *Bacteroides vulgates* and *Porphyromonas asaccharolytica*.^[^ ^120^ ^]^ Furthermore, it was discovered that *Fusobacterium nucleatum* potentiated intestinal tumorigenesis in Apc^Min/+^ mice via a TLR4/p‐PAK1/p‐β‐catenin S675 signaling pathway.^[^ ^134^ ^]^ However, a novel probiotic formula consisting of *B. adolescentis*, *B. longum*, and *B. bifidum* effectively inhibited the growth of *Fusobacterium nucleatum* and improved the gut microbial environment against CRC development.^[^ ^135^ ^]^ These results suggested that probiotic intervention enhanced the antitumor abilities by fostering the growth of beneficial bacteria while suppressing the harmful ones in antibiotic‐treated tumor bearing mice.^[^ ^128^ ^]^

## 4 Targeted Delivery Systems for Probiotics

As mentioned above, probiotics have potential health benefits. However, the delivery of probiotics through the gastrointestinal tract faces various challenges, such as harsh gastrointestinal environments. Encapsulation emerges as a highly effective method for maintaining the vitality of probiotics.^[^ ^136^ ^]^ Extensive research has been conducted to create various encapsulation methods for probiotics, such as nano armors,^[^ ^137^ ^]^ biofilms,^[^ ^138^ ^]^ gel microspheres,^[^ ^139^ ^]^ liposomes,^[^ ^140^ ^]^ probiotic spores,^[^ ^141^ ^]^ and so on. However, conventional encapsulation often falls short in safeguarding probiotics from the harsh conditions of the gastrointestinal tract, due to factors such as acidic gastric juices, bile salts, and digestive enzymes.^[^ ^136a^ ^]^ For instance, biopolymer microgels possess a high degree of porosity, which permits the infiltration of gastric acids and enzymes, leading to the potential degradation of the encapsulated probiotics. Spray drying encompasses atomizing a bacterial suspension into small droplets and then rapidly evaporating the water using hot air (up to 200 °C). During this process, microorganisms are exposed to diverse stresses such as thermal, dehydration, shear, and osmotic stresses, which collectively can reduce probiotic viability.^[^ ^136a^ ^]^ And probiotics are unable to be released within the colon or adhere to the colon's lining and be colonized on it, leading to rapidly passing through the human body and cannot exert their effects.^[^ ^142^ ^]^

More recently, there has been a shift in research emphasis toward improving the adhesion of probiotics within the target site.^[^ ^143^ ^]^ The ability of delivery systems to adhere to the target area is crucial for extending the duration of probiotic residence at the specified site in the gastrointestinal tract (GIT), which in turn optimizes their potential to deliver health benefits. Researchers are also committed to improving targeting and the survival rate of probiotics. This section systematically summarizes recent advances in the targeted delivery of probiotics for CRC prevention and treatment. These oral delivery systems are specifically designed to improve probiotic viability and mucosal adhesion, ensure effective colonic colonization, and provide site‐specific release in the gut.

However, based on our understanding, the existing research predominantly addresses targeted delivery therapy for colitis, whereas the focus on CRC therapy is relatively minor. Several clinical and epidemiological studies suggest that chronic inflammation serves as a pivotal initiating element in the pathogenesis of colorectal tumors, leading to DNA damage, impaired gut barrier function, and immunosuppression.^[^ ^144^ ^]^ For example, IBD, including ulcerative colitis (UC) and Crohn's disease (CD), poses a serious hazard to the development of colitis‐associated CRC. It was reported that individuals with UC have a four to twenty times higher incidence of cancer.^[^ ^145^ ^]^ The likelihood of developing inflammatory CRC is strongly correlated with the chronicity of colon inflammation and the extent of the lesions; the longer the inflammatory process and the broader the affected area, the higher the risk of cancer development.^[^ ^146^ ^]^ Therefore, improving intestinal inflammatory responses has become an effective strategy to prevent CAC. In light of these findings, we have also reviewed the literature on targeted delivery of probiotics to enhance the efficacy of IBD treatment in this article. Our aim is to provide guidance and insights that may inform future targeted delivery of probiotics for CRC prevention and treatment.

### 4.1 Microencapsulation Technology in Probiotics Delivery

Conventionally, predominant encapsulation methodologies employed for safeguarding probiotic cells have centered on microencapsulation technologies, whereby probiotics are embedded within micron‐scale gel networks, particles, or emulsion microgels prior to administration.^[^ ^147^ ^]^ The fabrication of these microgels commonly employs biopolymeric substances, such as starch, alginate, carrageenan, gelatin, xanthan gum, and proteins. These materials are chosen for their favorable properties, such as thermal stability, biocompatibility, minimal toxicity, and affordability.^[^ ^148^ ^]^ However, conventional microencapsulation was unable to control the dimensions of microgels, which was closely related to the survival of probiotics. Meanwhile, the viability and bioavailability of probiotics in vivo efficiency were so low that only a few probiotics could target tumor tissue. Thus, recent studies have focused on reducing microgel size to ensure efficient transit through the GIT, achieve targeted colon delivery, and enhance probiotic viability.

#### 4.1.1 Microgel Systems

Alginate has become a popular choice for probiotic encapsulation due to its water absorption capacity and ability to form gels through ionic cross‐linking. The alginate‐ion interaction generates hydrogels that resist harsh gastric conditions, particularly in the stomach.^[^ ^149^ ^]^ Moreover, alginate remains undigested within the upper gastrointestinal tract, whereas it undergoes fermentation in the lower gastrointestinal tract (specifically the colon) due to the enzymatic action of colonic bacteria.^[^ ^150^ ^]^ This property facilitates its ability to encapsulate and retain probiotics in the upper gastrointestinal tract, subsequently releasing them upon reaching the lower gastrointestinal tract.^[^ ^151^ ^]^

**Figure 6:**
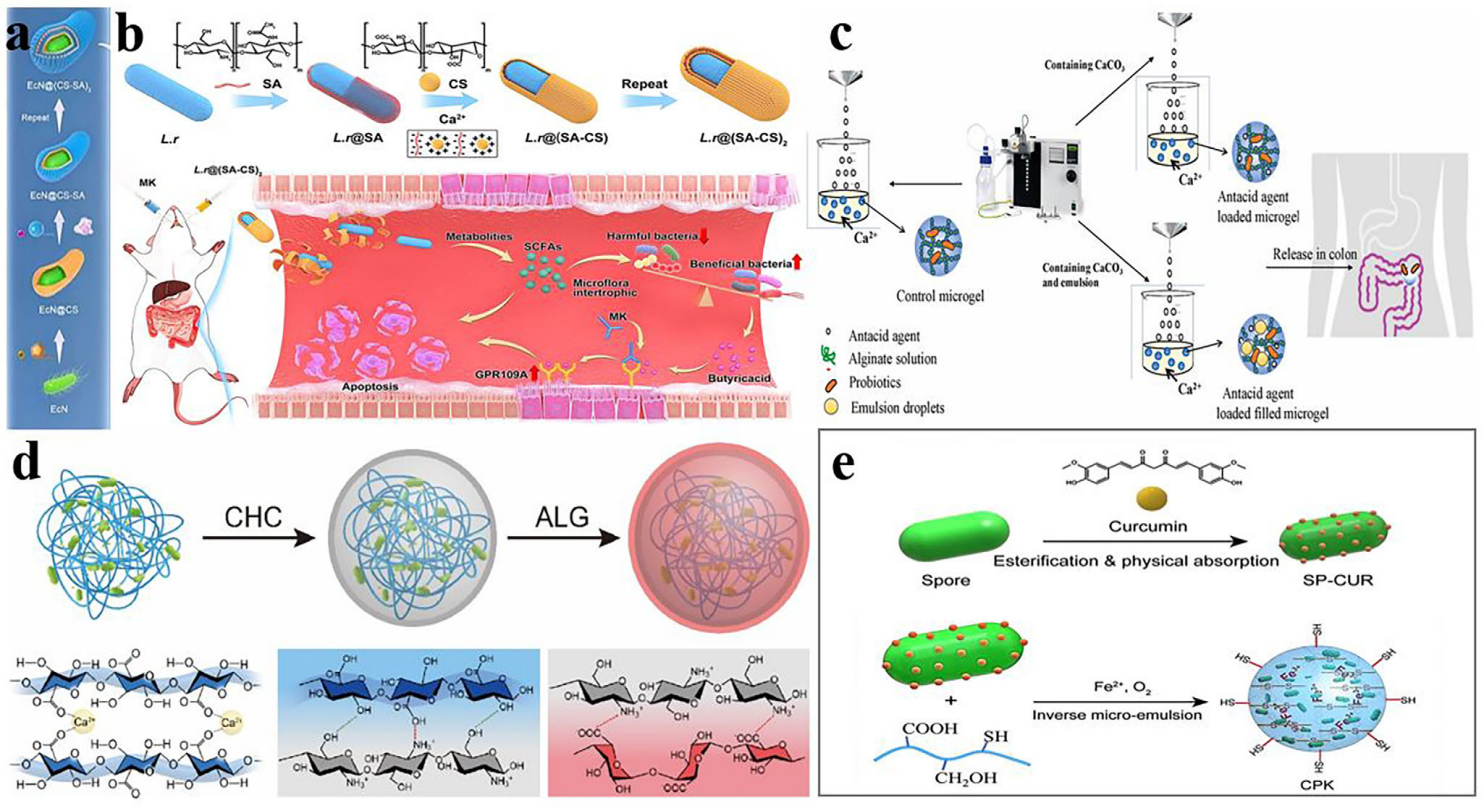
Microencapsulation of probiotics in microgels systems. a) Schematic illustration of *EcN*@(CS‐SA)_2_ microgel through layer‐by‐layer assembly and crosslinking with calcium chloride ions. Reproduced with permission.^[^ ^153^ ^]^ Copyright 2024, Elsevier B.V. b) Schematic illustration for the preparation of *L.r*@(SA‐CS)_2_ by encapsulating *L.r* with SA and CS through the layer‐by‐layer strategy.^[^ ^154^ ^]^ c) Schematic diagram of fabrication of probiotic‐loaded microgels: control microgel; antacid‐microgels; and, antacid‐nE microgels. Reproduced with permission.^[^ ^151^ ^]^ Copyright 2021, Elsevier Ltd. d) Schematic illustration of the procedure of preparing the single‐layer gel and multi‐layer gel. Reproduced with permission.^[^ ^159^ ^]^ Copyright 2023, Elsevier Ltd. e) Illustration of decoration procedure of curcumin onto bacterial spores and the encapsulation process of SP‐CUR by SOKGM through crosslinking of carboxyl‐Fe^3^^+^ coordination and disulfide bonds formation. Reproduced with permission.^[^ ^157^ ^]^ Copyright 2023, Elsevier Ltd.

SA microbeads are widely utilized for encapsulating, protecting, and delivering probiotics due to their high viscosity, which is beneficial for sustained release. However, the high porosity and bulkiness of SA microbeads can prematurely release the encapsulated probiotics. Research has demonstrated that the combination of SA and CS created chelation with EDTA, which not only supported the survival of *EcN* but also improved its adhesive properties and mechanical strength.^[^ ^58^, ^152^ ^]^ For instance, Niu and colleagues discovered that SA and CS together formed a chelating agent EDTA that created an environment conducive to the survival of *EcN*, and endowed it with better adhesion properties and mechanical strength (**Figure** **6**a). This oral microgel delivery system *EcN*@(CS‐SA)_2_ not only sustained 99.6% viability of the encapsulated bacteria but also withstood the exposure to simulated gastric fluid (SGF) for 4 hours, thereby exemplifying pronounced gastrointestinal resilience.^[^ ^153^ ^]^ Moreover, this microgel potentiated the antineoplastic efficacy of Galunisertib (Gal), an inhibitor of TGF‐β, by inducing apoptosis and immunogenic cell death (ICD) within malignant cells, as well as by enhancing the infiltration of CD8^+^ T lymphocytes into the TME, in a synergistic manner. It also significantly enhanced the abundance of *Lactobacillus*, *Akkermansia*, and *Bifidobacterium* in the intestinal microbiota.^[^ ^187^ ^]^ Another study also reported on a multilayer microgel encapsulation system L. r@(SA‐CS)_2_, which leveraged the electrostatic interactions between SA and CS, along with layer‐by‐layer assembly and calcium chloride ion crosslinking, to shield *L. reuteri* from gastric acid and ensure its arrival in the intestine (Figure 6b). The SCFAs produced by *L. reuteri* can cause apoptosis in some tumor cells, regulate intestinal microbiota by reducing harmful bacteria (e.g.*, Proteobacteria and Fusobacteriota*) and nourishing probiotics that produce butyric acid, and finally enhance antitumor therapeutic effect.^[^ ^154^ ^]^

Another interesting approach recently developed involved the use of calcium alginate to improve the viability of *Bifidobacterium pseudocatenulatum* G7 (BPG7) under gastric conditions, which can control the pore size and internal pH of microgels. Due to highly porous calcium alginate microgels, Zhang and colleagues incorporated colloidal antacids and lipid droplets into calcium alginate microgels. The stability of the probiotics was greatly improved by antacid microgels, which may be due to their ability to maintain a neutral internal pH under simulated stomach conditions (Figure 6c). After exposure to the GIT conditions, no viable probiotic cells were found in the control microgels. In contrast, microgels with CaCO_3_ contained 5.6 log_10_ CFU of live probiotics, and those with both CaCO_3_ and nanoemulsions retained 6.6 log_10_ CFU. The observed outcomes are primarily ascribable to the capacity of colloidal CaCO_3_ particles and lipid droplets to obstruct the migration of enzymes and bile salts into the core of the microgels, achieved by occupying a portion of the interstitial spaces in the calcium alginate network.^[^ ^151^ ^]^

In addition, some research not only preserved the inherent properties of probiotics but also endowed them with additional functionalities to enhance their therapeutic potential. Deol et al. encapsulated a probiotic‐ginger extract (GE), recognized for its antioxidant and potent anti‐inflammatory activity,^[^ ^155^ ^]^ along with *L. acidophilus* into calcium‐alginate beads.^[^ ^156^ ^]^ *L. acidophilus* MTCC5401 is noted for its antioxidant capabilities. To address the poor encapsulation of probiotics due to the numerous fine voids formed in the alginate gel, they employed polyethylene glycol for its hydrophilic, thickening, and osmotic characteristics. Furthermore, the system was coated with Eudragit‐S100 to ensure colon‐specific delivery. In vivo evaluation indicated both GE and LAB could attenuate oxidative stress (catalase, SOD, LPO) and inflammatory burden (IL‐6 and TNF‐), and downregulate COX‐2, iNOS, and c‐Myc. Another study encapsulated anti‐inflammatory curcumin with probiotic *Bacillus subtilis* (*B. subtilis*) spores in cysteine‐modified konjac glucomannan microspheres for the treatment of colitis and the prevention of colonic dysplasia by modulating the gut microbiota (Figure 6e). The polysaccharide microspheres, which leverage electrostatic interactions, have a prolonged retention effect on the intestines. This property effectively restores oxygenation to hypoxic areas of the colon, a process triggered by oxygen consumption during spore germination. As a result, the overgrowth of luminal Enterobacteriaceae is curbed, and the growth of bacteria that produce SCFAs is promoted. The fermentation of konjac glucomannan prebiotics by these bacteria results in the production of butyrate, which activates the peroxisome proliferator‐activated receptor‐c (PPAR‐c) signaling pathway in epithelial cells, shifting cellular metabolism from glycolysis to oxygen‐consuming β‐oxidation. This metabolic shift led to oxygen depletion, decreased nitrate‐producing enzymes, and reduced inflammation. Thus, the developed spore‐laden polysaccharide microspheres have demonstrated significant therapeutic benefits against DSS‐induced colitis‐associated CRC. The combination of natural anti‐inflammatory agents with probiotics presents a novel approach for therapeutic intervention in diseases.^[^ ^157^ ^]^

Besides normally used alginate, cellulose has emerged as a candidate material for probiotic delivery owing to its affordability, biocompatibility, and edibility. Previous research has shown that regenerated cellulose microgels maintain stability in simulated gastric and intestinal environments, characterized by their high porosity and adjustable pore sizes.^[^ ^158^ ^]^ Thus, Luan et al. developed an electrostatically reinforced and sealed nanocellulose‐based microsphere (Figure 6d). The microsphere's porous core offers a spacious and separate habitat for probiotics. The incorporation of CS hydrochloride (CHC) and alginate (ALG) fortified the microsphere's structure, creating a multi‐layered system with pH‐responsive capabilities. These engineered multi‐layer macrospheres are designed to shield probiotics against the corrosive effects of gastric acid and bile salts. Moreover, the outer shell's solubility and the inner porous framework's stability in the intestinal tract promote a controlled release of probiotics in targeted environments.^[^ ^159^ ^]^

#### 4.1.2 Hydrogels Systems

**Figure 7:**
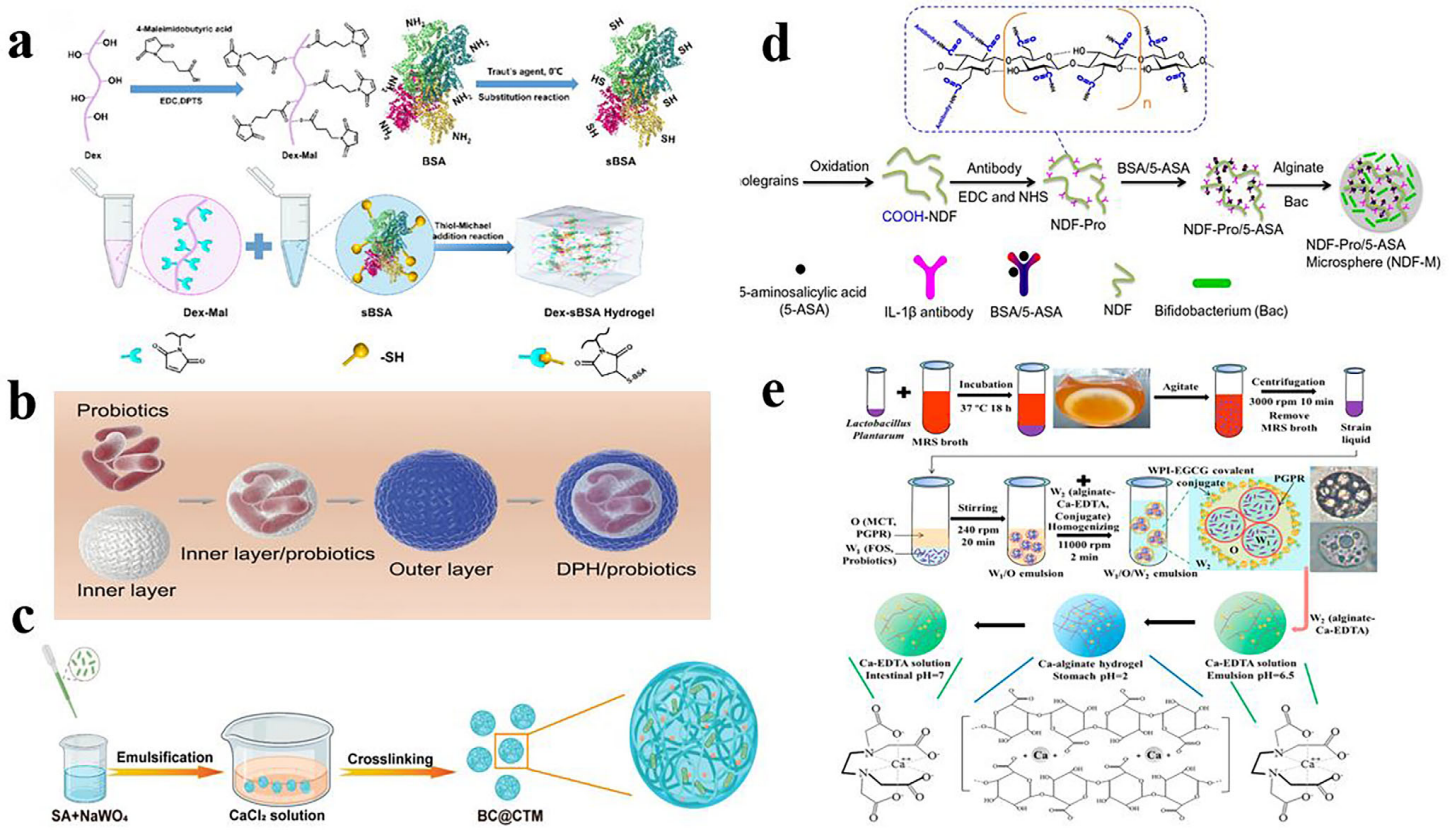
Microencapsulation of probiotics in hydrogel systems. a) Schematic illustration of the synthesis of the dextran (Dex)‐Mal backbone and thiolated bovine serum albumin (sBSA)thiolated bovine serum albumin (sBSA) and Dex‐sBSA hydrogel preparation. Reproduced with permission.^[^ ^162^ ^]^ Copyright 2023, American Chemical Society. b) Schematics of the double‐layer polysaccharide hydrogel (DPH).^[^ ^163^ ^]^ c) Calcium tungstate microgel (CTM) was loaded with BC via CaCl_2_ solution crosslinking using a w/o emulsion method.^[^ ^167^ ^]^ d) Preparation of an nanoscale dietary fibers (NDF)‐Pro/5‐aminosalicylic acid (5‐ASA) composite microsphere (NDF‐M).^[^ ^168^ ^]^ e) The process flowchart for encapsulation and colon‐targeted release of Lactobacillus Plantarum in W_1_/O/W_2_ double emulsions based on alginate‐CaEDTA system. Reproduced with permission.^[^ ^165^ ^]^ Copyright 2020, Elsevier Ltd.

It has been documented that hydrogels made from a single material, such as polysaccharide or protein, face limitations like poor mechanical stability, rapid degradation within the body, and sensitivity to gastric acid.^[^ ^160^ ^]^ In contrast, composite hydrogels combining polysaccharides and proteins offer improved mechanical stability and controlled release in response to stimuli.^[^ ^161^ ^]^ Qiu and colleagues used dextran and thiolated bovine serum albumin (sBSA) to design polysaccharide−protein hydrogels (Dex‐sBSA hydrogels) (**Figure** **7**a). This hydrogel significantly enhanced the survival of probiotic bacteria by several orders of magnitude in SGF and bile salts, and even more in GIF, protecting them from ROS and antibiotics. In vivo experiments have elucidated that encapsulation within a hydrogel matrix significantly enhances the bioavailability of orally administered probiotics. Notably, the cellular count within the intestine and colon was markedly elevated in comparison to the control group without encapsulation. Moreover, the hydrogel extended the residence time of the probiotics in the gut, thereby creating favorable conditions for the colonization and growth of probiotics.^[^ ^162^ ^]^ And Huang et al. introduced a double‐layer polysaccharide hydrogel (DPH) with a structure consisting of a carboxymethyl cellulose (CMCL) inner layer and a dialdehyde alginate (DAA) cross‐linked carboxymethyl chitosan (CMCS) outer layer (Figure 7b). The bifurcated structural design of DPH facilitates the encapsulation and targeted delivery of probiotics within the corporeal environment. Within the gastric cavity, the encapsulating matrix of DPH adopting a cage‐like configuration effectively sequesters probiotics, while the external stratum assimilates adjacent fluids to create a protective barrier against gastric secretion. Subsequently, the cage‐like matrix disassembles in the intestinal lumen, thereby facilitating the release of probiotics. Notably, probiotics encapsulated by DPH showed a 100.1‐fold increase in bioavailability and 10.6‐fold increase in mucoadhesion compared to free probiotics in an animal model 48 h after treatment. Thus, DPH confers probiotics with exceptional intestinal targeting efficacy, augmented oral bioavailability, increased tolerance to the gastrointestinal tract, and a strong mucoadhesive property.^[^ ^163^ ^]^

In order to facilitate the precise delivery of probiotics to the gastrointestinal tract, pH‐sensitive hydrogels are deemed an optimal conveyance for oral administration of probiotics.^[^ ^164^ ^]^ For instance, a W_1_/O/W_2_ double emulsion, stabilized by the whey protein isolate (WPI) and epigallocatechin‐3‐gallate (EGCG) (WPI‐EGCG) covalent conjugate nanoparticles in conjunction with an alginate‐Ca‐EDTA complex, was employed for the encapsulation of *L. plantarum* strain in liquid form. In acidic conditions, alginate and Ca^2+^ form a gel (Figure 7e), while EDTA competes with Ca^2+^ to maintain the system in a liquid state at neutral pH. This double emulsion serves as a colon‐targeted release vehicle for the L. plantarum strain contained in the inner aqueous phase.^[^ ^165^ ^]^ In another study, Ding et al. developed a similar W_1_/O/W_2_ double emulsion encapsulated within a calcium‐alginate hydrogel bead system (ACGs) for the intestinal‐targeted delivery of probiotics. They created carboxymethyl konjac glucomannan‐chitosan (CMKGM‐CS) nanogels to stabilize the W_1_/O/W_2_ double emulsions, which were subsequently encapsulated in alginate to form ACGs hydrogel beads for the targeted delivery of *L. reuteri*.^[^ ^152a^ ^]^ In vitro probiotic release experiments showed the lyophilized ACG‐2 and ACG‐3 hydrogel beads maintained a prolonged release profile within the simulated intestinal fluid (SIF). The cellular viability remained above 10^7^ CFU·mL^−1^ after 6 h. Furthermore, the alginate concentration in ACG hydrogel beads significantly affected their swelling behavior and structural integrity by influencing hydrogen bonding between alginate and CMKGM‐CS, thereby controlling probiotic release kinetics.

In addition, the ability of probiotics to deliver effectively and colonize at their targets was often impeded by the abnormal colonization of Enterobacteriaceae at pathological sites. To overcome this challenge, Yang and colleagues put forth a novel oral probiotic delivery system, which was based on calcium tungstate microgel (CTM) (Figure 7c). Their findings revealed that calprotectin (CP), which was highly prominent within colitis‐affected regions,^[^ ^166^ ^]^ initiated the liberation of tungsten from the CTM through a process of calcium depletion, effectively suppressing the proliferation of Enterobacteriaceae. This effect was mediated by replacing molybdenum in the molybdenum pterin cofactor, without adversely impacting the delivered probiotics. Moreover, CTM demonstrated remarkable adaptability within the inhospitable milieu of the GIT and manifested a strong affinity for intestinal adherence. The synergistic outcome of diminishing Enterobacteriaceae by 45‐fold and augmenting probiotic settlement by a factor of 25 has proven to be pivotal in the management of colitis. This therapeutic strategy has led to significant improvements such as the restoration of colon length, suppression of inflammation, repair of the mucosal barrier, and the reestablishment of gut microbiota balance.^[^ ^167^ ^]^ Another study exploited the advantages of dietary fibers (DFs) and gut microbiota by fabricating an alginate hydrogel microsphere, which served as a vehicle for encapsulating *Bifidobacterium* (Bac) alongside drug‐functionalized nanoscale dietary fibers (NDFs) (Figure 7d). The NDFs were modified with IL‐1β antibodies and a combination of thiolated bovine serum albumin (sBSA) and 5‐aminosalicylic acid (5‐ASA) to create NDF‐Pro/5‐ASA. Subsequently, NDF‐Pro/5‐ASA and Bac were combined and encapsulated into a hydrogel microsphere (NDFM) using an electrostatic droplet generator in the presence of alginate. Upon traversing the colorectal region, the anaerobic fermentation of Bac, which metabolizes NDFs and proteins as carbon and nitrogen substrates, can facilitate the release of therapeutic agents and to augment the efficacy of the probiotic within the gut microbiota. Notably, this innovative system significantly enhanced SCFA production and promoted 5‐ASA release at inflammation sites in murine models of chronic colitis, thereby mitigating gut inflammation and restructuring gut microbiota.^[^ ^168^ ^]^

### 4.2 Nanoencapsulation Technology in Probiotics Delivery

In recent years, there has been a growing body of research utilizing nanomaterials and nanotechnology for probiotics delivery. Nanoencapsulation technologies encase probiotics within a nanoshell matrix such as single‐cell encapsulation via nanocoating. These methods can overcome conventional delivery challenges and offer unique benefits, including enhanced colonization, strong gastric resistance, and significant roles in the prevention and treatment of diseases. In this section, we review two nano‐encapsulation technologies used for probiotic cells in the treatment of CRC or colitis: single‐cell encapsulation and nanofibers encapsulation. We further analyze the distinctive properties of these various nano‐encapsulation methods.

#### 4.2.1 Single‐Cell Encapsulation Systems

Single‐cell encapsulation is based on the formation of nanofilms around individual probiotic cells, enabling their direct delivery to the colon without needing release from an encapsulation matrix. These nanocoatings approach provides enhanced protection for probiotics in the harsh gastrointestinal environment and promotes stronger mucus adhesion, thereby reducing the risk of bacterial translocation.^[^ ^169^ ^]^ Moreover, specific nanocoatings confer additional therapeutic functions on probiotics, such as immunomodulation, antioxidant activity, and anti‐inflammatory properties, which enhance their efficacy in treating CRC or colitis and improve safety. Encapsulated probiotics have also shown promise in preventing and treating diseases at a cellular level.^[^ ^170^ ^]^ An assortment of nanoscale coatings has been developed for the encapsulation of probiotics, encompassing polysaccharides, lipid membranes, proteins, cell membranes, and modified polymers. Based on the different modes of interactions in the encapsulation process, we categorize these methods into non‐covalent adsorption, covalent conjugation, and substrate encapsulation. In this section, we present an overview of the contemporary single‐cell encapsulation methods used for probiotic cells in the treatment of CRC or colitis.

##### Non‐Covalent Adsorption

**Figure 8:**
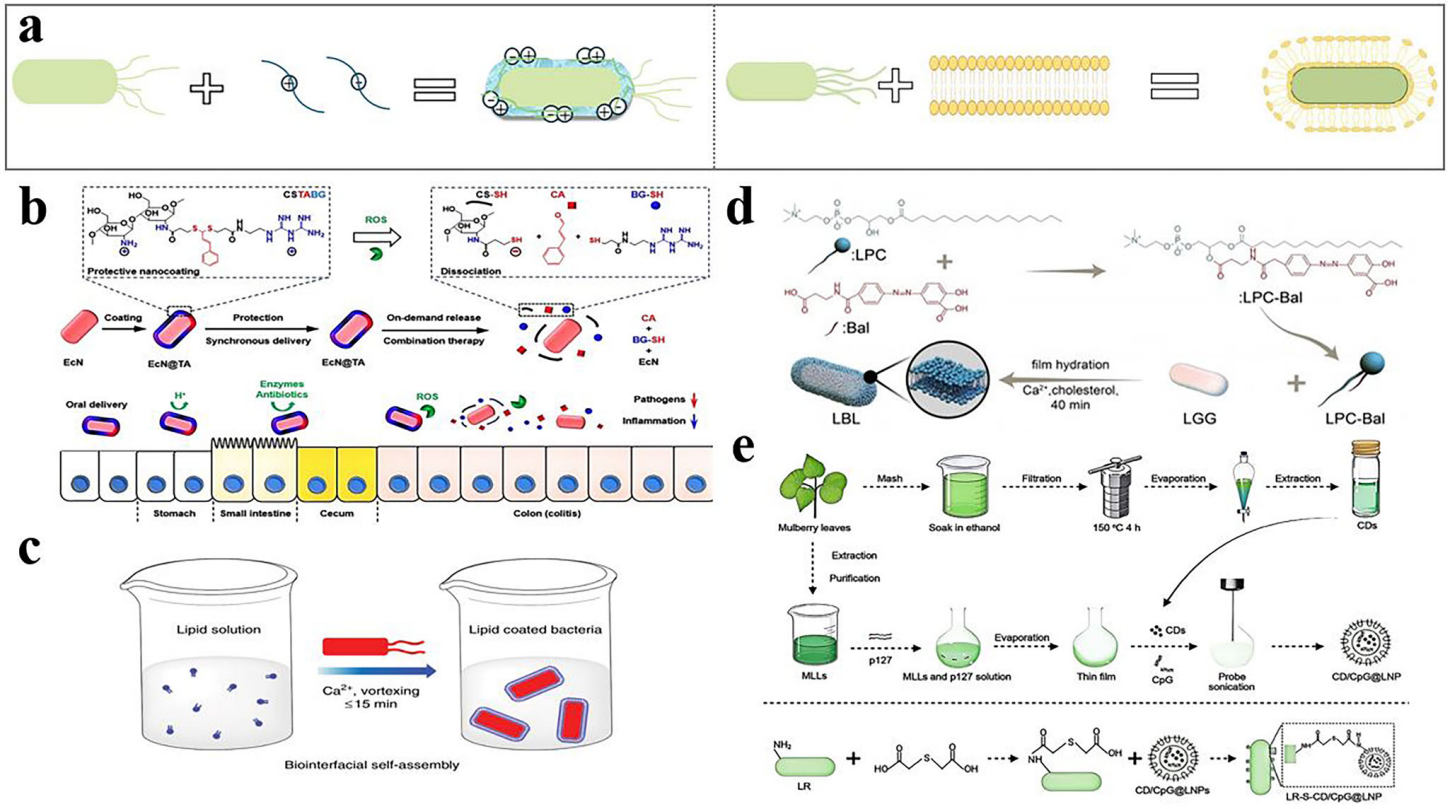
Single‐cell encapsulation of probiotics by electrostatic deposition and lipid‐bilayer coatings. a) Schematic illustration of electrostatic deposition and lipid‐bilayer coating adsorption methods. b) Schematic diagram of the utilization of a triggerable prodrug nanocoating to enable lesion‐targeted dual activation of living microbial therapeutics (LMTs) and small‐molecular drugs (SMDs) for combination therapy. Reproduced with permission.^[^ ^172^ ^]^ Copyright 2023, American Chemical Society. c) Schematic illustration of the preparation of lipid membrane coated bacteria by biointerfacial supramolecular self‐assembly.^[^ ^140^ ^]^ d) Balsalazide (Bal) was conjugated to 1‐palmitoyl‐sn‐glycero‐3‐phosphocholine (LPC‐Bal) and modified onto the surface of Lacticaseibacillus rhamnosus GG (LGG) via the interfacial supra‐molecular self‐assembly.^[^ ^179^ ^]^ e) Preparation and physicochemical characterization of LR‐S‐CD/CpG@LNPs.^[^ ^180^ ^]^

The layer‐by‐layer (LbL) deposition method typically entails the sequential adsorption of positively or negatively charged polymers onto substrates with opposite charges to form multiple polymer layers (**Figure** **8**a). Cationic polymers, such as CS, can be directly deposited onto the anionic surfaces of bacteria, like alginate, through electrostatic attraction.^[^ ^171^ ^]^ For instance, Kuang et al. selected CS as a carrier to conjugate biguanide (BG), leveraging its positive charge to facilitate deposition onto the negatively charged surface of *EcN* through electrostatic interactions (Figure 8b). They employed oxidation‐responsive aromatic thioacetal (TA) as a linker to create a CS‐BG prodrug (CS‐TA‐BG) that forms a protective nanocoating on *EcN*. Upon oral administration, this nanocoating effectively shields *EcN* from gastrointestinal insults, while its stability allows for the simultaneous and spatially aligned co‐delivery of *EcN* and BG. At the lesion site, elevated ROS levels trigger TA linker cleavage, resulting in dual release of the conjugated drug and the linker‐derived cinnamaldehyde (CA), while charge reversal releases and activates *EcN* for proliferation. The combined delivery of *EcN* and BG with the linker‐derived antibacterial CA to colitic lesions demonstrated significant synergistic therapeutic efficacy in a murine model of pathogen‐induced colitis.^[^ ^172^ ^]^ *Bacillus amyloliquefaciens* (*B. amyloliquefaciens*) was loaded into CS and sodium alginate nanoparticles in another study. The results indicated that these nanoparticles loaded with *B. amyloliquefaciens* were more resilient in simulated gastrointestinal conditions than the unencapsulated bacteria Specifically, the load of unencapsulated *B. amyloliquefaciens* dropped from 8.9 ± 0.23× 10^9^ at 30 min after incubation to 8.1 ± 0.21 × 10^5^ after 120 min. In contrast, the load of *B. amyloliquefaciens* in nanoparticles decreased from 9.3 ± 0.19 × 10^9^ at 30 min to 6.1 ± 0.20 × 10^8^ after 120 min. Furthermore, it was shown that BANPs could upregulate Bcl‐2 and Bax expression and downregulate cytochrome c and caspase‐3, demonstrating their role in colonic apoptosis attenuation. They also prominently reduced the overexpression of inflammatory markers such as IL‐6, IL‐1β, TNF‐α, COX‐2, and iNOS, highlighting their potential therapeutic efficacy in IBD treatment.^[^ ^173^ ^]^ Similarly, Deng et al. employ hyaluronic acid (HA)‐nanocoated *C. butyricum* to repair damaged gut tissue and reduce inflammation. It can be coated with anionic HA by inverting the surface charge of *C. butyricum* with cationic CS via electrostatic interaction under cytocompatible conditions. This HA coating protected *C. butyricum* from GIT challenges and enhanced its aggregation at inflamed intestinal sites. The combination of HA's immunosuppressive properties and *C. butyricum*’s butyrate production effectively relieved intestinal mucosal damage.^[^ ^174^ ^]^ Coatings formed through electrostatic interactions can be unstable in varying pH or ionic strength conditions, which may expose the encapsulated probiotics to the external environment.^[^ ^175^ ^]^ Moreover, the elevated charge density within the microenvironment encircling the cell has the propensity to diminish cellular activity and may potentially compromise cellular integrity.^[^ ^176^ ^]^ As such, there is a growing tendency to resort to alternative non‐covalent interactions for coating assembly.

Research on encapsulating probiotics using lipid nanoparticles, rather than polymers, has seen a rise in recent years. Liposomes, predominantly composed of phospholipids and cholesterol, have been meticulously synthesized using substances such as cholesterol, lecithin, and vitamin E. The preparation of liposomes was accomplished through the membrane dispersion technique, culminating in the procurement of a single‐cell “nanoarmor” (Liposome) encapsulating probiotics via the admixture of the liposome solution with bacterial sediment.^[^ ^143b^ ^]^ Phospholipids self‐assemble into a phospholipid bilayer structure on the surface of probiotics to protect probiotics from strong acids and digestive enzymes, while cholesterol improves flexibility and permeability.^[^ ^177^ ^]^ Cao et al. coated *EcN* with a lipid membrane, demonstrating that the encapsulated *EcN*s had nearly three times the survival rate in the mouse stomach and over four times the bioavailability in the gut compared to uncoated bacteria.^[^ ^140^ ^]^ (Figure 8c) The prolonged residence in the gut can last up to 4 days after administration. They also demonstrated that coated *EcN*s achieved significantly increased efficacies in DSS‐induced colitis mouse models. Similarly, Xu et al. found that *L. rhamnosus* and *Bifidobacterium longum subsp. Longum* encapsulated in single‐cell “nanoarmor” (Liposome) significantly improved probiotics' colonization ability and viability.^[^ ^178^ ^]^ Following a 48‐h exposure within the gastrointestinal tract, the probiotics formulation demonstrated a superior fluorescence intensity relative to the naked bacteria group, suggesting that the lipid membrane is instrumental in extending the retention time of bacteria within the gastrointestinal lumen. Moreover, the probiotics formulation was observed to modulate the colonic microenvironment, leading to an increase in the population of goblet cells and the expression of protective proteins, including claudin and occludin. Therefore, the combined administration of nanovaccines and probiotics formulation could bolster resistance against AOM/DSS‐induced CRC by preserving the structural integrity of the epithelial tissue. Balsalazide (Bal), a prodrug of 5‐ASA, was conjugated to 1‐palmitoyl‐sn‐glycero‐3‐phosphocholine to form LPC‐Bal (Figure 8d). This conjugate was successfully modified onto the surface of *L. rhamnosus* GG via interfacial supramolecular self‐assembly, creating a drug‐loaded lipid coating. Upon reaching the colon, the coated probiotic released 5‐ASA under the action of azoreductase, which effectively regulated intestinal inflammation and provided a favorable microenvironment for LGG colonization. Concurrently, the coated probiotic modulated the gut microbiota and improved epithelial barrier function, thereby synergistically ameliorating UC.^[^ ^179^ ^]^ In another study, Xu et al. synthesized carbon dots (CDs) and CpG‐encapsulated mulberry leaf lipid (MLL) nanoparticles (CD/CpG@LNPs) using a thin film hydration technique (Figure 8e). These nanoparticles were subsequently functionalized to the surface of *Limosilactobacillus reuteri* (LR) via ROS‐responsive linkers, creating an engineered bacterium (LR‐S‐CD/CpG@LNP). This nanomedicine‐engineered bacterium exhibited optical responsiveness, immune‐stimulating activity, and the ability to regulate microbiota metabolome. Mechanistically, the intrinsic photothermal and photon‐induced cytotoxic effects of CD/CpG@LNPs induced the generation of cytotoxic ROS and immunogenic apoptosis within colorectal tumor cells. The resultant neoantigens, combined with the emancipated CpG, formed a potent in situ immunogen that facilitate the maturation of immature dendritic cells served as a robust in situ immunogen that facilitates immature dendritic cells' maturation. Furthermore, the mature dendritic cells and metabolites secreted by LR promoted the infiltration of cytotoxic T lymphocytes into the tumor microenvironment, aiding in the excision of colorectal malignancies.^[^ ^180^ ^]^

##### Substrate Encapsulation

**Figure 9:**
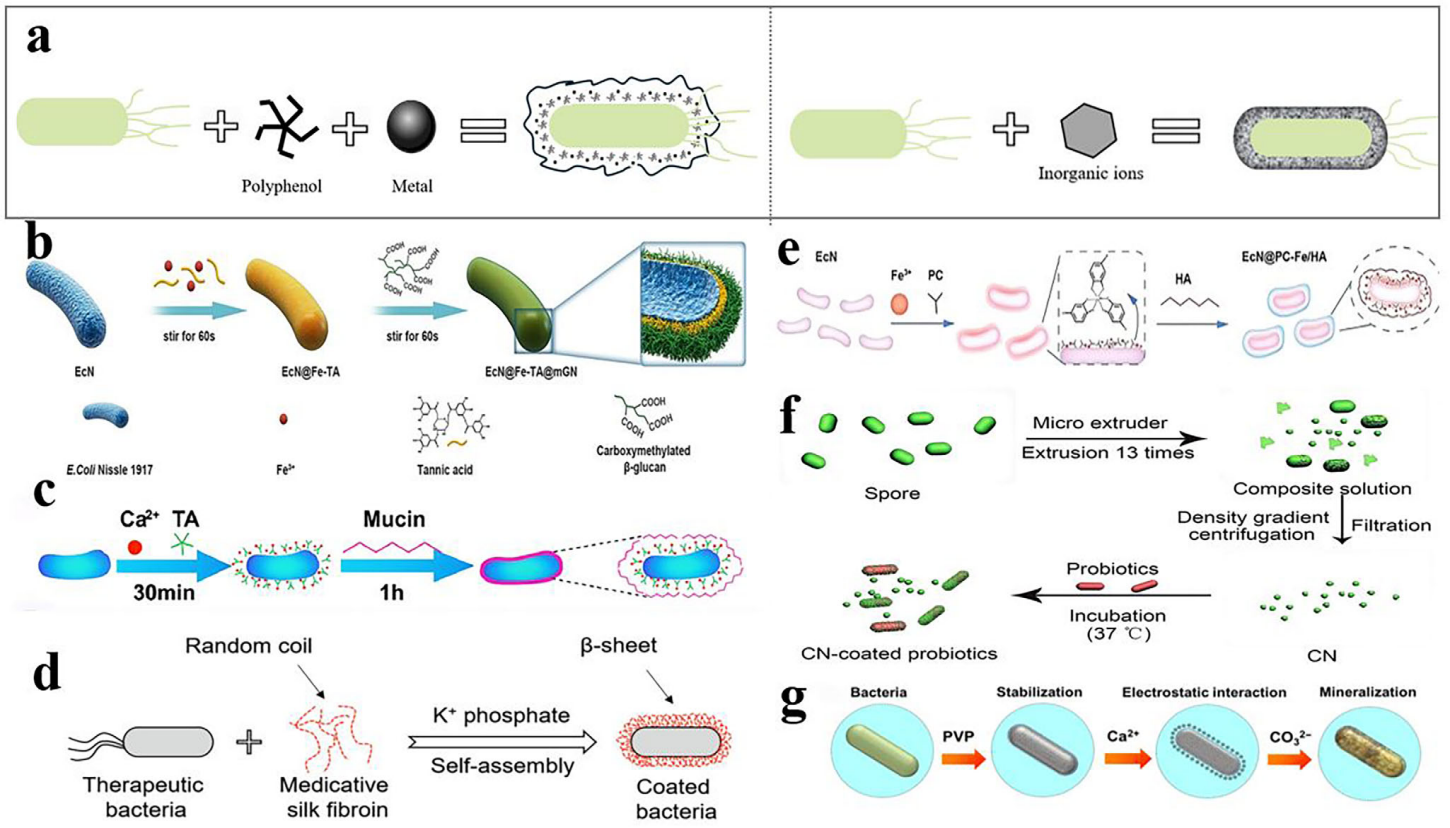
Single‐cell encapsulation of probiotics by ligand complex and biomineralization encapsulation method. a) Schematic illustration of ligand complex and biomineralization encapsulation method. b) Schematic illustration of the fabrication of *EcN*@Fe‐TA (Tannic acids) @β‐glucan (mGN). Reproduced with permission.^[^ ^182^ ^]^ Copyright 2023, American Chemical Society. c) Decorating the therapeutic bacteria with TA and mucin by layer‐by‐layer coating technology. Reproduced with permission.^[^ ^183^ ^]^ Copyright 2022, American Chemical Society. d) Coating therapeutic bacteria with medicative silk fibroin by biointerfacial self‐assembly. Reproduced with permission.^[^ ^191^ ^]^ Copyright 2021, Wiley‐VCH GmbH. e) Demonstration of a synthetic procedure for *EcN*@procyanidine (PC)‐Fe/hyaluronic acid (HA) with PC, FeIII, and high‐molecular‐weight hyaluronan (HMW‐HA) by layer‐by‐layer coating strategy. Reproduced with permission.^[^ ^185^ ^]^ Copyright 2024, Wiley‐VCH GmbH. f) Schematic of process of spore‐coated nanomaterial (CN) preparation from probiotics spores and the mechanism of CN‐coated probiotics for colitis treatment. Reproduced with permission.^[^ ^189^ ^]^ Copyright 2021, Wiley‐VCH GmbH. g) Schematic illustration of biointerface mineralization that generates ultraresistant gut microbes as oral biotherapeutics.^[^ ^194^ ^]^

Metal‐phenolic networks (MPNs) are formed by cross‐linking polyphenols with metal ligands (**Figure** **9**a). The as‐synthesized MPN complexes feature pH responsiveness, controllable size and rigidity, adhesion, self‐repair, protection, and toughness.^[^ ^181^ ^]^ Therefore, it is widely used in biological applications. However, the metal‐polyphenol network disintegrates under acidic conditions, making them inappropriate for use as a protective barrier for probiotics. Instead, MPNs serve more effectively as an adhesive layer, bridging probiotics with protective wall materials and enhancing their retention and delivery within the GIT. Integrating metal‐polyphenol networks with other technological advancements can revolutionize the development of delivery systems with optimal protective and adhesive properties. For instance, Xie et al. equipped EcN with a Fe^3+^‐tannic acid cross‐linked network and carboxymethylated β‐glucan (GN) to create EcN@Fe‐TA@mGN (Figure 9b). Shielded by the Fe‐TA@mGN “armor”, the viability of the modified EcN was enhanced approximately 1720‐fold compared to the unmodified EcN following exposure to SGF. Moreover, the retention rate of EcN@Fe‐TA@mGN in the intestines reached 47.54 ± 6.06% after 16 h of administration, whereas the majority of the unmodified EcNs were eliminated within 8 h post‐administration. Given that GN possesses multiple bioactivities (e.g., modulating gut microbiota and repairing intestinal barriers), combined with its upper gastrointestinal stability and specific recognition by Dectin‐1 on M cells, this system achieves synergistic therapeutic effects for improved colitis management. ^[^ ^182^ ^]^

Since probiotics colonize and grow within the mucus layer, Shi and colleagues modified probiotics with mucin and Tannins/Tannic acids (TA) to spontaneously regulate the pathological microenvironment of inflammatory diseases (Figure 9c). These mucin‐fortified probiotics demonstrated enhanced resistance to rigorous gastrointestinal conditions and improved intestinal mucosa adhesion, facilitating stable colonization and growth within the mucus layer while maintaining their protective coating. Moreover, *EcN*@TA‐Ca^2+^@Mucin can significantly mitigate inflammation through the scavenging of ROS scavenging and minimize the adverse effects of bacterial translocation associated with IBD, thereby increasing the abundance and diversity of the gut microbiota.^[^ ^183^ ^]^ Building on this foundation, researchers have explored other methods to enhance the resilience and efficacy of probiotics. In another study, procyanidine (PC) coordinated with Fe (III) to form MPNs, which were then incorporated into a high‐molecular‐weight hyaluronan (HMW‐HA) framework (Figure 9e). Coated probiotics exhibited superior resistance to the harsh environment, and after 1–2 h exposure to SGF, the survival rate of *EcN*@PC–Fe/HA was approximately six times higher than that of naked *EcN*. Previous studies indicated that key functional groups (carboxyl, hydroxyl, and acetylamino) of HMW‐HA interact strongly with mucin via multiple molecular interactions (hydrogen bonding, hydrophobic interactions, and electrostatic forces), thereby significantly prolonging intestinal residence time of coated bacteria.^[^ ^184^ ^]^ The luminescence intensity of the unadorned *EcN*‐mcherry diminished precipitously 24 h subsequent to oral administration. Conversely, the fluorescence emitted by *EcN*mCherry@PC‐Fe and *EcN*‐mCherry@PC‐Fe/HA complexes remained discernible up to 120 h post‐administration. Additionally, *EcN*@PC‐Fe/HA probiotics demonstrated a marked preference for adhering to the inflamed areas within the gastrointestinal tract, attributed to the interaction between HA and the overexpressed CD44 receptors. This specific binding enhanced the targeting at the disease site and the bioavailability, culminating in an improved therapeutic outcome.^[^ ^185^ ^]^ This approach of enhancing probiotic performance through nanocoatings is not limited to the examples mentioned above. Although the use of polyphenol‐based nanocoatings for probiotics is a relatively recent development, it has already shown promise due to its ability to improve the viability of probiotics, enhance mucosal adhesion, and provide an adjuvant treatment effect for diseases. As a result, nanocoating probiotics have the potential to become a hot research direction in the field of probiotic encapsulation.

Regarding to targeted delivery systems, recent advances tend to focus on single‐cell encapsulation platforms conjugated with specific targeting moieties (e.g., antibodies or ligands) for precise antigen recognition. For example, building on the specificity of HA binding to CD44 receptors, Zhu et al. meticulously engineered a hyaluronic acid‐inulin (HA‐IN) coated Enterococcus faecium (E. faecium, EF47) for the purpose of achieving colon‐specific delivery, with the intent to combat the pathogen Fusobacterium nucleatum.^[^ ^186^ ^]^ The intrinsically protected EF47 was resilient against the rigorous conditions of the gastrointestinal tract, particularly in the colon where it was selectively degraded, thereby functioning as a prebiotic to enhance the proliferation of EF47. Subsequent validation demonstrated that this system significantly augmented tumor tissue adhesive properties, effectively counteracted Fusobacterium nucleatum's proliferation, and exerted a notable antitumor effect. Therefore, this colon‐targeted delivery system offered a novel platform for realizing high‐activity and adhesive delivery of probiotics, potentially improving the therapeutic efficiency of CRC. Although targeted probiotic delivery systems based on specific recognition of disease biomarkers or signaling molecules remain in the early stages of development, they represent a promising approach for precision microbiome therapeutics. Further endeavors are required in this emerging field.

In recent years, spores, a natural material, have been utilized as coatings to enhance probiotic performance. Spores are characterized by their robust outer layer composed of hydrophobic proteins, which protect probiotics against the stringent gastrointestinal environment and extreme temperature variations, enabling successful traversal of the GIT and colonization in the large intestine.^[^ ^187^ ^]^ The surface proteins of spores exhibit significant resistance to external attacks, making them suitable candidates for chemical modification into novel carrier materials.^[^ ^188^ ^]^ Notably, Song et al. innovatively developed a multifunctional spore coat nanomaterial designated as CN, which is affixed to probiotic surfaces, forming CN‐coated probiotics (Figure 9f). This method involves mechanically extruding spore coatings while retaining their high resistance and natural affinity properties, facilitating the exponential growth and competitive establishment of probiotics. CN demonstrates pronounced anti‐inflammatory activity and enhances probiotic efficacy across various functions, including microbiota regulation, gut barrier maintenance, and tumor prevention.^[^ ^189^ ^]^ Moreover, spores are widely utilized as oral delivery carriers due to their ability to resist stomach acidity and facilitate the germination of probiotics upon exposure to intestinal nutrients. This capability enables colonization in the intestine. Compared to alternative systems, *Bacillus* spores offer distinct advantages such as prebiotic function, oral safety profile, cost‐effectiveness, ease of synthesis, substantial drug loading capacity, biocompatibility, and targeted colon delivery.^[^ ^187^ ^]^ Inspired by these physiological characteristics, Song and colleagues modified *Bacillus cagulans* spores by incorporating deoxycholic acid (DA). Subsequently, chemotherapeutic drugs (doxorubicin and sorafenib, DOX/SOR) were encapsulated within these spores to form a self‐sustaining nanocarrier platform. These nanoparticles protected the drug cargo as they navigated through the harsh gastric environment and were transported to the intestine for swift colonization. Additionally, these nanoparticles efficiently enter epithelial cells via the apical sodium‐dependent bile acid transporter (ASBT)‐mediated pathway, which helped overcome intestinal epithelial barriers and enhanced drug release on the basolateral side. This therapeutic approach has shown promising results in reducing inflammation, suppressing tumors, restoring intestinal barrier function, and maintaining gut microbiota equilibrium.^[^ ^190^ ^]^ In parallel, Yin et al. developed an oral delivery system, named SPORE‐CUR‐FA, based on probiotic *Bacillus* spores for curcumin delivery in CRC therapy.^[^ ^187^ ^]^ In this system, curcumin and folate are chemically attached to the spore surface without affecting the spores’ activity. In vivo studies have demonstrated that it significantly increased curcumin's oral bioavailability, prolonged its retention period in the body, and exhibited potent anti‐colon cancer properties. These studies collectively highlight the potential of spores as carriers for enhancing probiotic efficacy and delivering therapeutic agents, offering innovative avenues for treating various diseases.

In addition, coated probiotics with therapeutic nanocoating is also a protective strategy, which can provide exogenous functions (e.g., anti‐inflammatory properties) and synergistically enhance biotherapy. Silk fibroin was employed to self‐assemble on the surface of bacteria, transitioning from a random coil to a β‐sheet conformation and then forming a complete and stable nanocoating (Figure 9d). Inspired by the excellent properties of silk fibroin in biological structures, numerous studies have encapsulated cells using this material. Hou et al. meticulously crafted a silk fibroin coating for the encapsulation of EcN. Following a 3‐h exposure to simulated gastric juice, the number of viable encapsulated EcN was found to be 52‐fold higher compared to the non‐encapsulated EcN. The nano‐scale silk fibroin coating served not only as an effective barrier to protect against gastric degradation but also mitigated inflammation and exerted a synergistic enhancement on the therapeutic efficacy of probiotics in the treatment of intestinal mucositis.^[^ ^191^ ^]^ Similarly, Yang et al. found that silk fibroin nanocoating significantly improved the survival of coated bacteria against gastric insults.^[^ ^192^ ^]^ It can also modulate the balance of microbiota and metabolites in the gut by increasing their richness and diversity, restore the intestinal barrier integrity, and consequently exhibit stronger anti‐inflammatory efficacy than the traditional immunosuppressant widely used in the clinic (5‐ASA), without side effects and resulting in effective treatment of acute colitis.

Biomineralization is another substrate encapsulation method that can produce a hard coating around soft tissues, thereby enhancing the survival of organisms.^[^ ^193^ ^]^ (Figure 9a) Inspired by this natural phenomenon, researchers applied it to coat probiotics. CaCO_3_‐coated *B. fragilis* (BF839) was meticulously prepared through an electrostatic interaction‐facilitated interfacial mineralization process, wherein polyvinylpyrrolidone was employed as a stabilizing reagent to ensure colloidal stability (Figure 9g). Following oral ingestion, the outer casing effectively engages in neutralizing gastric acid, concurrently liberating the entrapped bacteria via a prompt and autonomous biphasic decomposition process. Apart from neutralizing acidic content, the resultant calcium ions facilitate the aggregation of biliary acids at the micellar level, thereby endowing the formulation with a dual protective mechanism against the deleterious effects of both gastric and biliary acids, thus guaranteeing the integrity and viability of the encapsulated bacteria. The probiotic strain *B. fragilis*, fortified with a mineral coating, exhibited significant therapeutic benefits in a DSS‐induced murine colitis model.^[^ ^194^ ^]^

##### Covalent Conjugation

**Figure 10:**
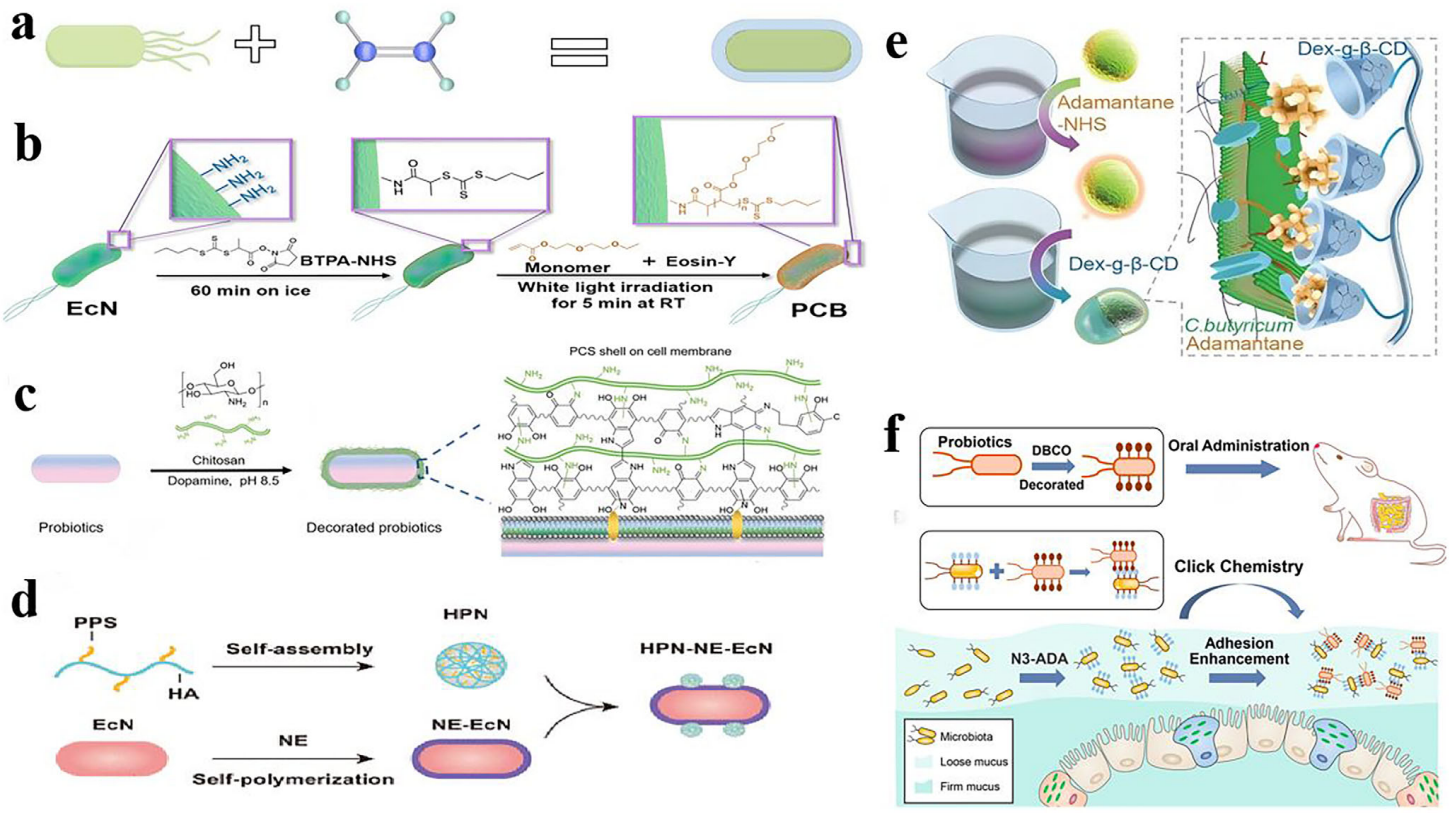
Single‐cell encapsulation of probiotics by covalent binding methods. a) Schematic illustration of covalent binding encapsulation. b) The principle of surface‐initiated polymerization on *EcN* to produce polymer‐coated bacteria (PCB). Reproduced with permission.^[^ ^9^ ^]^ Copyright 2022, American Chemical Society. c) Decoration of probiotics by wrapping with a multimodal polydopamine (PDA) coating and the chemical structure of chitosan co‐deposited coating on cell surface. Reproduced with permission.^[^ ^196^ ^]^ Copyright 2021, Wiley‐VCH GmbH. d) Preparation of HPN by self‐assembly of HA‐PPS molecule, encapsulation of *EcN* with the norepinephrine (NE) layer, and conjugation of HPN to the surface of *EcN*.^[^ ^197^ ^]^ e) Schematic illustration for the combination of probiotics and prebiotics through host‐guest chemistry. Reproduced with permission.^[^ ^141^ ^]^ Copyright 2020, Wiley‐VCH GmbH. f) Schematic illustration of bioorthogonal‐mediated bacterial delivery to enhance probiotics colonization in the gut.^[^ ^200^ ^]^

Conjugating substances can form coatings around individual probiotics to specific chemical groups on the cell surface (**Figure** **10**a). A successful example of covalent binding is the conjugation of the chain‐transfer agent (CTA) BTPA‐NHS to amino groups on the *EcN* cellular surface (Figure 10b). Polyethylene glycol (PEG) polymer brushes were synthesized from CTA to produce the polymer‐coated bacteria (PCB), which can promote the tumor colonization ability of *EcN* by prolonging the blood reservation with a lower inflammatory reaction. Notably, the polymer coating did not alter the cellular viability or dimensions of the bacteria. More importantly, PCB has the capability to transform the prodrug 5‐fluorocytosine (5‐FC) into 5‐FU, which is utilized for the treatment of tumors. The combination of PCB with 5‐FC resulted in a tumor growth inhibition rate of 56.1 ± 11.1%, markedly higher than that of the *EcN* with 5‐FC treatment (19.0 ± 9.9%).^[^ ^9^ ^]^ Another exemplar instance of covalent binding involves the conjugation of polydopamine (PDA) to amine and/or sulfhydryl moieties on the surface glycoproteins of bacterial cells.^[^ ^195^ ^]^ Inspired by an adhesion protein in mussels, PDA features catechol groups that enable strong surface adhesion. Using a one‐step oxidation and self‐polymerization method, dopamine was successfully deposited on the surface of the probiotic *EcN*.^[^ ^196^ ^]^ This PDA coating can then be functionalized through various chemical reactions, including hydrogen bonding, π–π stacking, Michael addition, or Schiff base reactions. A case in point is the integration of CS into the PDA coating through Michael's addition and Schiff base reactions, culminating in the creation of *EcN* encapsulated with a PDA‐CS composite layer (Figure 10c). The dual cytoprotective and targeting effects observed in this study significantly enhanced the oral bioavailability and tissue‐specific accumulation, as demonstrated by experimental results.^[^ ^196^ ^]^ Additionally, Liu et al. synthesized a hyaluronic acid‐poly (propylene sulfide) (HA‐PPS) conjugate that self‐assembled into nanoparticles (HPNs) due to the amphiphilic nature of the HA‐PPS combination (Figure 10d). These ROS‐scavenging HPNs were used to encapsulate *EcN* cells with an outer polynorepinephrine (NE) coating. The NE layer protected *EcN* from oxidative stress, enhancing both bacterial viability and intestinal retention through strong mucoadhesion. In DSS‐induced murine colitis models, the HPN‐NE‐*EcN* system showed significantly improved prophylactic and therapeutic efficacy, while also promoting gut microbiota diversity. ^[^ ^197^ ^]^

TAs possess polyphenolic groups that can easily bind to bacterial surface proteins, allowing for the attachment of additional substances.^[^ ^141^ ^]^ For example, poloxamer 188 (F68) has been shown to self‐assemble on TA‐coated bacterial surfaces, creating a coating (TA@F68) that enhances probiotic survival and strengthens the intestine colonization by autonomously modulating the pathological microenvironment. In DSS colitis mice, this coating demonstrated excellent anti‐inflammatory effects and a strong ability to restore intestinal barrier functions and prompt the balance of gut microbiota.^[^ ^198^ ^]^ Liu et al. delineated a sophisticated double‐layer encapsulation methodology utilizing TA in conjunction with enteric polymer L100 to encapsulate *EcN*. This novel strategy displayed exceptional resilience in withstanding the rigorous conditions prevalent within the gastrointestinal tract.^[^ ^199^ ^]^ Furthermore, the pH‐sensitive disintegration of the outer L100 coating facilitates targeted delivery of the TA‐*EcN* complex specifically to the intestinal region. The intrinsic mucoadhesive properties of the TA layer significantly enhance the retention period of *EcN* within the intestine, while simultaneously preserving its viability and promoting its proliferation. This mechanism significantly contributed to the enhanced prophylactic and therapeutic effectiveness against colitis.

Bio‐coupling chemistry has been investigated for its potential to enhance the adhesion of probiotics to epithelium cells, thereby improving probiotic delivery and colonization. Metabolic amino acid engineering was utilized to facilitate the metabolic integration of azido‐functionalized D‐alanine into the peptidoglycan structure of gut microbiota, thereby permitting in situ bioorthogonal conjugation with probiotics that have been modified with dibenzocyclooctyne (DBCO). As illustrated in Figure 10f, this approach significantly enhanced bacterial adherence within intricate physiological milieus. Notably, the DBCO‐modified strain of *C. butyricum* exhibited enhanced retention within the gut lumen and markedly ameliorated disease symptoms in the DSS‐induced colitis model.^[^ ^200^ ^]^ Similarly, Xie et al. attached doxorubicin to *EcN* using acid‐labile cisaconitic anhydride linkers to achieve tumor‐specific targeting and drug release in response to the tumor microenvironment.^[^ ^201^ ^]^ The treatment with *EcN* conjugated to doxorubicin through these acid‐labile linkers has been found to be more effective in inhibiting tumor growth, extending survival in animal models, and inducing apoptosis in cancer cells compared to free doxorubicin or doxorubicin conjugated to *EcN* with stable succinic anhydride linkers.

Zheng and colleagues developed a novel probiotic delivery system by combining *C. butyricum* with a chemically modified prebiotic dextran to create prebiotic‐encapsulated probiotic spores, denoted as spores‐dex (Figure 10e). The suggestion of incorporating prebiotics, in conjunction with the co‐encapsulation technique of probiotics and prebiotics, represents a prospective strategy for augmenting the viability and functional efficacy of probiotics.^[^ ^202^ ^]^ For example, dextran enhances intestinal mucosal adherence and, subsequent to the fermentation by *C. butyricum*, produced abundant SCFAs, thereby exerting a notable anti‐tumorigenic influence. In addition, oral prebiotic intake is advantageous for human health, as it enhances mineral absorption, modulates the immune system, and promotes metabolic processes.^[^ ^162^, ^203^ ^]^ It has been found that following oral ingestion, spores‐dex selectively accumulates within the colon, thereby enhancing the prevalence of SCFA‐producing bacteria (e.g., *Eubacterium* and *Roseburia*), and markedly elevating the overall biodiversity of the microbiota.^[^ ^141^ ^]^ Moreover, co‐delivery of probiotics and prebiotics is a frequently employed strategy to augment the therapeutic efficacy of probiotics in the context of oral delivery. For example, Subirade and colleagues elucidated that the viability of encapsulated probiotics exposed to acidic environments in vitro was markedly enhanced by incorporating inulin as a co‐encapsulating agent.^[^ ^204^ ^]^

#### 4.2.2 Nanofibers Encapsulation Systems

Recent studies have significantly increased the number of probiotics encapsulated within nanofibers, leveraging coaxial electrospinning technology to enhance surface area and porosity, thereby protecting bioactivity and facilitating the release of bioactive substances. A pivotal benefit of electrospinning technology lies in its ability to avoid heat generation during the encapsulation phase, obviate the requirement for deleterious organic solvents, and maintain the integrity of bioactive compounds.^[^ ^143b^ ^]^ Hitherto, an array of materials, encompassing polysaccharides, proteins, and lipids, have been utilized as encapsulating agents within the electrospinning framework.^[^ ^205^ ^]^

**Figure 11:**
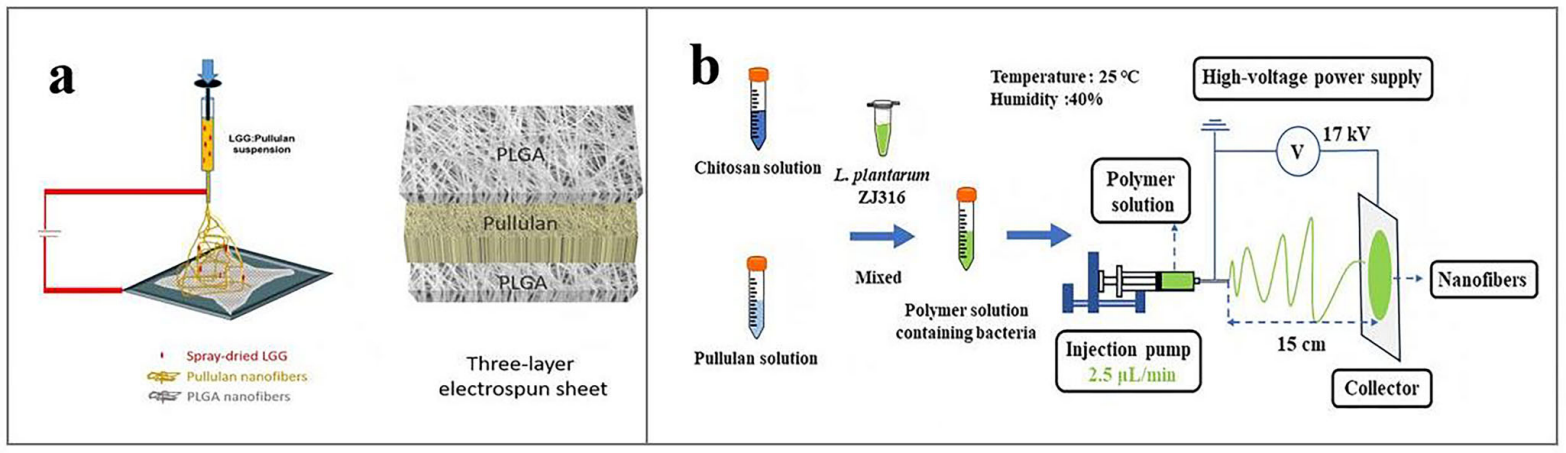
Nanofibers encapsulation of probiotics. a) Multilayered fibrous structure of Poly‐lactic‐co‐glycolic acid (PLGA)‐Pull‐PLGA. Schematic representation of the layer‐by‐layer electrospinning (PLGA, LGG:Pull) is applied to fabricate a three‐layer electrospun construct where the pullulan layer is sandwiched by two PLGA layers. Reproduced with permission.^[^ ^207^ ^]^ Copyright 2021, Elsevier Ltd. b) Schematic diagram of electrospinning. Reproduced with permission.^[^ ^208^ ^]^ Copyright 2024, Elsevier B.V.

LGG bacteria were encapsulated within pullulan‐based nanofibers, which were subsequently enveloped by bilayered layers of electrospun Poly‐lactic‐co‐glycolic acid (PLGA). PLGA is widely recognized for its utility as a protective carrier for bioactive compounds, safeguarding them from detrimental conditions and enhancing their oral or systemic delivery and absorption.^[^ ^206^ ^]^ (**Figure** **11a**) According to an in vitro study, the viability of LGG encapsulated in a single pullulan‐based layer was much lower (4 × 10^6^ CFU·g^−1^) than that in the triple‐layered structure, which boasted a significantly higher count of 2.4 × 10^9^ CFU·g^−1^.^[^ ^207^ ^]^ Zhang et al. also prepared encapsulated nanofibers of *L. fermentum* ZJ316 using pullulan and CS by electrospinning.^[^ ^208^ ^]^ The encapsulation of ZJ316 within the nanofibrous matrix markedly augmented its viability in simulated gastrointestinal environments, as evidenced by the significantly elevated survival rates of 87.24% in gastric fluid and 79.71% in intestinal fluid, respectively, when compared to the viability of unencapsulated bacteria (Figure 11b).

Prebiotics are endowed with the capacity to augment the bio‐metabolic processes, viability, and proliferative potential of probiotics, and further boost their survival rate as they pass through the upper gastrointestinal tract, amplifying their beneficial effects in the large intestine. Consequently, the co‐administration of probiotics and prebiotics is a widely adopted strategy to improve the oral bioavailability of probiotics. For example, Yu et al. encapsulated *L. plantarum* in polylactic acid (PLA) nanofibers using coaxial electrospinning.^[^ ^209^ ^]^ PLA is biocompatible, degradable in the human body and has good acid resistance. In vitro digestion simulations showed that the survival rate of probiotics in these coaxial electrospun nanofibers exceeded 72%. Fructooligosaccharides (FOS) incorporated in shell electro‐spun nanofibers could selectively promote the further growth of lactic acid bacteria. A similar study reported a significant increase in the viability of *L. plantarum* by 1.1 log when 2.5% (w/w) of FOS was used in the electrospinning process.^[^ ^210^ ^]^ And Wen et al. reported that a quercetin‐loaded electro‐spun fiber mat (Q‐loaded EFM) exhibited good prebiotic effects owing to the addition of prebiotic galactooligosaccharide (GOS). It promoted sustained and targeted colon‐specific quercetin (Q) release and enhanced its release rate. Furthermore, it showed anticancer activity by halting the cell cycle at the G0/G1 phase and inducing apoptosis in colon cancer cells.^[^ ^211^ ^]^ These results suggested that electrospun fiber‐based encapsulation systems that protect gastric‐sensitive next‐generation probiotics from acidic degradation, combined with prebiotic–probiotic synergism to enhance antitumor immunity, may represent a promising strategy for clinical translation.

## 5 Challenges, Limitations, and Prospects

### 5.1 Challenges to the Development of Probiotics Oral Delivery Systems

1. Precision of targeted delivery: Probiotic delivery systems are comprised of a variety of materials, which are integrated via covalent or non‐covalent bonds. Depending on the strength of these bonds, an excessively stable delivery system may preclude the timely release of probiotics at target sites. Conversely, an overly unstable system may result in the premature discharge of the encapsulated probiotics prior to reaching their intended destination. In both cases, it is difficult to ensure that probiotics accurately reach the site of inflammation or tumor.^[^ ^212^ ^]^ Some synthetic components, like surfactants and polymers used in oral systems, may pose toxicity risks, potentially disrupting the microbiome or causing gastrointestinal inflammation. Therefore, it is essential to develop materials that integrate targeting to pathological sites or possess responsiveness, such as ROS responsiveness or the ability to bind to CD44 receptors, while also ensuring they are biocompatible, biodegradable, protective, and non‐toxic to the host. The specific strategies will be discussed in Section 5.3.
2. Complex preparation process and difficulty in ensuring consistency: Preparing probiotic delivery systems involves intricate steps: probiotic cultivation, material preparation, carrier construction, and drug loading. Since laboratory research often relies on manual operations, achieving high automation and standardization is difficult, leading to significant differences in each preparation process. Errors in manual operations may affect the activity of probiotics, drug loading efficiency, and the overall performance of the delivery system, thus failing to ensure consistency in quality and function for each production. This poses a major obstacle to the high repeatability and controllability required in clinical applications for CRC.
3. Insufficient stability, long‐term storage, and liquid‐to‐powder conversion: Probiotic delivery systems must remain active in the harsh gastrointestinal environment, where gastric acid, low pH, and enzymes can reduce probiotic viability or damage the system, lowering delivery efficiency.^[^ ^213^ ^]^ Currently, prepared probiotic systems are often “freshly prepared and used” liquid forms, characterized by high fluidity and difficulties in long‐term storage. These characteristics make them inconvenient for clinical use and patient administration and limit the possibility of large‐scale production and commercialization. Moreover, transitioning to stable forms like powders, capsules, or tablets is technically challenging, requiring protection of probiotic activity, stable drug loading, and optimized production processes. Existing technology remains immature, hindering the shift from lab‐scale liquids to practical clinical forms. If these problems cannot be solved, clinical translation will face bottlenecks.
4. Production and quality control standards: Currently, there is a lack of unified international or domestic standardized norms for the production of probiotic‐targeted delivery systems. Laboratory preparations often rely on researchers’ experience and conditions, making it difficult to form consistent quality control standards (such as detection methods for strain activity and drug loading efficiency). Clinical applications require strict batch consistency, quality traceability, and stability testing, but existing technology cannot meet these requirements, leading to difficulties in translating from laboratory to industrial production.
5. Dosage and potency standardization: The quality and potency of probiotics directly affect their therapeutic effect. The absence of standardized probiotic types, dosages, and treatment protocols affects the comparability of results. It is essential to validate the safety and efficacy of probiotic therapies through multicenter, large‐sample clinical trials and establish unified standards for probiotic types, dosages, and treatment protocols to improve result comparability.
6. Detection methodologies standardization: Detection methodologies for probiotics must be standardized to ensure comparability of results across different laboratories and manufacturers (e.g., qPCR for strain‐specific quantification and metabolomic profiling for functional validation). International organizations (e.g., ISO) could take the lead in developing globally applicable detection methodologies standards.
7. Safety and side effects: Not all probiotic strains are suitable for treating inflammatory diseases or cancer; some strains may cause adverse reactions. And not everyone can be treated with probiotics. For instance, cases of bacteremia associated with probiotic therapy have been documented in particularly vulnerable populations, such as very young and immunocompromised children.^[^ ^214^ ^]^ Moreover, the potential risks of long‐term or excessive probiotic use, such as microbial imbalance or immune overactivation, remain unclear.^[^ ^143a^ ^]^ Establishing a globally unified guideline for probiotic safety assessment (e.g., immunogenicity screening and long‐term microbiome monitoring) is recommended. Additional safety assessments should be conducted for vulnerable populations such as immunocompromised patients and children.
8. Market access and regulatory coordination. Diverse regulatory requirements across regions complicate market entry. Enhanced international coordination could unify global standards for probiotic products by drawing from the EU's EFSA (European Food Safety Authority) or the US's FDA (Food and Drug Administration).

### 5.2 Limitations to the Development of Probiotics Oral Delivery Systems

1. Technical bottlenecks in delivery systems: Current delivery technologies (e.g., microencapsulation, nanoparticles) may protect probiotics but could also affect their release and activity.
2. Functional limitations of probiotics: Probiotics' anti‐inflammatory and anti‐cancer mechanisms are not fully understood, limiting their precise application. And more importantly, probiotics alone may not address complex inflammatory or cancer‐related issues and may need to be combined with other therapies.

### 5.3 Future Perspectives of the Development of Probiotics Oral Delivery Systems

1. Development of novel delivery technologies: First, nanomaterials are being employed to effectively enhance probiotics' targeting and stability. For instance, encapsulation materials such as MPNs have been extensively investigated for their potential in surface film formation.^[^ ^185^, ^215^ ^]^ Second, responsive delivery systems that trigger probiotics release based on specific intestinal conditions are under active research. A notable approach involves single‐cell encapsulations conjugated with targeting moieties such as ligands or antibodies, enabling precise targeting of receptors overexpressed on CRC cells. Several researchers employed aromatic TA or a single thioether linkage, known for its specific responsiveness to ROS, thereby facilitating rapid drug release and enabling improvement of drug bioavailability. Third, addressing concerns related to chronic toxicity associated with prolonged use has led to an increasing trend of incorporating plant‐derived components into formulations, which not only mitigate potential toxicities but also maintain and potentially enhance the delivery system's efficacy through improved bioavailability.
2. Introduce automated production technologies and intelligent equipment, such as using microfluidics or high‐precision bioreactors for probiotic cultivation combined with sensors to monitor culture conditions (temperature, pH, oxygen concentration), to reduce human operational errors. Develop detailed Standard Operating Procedures (SOPs) and ensure consistency in the activity of probiotics, drug‐loading efficiency, and delivery system performance through process parameter optimization and real‐time quality monitoring.
3. Optimize the freeze‐drying technology by adding protectants or using coating materials that are resistant to freeze‐drying, to extend the formulation's shelf life and address the limitations of the “prepare‐and‐use” approach.
4. Personalized treatment: Given the differences in gut microbiota composition and tumor microenvironments among CRC patients, future research should use metagenomics and other technologies to analyze patients' gut microbiota and develop personalized probiotic treatment plans.
5. Interdisciplinary collaboration and technological innovation: As an emergent interdisciplinary discipline, its advancement necessitates collaborative efforts across the domains of microbiology, materials science, immunology, and clinical medicine. And it is also necessary to apply new technologies like CRISPR gene editing to engineer probiotics and enhance CRC therapeutic effects.
6. Combination therapy strategies: Probiotics combined with small‐molecular drugs or immune checkpoint inhibitors may improve efficacy. It is a good idea to implement multilayered gut microecological regulation strategies that combine dietary interventions, prebiotics, and postbiotics.
7. In‐depth research on probiotic mechanisms, especially their anti‐inflammatory, immunomodulatory, and anti‐cancer effects in CRC, is crucial for providing a theoretical basis for precision therapy.

## 6 Conclusion

The growing appreciation of probiotics' potential in the prevention and adjuvant treatment of CRC has sparked a surge of interest in developing sophisticated targeted‐delivery systems. These systems are designed to encapsulate, protect, and effectively deliver probiotics to the colon. This article provides a comprehensive review of the advancements in targeted probiotics release for CRC treatment, highlighting the various encapsulation methods available, each with its own set of advantages and limitations. Ranging from hydrogel microcapsule technology to the bulk encapsulation of probiotics with nanofibers, and more recently, the development of nano “armor” for a single probiotic through nanocoating, nanocarriers offer an innovative approach to probiotics encapsulation and delivery. It is important to mention that single‐cell encapsulation technologies offer significant advantages over traditional microencapsulation methods. Coated probiotics, for instance, are biocompatible and exhibit enhanced resistance to the harsh environment of the gastrointestinal tract. They can adhere to mucus, thereby prolonging their retention time, and promoting intestine colonization. They also enable improved controlled release of probiotic cells in the colon and hold potential for cellular‐level disease prevention and treatment. Consequently, the development of well‐designed, edible delivery systems could totally increase the effectiveness of probiotics in both preventing and treating CRC. These advancements in targeted delivery protect the probiotics from the hostile gastrointestinal environment and ensure their efficient delivery to the site of action, making them a promising tool in the arsenal against CRC.

## Conflict of Interest

The authors declare no conflict of interest.

## Author Contributions

H.Z. and J.J. contributed equally to this work and share the first authorship. H.Z. was responsible for writing the original draft, providing resources, contributing to methodology, conceptualizing the project, and administering the project. J.J. was involved in writing the original draft, reviewing and editing the writing, and providing resources. H.X.Z., M.H., and L.C. contributed to writing the review and editing. R.G. supervised the project, contributed to the methodology, and was involved in writing the review and editing.

## References

[1] R. L. Siegel , N. S. Wagle , A. Cercek , R. A. Smith , A. Jemal , Ca‐Cancer J. Clin. 2023, 73, 233. 36856579 10.3322/caac.21772

[2] I. Mármol , C. Sánchez‐de‐Diego , A. Pradilla Dieste , E. Cerrada , M. R Yoldi , Int. J. Mol. Sci. 2017, 18, 197. 28106826 10.3390/ijms18010197 PMC5297828

[3] Y. Cheng , Z. Ling , L. Li , Frontiers in Immunology 2020, 11, 615056. 33329610 10.3389/fimmu.2020.615056 PMC7734048

[4] J. Yu , Q. Feng , S. H. Wong , D. Zhang , Q. y. Liang , Y. Qin , L. Tang , H. Zhao , J. Stenvang , Y. Li , X. Wang , X. Xu , N. Chen , W. K. K. Wu , J. Al‐Aama , H. J. Nielsen , P. Kiilerich , B. A. H. Jensen , T. O. Yau , Z. Lan , H. Jia , J. Li , L. Xiao , T. Y. T. Lam , S. C. Ng , A. S.‐L. Cheng , V. W.‐S. Wong , F. K. L. Chan , X. Xu , H. Yang , et al., Gut 2017, 66, 70. 26408641 10.1136/gutjnl-2015-309800

[5] a) N. Sugimura , Q. Li , E. S. H. Chu , H. C. H. Lau , W. Fong , W. Liu , C. Liang , G. Nakatsu , A. C. Y. Su , O. O. Coker , W. K. K. Wu , F. K. L. Chan , J. Yu , Gut 2022, 71, 2011; 10.1136/gutjnl-2020-323951 PMC9484392 34937766

[6] S. Singh , M. Singh , S. Gaur , Frontiers in Immunology 2022, 13, 1002674. 36263037 10.3389/fimmu.2022.1002674 PMC9573965

[7] M. Zhou , W. Yuan , B. Yang , W. Pei , J. Ma , Q. Feng , Ann. Transl. Med. 2022, 10, 478. 35571406 10.21037/atm-22-1670 PMC9096358

[8] a) Q. Zhang , Q. Zhao , T. Li , L. Lu , F. Wang , H. Zhang , Z. Liu , H. Ma , Q. Zhu , J. Wang , X. Zhang , Y. Pei , Q. Liu , Y. Xu , J. Qie , X. Luan , Z. Hu , X. Liu , Cell Metab. 2023, 35, 943; 37192617 10.1016/j.cmet.2023.04.015

[9] W. Tang , W. Ma , T. Meng , W. Han , C. Liu , X. Duan , ACS Appl. Polym. Mater 2022, 4, 1368.

[10] M. Wang , J. Yang , M. Li , Y. Wang , H. Wu , L. Xiong , Q. Sun , Food Chem. 2019, 285, 260. 30797343 10.1016/j.foodchem.2019.01.162

[11] J. H. Park , K. Kim , J. Lee , J. Y. Choi , D. Hong , S. H. Yang , F. Caruso , Y. Lee , I. S. Choi , Angew. Chem., Int. Ed. 2014, 53, 12420. 10.1002/anie.201405905 25139382

[12] S. Ha , X. Zhang , J. Yu , Chin. Med. J. 2023, 137, 8. 38031348 10.1097/CM9.0000000000002955 PMC10766304

[13] M. Naeem , U. A. Awan , F. Subhan , J. Cao , S. P. Hlaing , J. Lee , E. Im , Y. Jung , J.‐W. Yoo , Arch. Pharmacal Res. 2020, 43, 153. 10.1007/s12272-020-01219-0 31989477

[14] N. Li , B. Lu , C. Luo , J. Cai , M. Lu , Y. Zhang , H. Chen , M. Dai , Cancer Letters 2021, 522, 255. 34563640 10.1016/j.canlet.2021.09.034

[15] R. M. McQuade , V. Stojanovska , R. Abalo , J. C. Bornstein , K. Nurgali , Front. Pharmacol. 2016, 7, 414. 27857691 10.3389/fphar.2016.00414 PMC5093116

[16] S. Xu , H. Lan , C. Huang , X. Ge , J. Zhu , Eur. J. Pharmacol. 2024, 974, 176614. 38677535 10.1016/j.ejphar.2024.176614

[17] M. Motoori , K. Sugimura , K. Tanaka , O. Shiraishi , Y. Kimura , H. Miyata , M. Yamasaki , T. Makino , Y. Miyazaki , M. Iwama , K. Yamashita , M. Niikura , T. Sugimoto , T. Asahara , K. Fujitani , T. Yasuda , Y. Doki , M. Yano , Clin. Nutr. 2022, 41, 1112. 35413573 10.1016/j.clnu.2022.03.023

[18] M. Mego , J. Chovanec , I. Vochyanova‐Andrezalova , P. Konkolovsky , M. Mikulova , M. Reckova , V. Miskovska , B. Bystricky , J. Beniak , L. Medvecova , A. Lagin , D. Svetlovska , S. Spanik , V. Zajac , J. Mardiak , L. Drgona , Complementary Therapies in Medicine 2015, 23, 356. 26051570 10.1016/j.ctim.2015.03.008

[19] H. Hassan , M. Rompola , A. W. Glaser , S. E. Kinsey , R. S. Phillips , Supportive Care in Cancer 2018, 26, 2503. 29704110 10.1007/s00520-018-4216-z

[20] E. L. Amitay , P. R. Carr , A. Gies , D. C. Laetsch , H. Brenner , Clin. Transl. Gastroenterol. 2020, 11, 00268. 10.14309/ctg.0000000000000268 PMC7714058 33512803

[21] T. Tanoue , S. Morita , D. R. Plichta , A. N. Skelly , W. Suda , Y. Sugiura , S. Narushima , H. Vlamakis , I. Motoo , K. Sugita , A. Shiota , K. Takeshita , K. Yasuma‐Mitobe , D. Riethmacher , T. Kaisho , J. M. Norman , D. Mucida , M. Suematsu , T. Yaguchi , V. Bucci , T. Inoue , Y. Kawakami , B. Olle , B. Roberts , M. Hattori , R. J. Xavier , K. Atarashi , K. Honda , Nature 2019, 565, 600. 30675064 10.1038/s41586-019-0878-z

[22] Y. He , L. Fu , Y. Li , W. Wang , M. Gong , J. Zhang , X. Dong , J. Huang , Q. Wang , C. R. Mackay , Y.‐X. Fu , Y. Chen , X. Guo , Cell Metab. 2021, 33, 988. 33761313 10.1016/j.cmet.2021.03.002

[23] Z. Ren , S. Chen , H. Lv , L. Peng , W. Yang , J. Chen , Z. Wu , C. Wan , Pharmacological Research 2022, 184, 106406. 35987480 10.1016/j.phrs.2022.106406

[24] F. Huang , S. Li , W. Chen , Y. Han , Y. Yao , L. Yang , Q. Li , Q. Xiao , J. Wei , Z. Liu , T. Chen , X. Deng , Nutrients 2023, 15, 356. 36678227 10.3390/nu15020356 PMC9861237

[25] Z. He , H. Xie , H. Xu , J. Wu , W. Zeng , Q. He , C. Jobin , S. Jin , P. Lan , Gut Microbes 2024, 16, 2319511. 38400752 10.1080/19490976.2024.2319511 PMC10896127

[26] a) D. E. Citrin , D. L. Longo , N. Engl. J. Med. 2017, 377, 1065; 28902591 10.1056/NEJMra1608986

[27] H. Wang , X. Mu , H. He , X.‐D. Zhang , Trends Pharmacol. Sci. 2018, 39, 24. 29224916 10.1016/j.tips.2017.11.003

[28] Q. Hua , H. Zhang , R. Xu , C. Tian , T. Gao , Y. Yuan , Y. Han , Y. Li , C. Qi , F. Zhong , A. Ma , Mol. Nutr. Food Res. 2022, 67, 2200337. 10.1002/mnfr.202200337 36408889

[29] M. G. Seal , Y. Naito , R. Barreto , A. Lorenzetti , P. Safran , F. Marotta , Journal of Digestive Diseases 2007, 8, 143. 17650226 10.1111/j.1443-9573.2007.00301.x

[30] P. Delia , G. Sansotta , V. Donato , G. Messina , P. Frosina , S. Pergolizzi , C. De Renzis , G. Famularo , The American Journal of Gastroenterology 2002, 97, 2150. 12190202 10.1111/j.1572-0241.2002.05946.x

[31] E. Kouhsari , A. Ghadimi‐Daresajini , H. Abdollahi , N. Amirmozafari , S. R. Mahdavi , S. Abbasian , S. H. Mousavi , H. F. Yaseri , M. Moghaderi , Clin. Transl. Oncol. 2017, 20, 127. 28623514 10.1007/s12094-017-1701-7

[32] a) M. Abdoli , G. Mohammadi , K. Mansouri , S. Khaledian , M. Taran , F. Martinez , J. Biotechnol. 2022, 354, 63; 35724764 10.1016/j.jbiotec.2022.06.005

[33] a) F. Aragón , S. Carino , G. Perdigón , A. de Moreno de LeBlanc , Immunobiology 2014, 219, 457; 24646876 10.1016/j.imbio.2014.02.005

[34] G. Gao , S. Shen , T. Zhang , J. Zhang , S. Huang , Z. Sun , H. Zhang , eBioMedicine 2023, 91, 104533. 37027929 10.1016/j.ebiom.2023.104533 PMC10085781

[35] X. Deng , J. Yang , Y. Zhang , X. Chen , C. Wang , H. Suo , J. Song , Foods 2023, 12, 3706. 37835359 10.3390/foods12193706 PMC10572180

[36] F. Xu , Q. Li , S. Wang , J. Bai , M. Dong , G. Xiao , J. Wang , Journal of Functional Foods 2021, 87, 104779.

[37] G. J. Nychas , A. Tiptiri‐Kourpeti , K. Spyridopoulou , V. Santarmaki , G. Aindelis , E. Tompoulidou , E. E. Lamprianidou , G. Saxami , P. Ypsilantis , E. S. Lampri , C. Simopoulos , I. Kotsianidis , A. Galanis , Y. Kourkoutas , D. Dimitrellou , K. Chlichlia , PLoS One 2016, 11, e0147960. 26849051 10.1371/journal.pone.0147960 PMC4744000

[38] K. Śliżewska , P. Markowiak‐Kopeć , W. Śliżewska , Cancers 2020, 13, 20. 33374549 10.3390/cancers13010020 PMC7793079

[39] X. Kang , C. Liu , Y. Ding , Y. Ni , F. Ji , H. C. H. Lau , L. Jiang , J. J. Y. Sung , S. H. Wong , J. Yu , Gut 2023, 72, 2112. 37491158 10.1136/gutjnl-2023-330291 PMC10579466

[40] T. A. Oelschlaeger , International Journal of Medical Microbiology 2010, 300, 57. 19783474 10.1016/j.ijmm.2009.08.005

[41] G. Keyhani , H. M. Hosseini , A. Salimi , Iranian journal of microbiology 2022, 14, 90. 35664711 10.18502/ijm.v14i1.8809 PMC9085540

[42] Y. Shi , L. Meng , C. Zhang , F. Zhang , Y. Fang , Microbiological Research 2022, 255, 126921. 10.1016/j.micres.2021.126921 34839170

[43] B. C. An , Y. Ryu , Y.‐S. Yoon , O. Choi , H. J. Park , T. Y. Kim , S.‐I. Kim , B.‐K. Kim , M. J. Chung , Mol. Cells 2019, 42, 755. 31707776 10.14348/molcells.2019.0064 PMC6883978

[44] C. Hill , F. Guarner , G. Reid , G. R. Gibson , D. J. Merenstein , B. Pot , L. Morelli , R. B. Canani , H. J. Flint , S. Salminen , P. C. Calder , M. E. Sanders , Nat. Rev. Gastroenterol. Hepatol. 2014, 11, 506. 24912386 10.1038/nrgastro.2014.66

[45] D. Kothari , S. Patel , S.‐K. Kim , Biomed. Pharmacother. 2019, 111, 537. 30597307 10.1016/j.biopha.2018.12.104

[46] S. Ciernikova , M. Mego , M. Semanova , L. Wachsmannova , Z. Adamcikova , V. Stevurkova , L. Drgona , V. Zajac , Integrative Cancer Therapies 2016, 16, 188. 27151581 10.1177/1534735416643828 PMC5739119

[47] S. Hempel , S. Newberry , A. Ruelaz , Z. Wang , J. N. V. Miles , M. J. Suttorp , B. Johnsen , S. Roberta , W. Slusser , N. Fu , A. Smith , B. Roth , J. Polak , A. Motala , T. Perry , P. G. Shekelle , Evidence Report/Technology Assessment 2011, 200, 1. PMC4780970 23126627

[48] Y. H. Wang , N. Yao , K. K. Wei , L. Jiang , S. Hanif , Z. X. Wang , C. X. Pei , European Journal of Clinical Nutrition 2016, 70, 1246. 27329608 10.1038/ejcn.2016.102

[49] S. Rau , A. Gregg , S. Yaceczko , B. Limketkai , Nutrients 2024, 16, 778. 38542689 10.3390/nu16060778 PMC10975713

[50] K. Wang , W. Li , X. Rui , X. Chen , M. Jiang , M. Dong , Int. J. Biol. Macromol. 2014, 63, 133. 24189393 10.1016/j.ijbiomac.2013.10.036

[51] A. B. Leila , H. B. Leila , A. Parinaz , A. Parinaz , L. zarei , Medical Science 2018, 22, 99.

[52] S. M. A. Aziz Mousavi , S. A. Mirhosseini , M. R Shariat Panahi , H. M Hosseini , Probiotics and Antimicrobial Proteins 2019, 12, 740. 10.1007/s12602-019-09530-z 31020619

[53] R. Manikandan , B. Manikandan , T. Raman , K. Arunagirinathan , N. M. Prabhu , M. J Basu , M. Perumal , S. Palanisamy , A. Munusamy , Spectrochim. Acta, Part A 2015, 138, 120. 10.1016/j.saa.2014.10.043 25481491

[54] K. Spyridopoulou , E. Tryfonopoulou , G. Aindelis , P. Ypsilantis , C. Sarafidis , O. Kalogirou , K. Chlichlia , Nanoscale Advances 2021, 3, 2516. 36134160 10.1039/d0na00984a PMC9417964

[55] A. B. A. Mohammed , A. E. Hegazy , A. Salah , Appl. Nanosci. 2021, 13, 633.

[56] A. Gupta , S. Mumtaz , C.‐H. Li , I. Hussain , V. M. Rotello , Chem. Soc. Rev. 2019, 48, 415. 30462112 10.1039/c7cs00748e PMC6340759

[57] H. Tang , T. Zhou , W. Jin , S. Zong , T. Mamtimin , E.‐S. Salama , B.‐H. Jeon , P. Liu , H. Han , X. Li , Life Sci. 2023, 324, 121709. 37100380 10.1016/j.lfs.2023.121709

[58] J. Zhou , M. Li , Q. Chen , X. Li , L. Chen , Z. Dong , W. Zhu , Y. Yang , Z. Liu , Q. Chen , Nat. Commun. 2022, 13, 3432. 35701435 10.1038/s41467-022-31171-0 PMC9198027

[59] S. Gu , X. Zhao , F. Wan , D. Gu , W. Xie , C. Gao , Nano Lett. 2024, 24, 13504. 39418594 10.1021/acs.nanolett.4c02699

[60] F. Cao , L. Jin , C. Zhang , Y. Gao , Z. Qian , H. Wen , S. Yang , Z. Ye , L. Hong , H. Yang , Z. Tong , L. Cheng , Y. Ding , W. Wang , G. Yu , Z. Mao , X. Chen , Adv. Mater. 2023, 36, 2304257. 10.1002/adma.202304257 37788635

[61] Y. J. Jang , H. M. Gwon , W. S. Jeong , S. H. Yeo , S. Y. Kim , Microorganisms 2021, 9, 2450. 34946052 10.3390/microorganisms9122450 PMC8704421

[62] H. S. Yu , N. K. Lee , A. J. Choi , J. S. Choe , C. H. Bae , H. D. Paik , Journal of Microbiology and Biotechnology 2019, 29, 1022. 31216608 10.4014/jmb.1903.03014

[63] Y. Yang , L. Li , C. Xu , Y. Wang , Z. Wang , M. Chen , Z. Jiang , J. Pan , C. Yang , X. Li , K. Song , J. Yan , W. Xie , X. Wu , Z. Chen , Y. Yuan , S. Zheng , J. Yan , J. Huang , F. Qiu , Gut 2021, 70, 1495. 10.1136/gutjnl-2020-320777 PMC8292576 33122176

[64] a) L. Chen , H. Deng , H. Cui , J. Fang , Z. Zuo , J. Deng , Y. Li , X. Wang , L. Zhao , Oncotarget 2018, 9, 7204; 29467962 10.18632/oncotarget.23208 PMC5805548

[65] a) T. Wang , L. Zhang , P. Wang , Y. Liu , G. Wang , Y. Shan , Y. Yi , Y. Zhou , B. Liu , X. Wang , X. Lü , Eur. J. Nutr. 2021, 61, 85; 34185157 10.1007/s00394-021-02627-8

[66] T. Wang , P. Wang , W. Ge , C. Shi , G. Xiao , X. Wang , X. Lü , Food Bioscience 2021, 44, 101346.

[67] M. C. S. Mendes , D. S. M. Paulino , S. R. Brambilla , J. A. Camargo , G. F. Persinoti , J. B. C. Carvalheira , World J. Gastroenterol. 2018, 24, 1995. 29760543 10.3748/wjg.v24.i18.1995 PMC5949713

[68] a) B. K. Popivanova , K. Kitamura , Y. Wu , T. Kondo , T. Kagaya , S. Kaneko , M. Oshima , C. Fujii , N. Mukaida , J. Clin. Invest. 2008, 118, 560; 10.1172/JCI32453 PMC2213370 18219394

[69] a) H. Ding , Q. Mei , H. Z. Gan , L. Y. Cao , X. C. Liu , J. M. Xu , Gastroenterology Report 2014, 2, 215; 24787389 10.1093/gastro/gou022 PMC4124268

[70] S. C. Bischoff , G. Barbara , W. Buurman , T. Ockhuizen , J.‐D. Schulzke , M. Serino , H. Tilg , A. Watson , J. M. Wells , BMC gastroenterology 2014, 14, 1. 25407511 10.1186/s12876-014-0189-7 PMC4253991

[71] S. Sánchez‐Fidalgo , I. Villegas , A. Cárdeno , E. Talero , M. Sánchez‐Hidalgo , V. Motilva , C. Alarcón de la Lastra , Clin. Nutr. 2010, 29, 663. 20427102 10.1016/j.clnu.2010.03.003

[72] K. Watanabe , T. Kawamori , S. Nakatsugi , K. Wakabayashi , BioFactors 2008, 12, 129. 10.1002/biof.5520120120 11216473

[73] M. Peng , D. Biswas , Crit. Rev. Food Sci. Nutr. 2016, 57, 3987. 10.1080/10408398.2016.1203286 27438132

[74] M. Peng , S.‐H. Lee , S. O. Rahaman , D. Biswas , Food Funct. 2020, 11, 10724. 33231228 10.1039/d0fo02652b

[75] P. Jin , S. Civini , Y. Zhao , V. De Giorgi , J. Ren , M. Sabatino , J. Jin , H. Wang , D. Bedognetti , F. Marincola , D. Stroncek , Br. J. Cancer 2014, 110, 2955. 24809778 10.1038/bjc.2014.235 PMC4056054

[76] M. P. Rubinstein , E. W. Su , S. Suriano , C. A. Cloud , K. Andrijauskaite , P. Kesarwani , K. M. Schwartz , K. M. Williams , C. B. Johnson , M. Li , G. M. Scurti , M. L. Salem , C. M. Paulos , E. Garrett‐Mayer , S. Mehrotra , D. J. Cole , Cancer Immunology, Immunotherapy 2015, 64, 539. 25676709 10.1007/s00262-015-1655-y PMC4804872

[77] F. Ma , M. Sun , Y. Song , A. Wang , S. Jiang , F. Qian , G. Mu , Y. Tuo , Nutrients 2022, 14, 1916. 35565884 10.3390/nu14091916 PMC9100115

[78] S. L. Zhang , B. Han , Y. Q. Mao , Z. Y. Zhang , Z. M. Li , C. Y. Kong , Y. Wu , G. Q. Chen , L. S. Wang , Gut Microbes 2022, 14, 2046246. 35259052 10.1080/19490976.2022.2046246 PMC8920197

[79] T. J. Zumwalt , M. Arnold , A. Goel , C. R. Boland , Oncotarget 2014, 6, 2981. 10.18632/oncotarget.3205 PMC4413778 25671296

[80] B. Farhood , M. Najafi , K. Mortezaee , J. Cell. Physiol. 2018, 234, 8509. 30520029 10.1002/jcp.27782

[81] G. Aindelis , A. Tiptiri‐Kourpeti , E. Lampri , K. Spyridopoulou , E. Lamprianidou , I. Kotsianidis , P. Ypsilantis , A. Pappa , K. Chlichlia , Cancers 2020, 12, 368. 32033490 10.3390/cancers12020368 PMC7072577

[82] X. Zhang , D. Yu , D. Wu , X. Gao , F. Shao , M. Zhao , J. Wang , J. Ma , W. Wang , X. Qin , Y. Chen , P. Xia , S. Wang , Cell Host Microbe 2023, 31, 418. 36893736 10.1016/j.chom.2023.01.013

[83] M. E. Griffin , E. Juliel , J. L. Becker , L. Dung , Ji, T. S. C , J. K. Jha , G. R. Fanger , H. C. Hang , Science 2021, 373, 1040. 34446607 10.1126/science.abc9113 PMC9503018

[84] W. Fong , Q. Li , F. Ji , W. Liang , H. C. H. Lau , X. Kang , W. Liu , K. K.‐W. To , Z. Zuo , X. Li , X. Zhang , J. J. Sung , Y. Jun , Gut 2023, 72, 2272. 37770127 10.1136/gutjnl-2023-329543 PMC10715476

[85] Q. Zhuo , B. Yu , J. Zhou , J. Zhang , R. Zhang , J. Xie , Q. Wang , S. Zhao , Sci. Rep. 2019, 9, 20128. 31882868 10.1038/s41598-019-56661-y PMC6934597

[86] L. Shi , J. Sheng , G. Chen , P. Zhu , C. Shi , B. Li , C. Park , J. Wang , B. Zhang , Z. Liu , X. Yang , Journal for ImmunoTherapy of Cancer 2020, 8, 000973. 10.1136/jitc-2020-000973 PMC7542661 33028692

[87] J. A. Owens , B. J. Saeedi , C. R. Naudin , S. Hunter‐Chang , M. E. Barbian , R. U. Eboka , L. Askew , T. M. Darby , B. S. Robinson , R. M. Jones , Cellular and Molecular Gastroenterology and Hepatology 2021, 12, 1311. 34111601 10.1016/j.jcmgh.2021.06.001 PMC8463873

[88] Y. Gao , P. Xu , D. Sun , Y. Jiang , X.‐L. Lin , T. Han , J. Yu , C. Sheng , H. Chen , J. Hong , Y. Chen , X.‐Y. Xiao , J.‐Y. Fang , Cancer Res. 2023, 83, 3710. 37602831 10.1158/0008-5472.CAN-23-0605

[89] D. Chen , D. Jin , S. Huang , J. Wu , M. Xu , T. Liu , W. Dong , X. Liu , S. Wang , W. Zhong , Y. Liu , R. Jiang , M. Piao , B. Wang , H. Cao , Cancer Letters 2020, 469, 456. 31734354 10.1016/j.canlet.2019.11.019

[90] L. K. Sharaf , M. Sharma , D. Chandel , G. Shukla , BMC Cancer 2018, 18, 1111. 30424722 10.1186/s12885-018-4999-9 PMC6234654

[91] S. Zununi Vahed , A. Barzegari , Y. Rahbar Saadat , A. Goreyshi , Y. Omidi , Biomed. Pharmacother. 2017, 94, 1094. 28821160 10.1016/j.biopha.2017.08.033

[92] L. Zhou , Y. Zhao , Cancer Manage. Res. 2019, 11, 10205. 10.2147/CMAR.S222224 PMC6899073 31819652

[93] L. Scorrano , S. J. Korsmeyer , Biochem. Biophys. Res. Commun. 2003, 304, 437. 12729577 10.1016/s0006-291x(03)00615-6

[94] P. J. Kahrilas , Gastroenterology 2010, 139, 716. 20659461 10.1053/j.gastro.2010.07.014 PMC3027213

[95] R. Ghanavati , P. Asadollahi , M. B. Shapourabadi , S. Razavi , M. Talebi , M. Rohani , Microbial Pathogenesis 2020, 139, 103829. 31682995 10.1016/j.micpath.2019.103829

[96] M. Ragusa , L. Statello , M. Maugeri , A. Majorana , D. Barbagallo , L. Salito , M. Sammito , M. Santonocito , R. Angelica , A. Cavallaro , M. Scalia , R. Caltabiano , G. Privitera , A. Biondi , M. Di Vita , A. Cappellani , E. Vasquez , S. Lanzafame , E. Tendi , S. Celeste , C. Di Pietro , F. Basile , M. Purrello , Journal of Molecular Medicine 2012, 90, 1421. 22660396 10.1007/s00109-012-0918-8

[97] A. Isazadeh , S. Hajazimian , B. Shadman , S. Safaei , A. Babazadeh Bedoustani , R. Chavoshi , D. Shanehbandi , M. Mashayekhi , M. Nahaei , B. Baradaran , Pharmaceutical Sciences 2020, 27, 262.

[98] E. Jacouton , F. Chain , H. Sokol , P. Langella , L. G. Bermúdez‐Humarán , Frontiers in Immunology 2017, 8, 1553. 29209314 10.3389/fimmu.2017.01553 PMC5702231

[99] B. S. Nielsen , S. Jørgensen , J. U. Fog , R. Søkilde , I. J. Christensen , U. Hansen , N. Brünner , A. Baker , S. Møller , H. J. Nielsen , Clin. Exp. Metastasis 2010, 28, 27. 21069438 10.1007/s10585-010-9355-7 PMC2998639

[100] C. A. Fahmy , A. M. Gamal‐Eldeen , E. A. El‐Hussieny , B. M. Raafat , N. S. Mehanna , R. M. Talaat , M. T. Shaaban , Nutrition and Cancer 2019, 71, 688. 30862187 10.1080/01635581.2019.1577984

[101] M. B. Zeisel , P. Dhawan , T. F. Baumert , Gut 2019, 68, 547. 30297438 10.1136/gutjnl-2018-316906 PMC6453741

[102] Y. Chen , S. Lin , L. Wang , Y. Zhang , H. Chen , Z. Fu , M. Zhang , H. Luo , J. Liu , Nat. Biomed. Eng. 2024, 8, 823. 38839928 10.1038/s41551-024-01224-4

[103] S.‐Y. Lee , B.‐H. Lee , J.‐H. Park , M.‐S. Park , G.‐E. Ji , M.‐K. Sung , Journal of Medicinal Food 2022, 25, 146. 35148194 10.1089/jmf.2021.K.0150

[104] J. Sun , I. Kato , Genes & Diseases 2016, 3, 130. 28078319 10.1016/j.gendis.2016.03.004 PMC5221561

[105] M. Li , B. C. A. M. Van Esch , G. T. M. Wagenaar , J. Garssen , G. Folkerts , P. A. J. Henricks , Eur. J. Pharmacol. 2018, 831, 52. 29750914 10.1016/j.ejphar.2018.05.003

[106] P. V. Chang , L. Hao , S. Offermanns , R. Medzhitov , Proc. Natl. Acad. Sci. USA 2014, 111, 2247. 24390544 10.1073/pnas.1322269111 PMC3926023

[107] S. Y. Archer , S. Meng , A. Shei , H. RA , Proc Natl Acad Sci U S A. 1998, 95, 6791. 9618491 10.1073/pnas.95.12.6791 PMC22637

[108] N. Singh , A. Gurav , S. Sivaprakasam , E. Brady , R. Padia , H. Shi , M. Thangaraju , P D. Prasad , S. Manicassamy , D H. Munn , J R. Lee , S. Offermanns , V. Ganapathy , Immunity 2014, 40, 128. 24412617 10.1016/j.immuni.2013.12.007 PMC4305274

[109] a) K. Wang , M. Karin , Cell. Logist. 2014, 3, e24975; 10.4161/cl.24975 PMC3906427 24516778

[110] D. Song , X. Wang , Y. Ma , N.‐N. Liu , H. Wang , Front. Nutr. 2023, 10, 1111872. 36969804 10.3389/fnut.2023.1111872 PMC10036377

[111] L. Wang , L. Tang , Y. Feng , S. Zhao , M. Han , C. Zhang , G. Yuan , J. Zhu , S. Cao , Q. Wu , L. Li , Z. Zhang , Gut 2020, 69, 1988. 32169907 10.1136/gutjnl-2019-320105 PMC7569398

[112] H. Kawanabe‐Matsuda , K. Takeda , M. Nakamura , S. Makino , T. Karasaki , K. Kakimi , M. Nishimukai , T. Ohno , J. Omi , K. Kano , A. Uwamizu , H. Yagita , I. G. Boneca , G. Eberl , J. Aoki , M. J. Smyth , K. Okumura , Cancer Discovery 2022, 12, 1336. 35180303 10.1158/2159-8290.CD-21-0929 PMC9662940

[113] S. Sheng , Y. Fu , N. Pan , H. Zhang , L. Xiu , Y. Liang , Y. Liu , B. Liu , C. Ma , R. Du , X. Wang , Frontiers in Microbiology 2022, 13, 1015270. 36225355 10.3389/fmicb.2022.1015270 PMC9549278

[114] H. N. Bell , R. J. Rebernick , J. Goyert , R. Singhal , M. Kuljanin , S. A. Kerk , W. Huang , N. K. Das , A. Andren , S. Solanki , S. L. Miller , P. K. Todd , E. R. Fearon , C. A. Lyssiotis , S. P. Gygi , J. D. Mancias , Y. M. Shah , Cancer Cell 2022, 40, 185. 34951957 10.1016/j.ccell.2021.12.001 PMC8847337

[115] H. Konishi , M. Fujiya , H. Tanaka , N. Ueno , K. Moriichi , J. Sasajima , K. Ikuta , H. Akutsu , H. Tanabe , Y. Kohgo , Nat. Commun. 2016, 7, 12365. 27507542 10.1038/ncomms12365 PMC4987524

[116] C. C. Wong , J. Yu , Nat. Rev. Clin. Oncol. 2023, 20, 429. 37169888 10.1038/s41571-023-00766-x

[117] X. Li , S. Zhang , G. Guo , J. Han , J. Yu , eBioMedicine 2022, 82, 104163. 35841869 10.1016/j.ebiom.2022.104163 PMC9297075

[118] X. Ji , C. Hou , Y. Gao , Y. Xue , Y. Yan , X. Guo , Food Funct. 2020, 11, 163. 31830158 10.1039/c9fo02171j

[119] Q. Li , W. Hu , W. X. Liu , L. Y. Zhao , D. Huang , X. D. Liu , H. Chan , Y. Zhang , J. D. Zeng , O. O. Coker , W. Kang , S. S. M. Ng , L. Zhang , S. H. Wong , T. Gin , M. T. V. Chan , J. L. Wu , J. Yu , W. K. K. Wu , Gastroenterology 2021, 160, 1179. 32920015 10.1053/j.gastro.2020.09.003

[120] A. U. Rehman , A. Iqbal Khan , Y. Xin , W. Yousuf , Ahmad, W. L , Medicine in Microecology 2022, 14, 100062.

[121] S. Fukuda , H. Toh , K. Hase , K. Oshima , Y. Nakanishi , K. Yoshimura , T. Tobe , J. M. Clarke , D. L. Topping , T. Suzuki , T. D. Taylor , K. Itoh , J. Kikuchi , H. Morita , M. Hattori , H. Ohno , Nature 2011, 469, 543. 21270894 10.1038/nature09646

[122] a) J. Chen , B. Kang , Q. Jiang , M. Han , Y. Zhao , L. Long , C. Fu , K. Yao , Front Microbiol 2018, 9, 1057; 29904374 10.3389/fmicb.2018.01057 PMC5991137

[123] E. Ansaldo , L. C. Slayden , K. L. Ching , M. A. Koch , N. K. Wolf , D. R. Plichta , E. M. Brown , D. B. Graham , R. J. Xavier , J. J. Moon , G. M. Barton , Science 2019, 364, 1179. 31221858 10.1126/science.aaw7479 PMC6645389

[124] A. C. Y. Su , X. Ding , H. C. H. Lau , X. Kang , Q. Li , X. Wang , Y. Liu , L. Jiang , Y. Lu , W. Liu , Y. Ding , A. H.‐K. Cheung , K. F. To , J. Yu , Gut 2024, 73, 1478. 38599786 10.1136/gutjnl-2023-330835 PMC11347254

[125] H. Tan , Q. Zhai , W. Chen , Food Research International 2019, 116, 637. 30716990 10.1016/j.foodres.2018.08.088

[126] a) T. Yasuma , M. Toda , A. M. Abdel‐Hamid , C. D'Alessandro‐Gabazza , T. Kobayashi , K. Nishihama , V. F. D'Alessandro , G. V. Pereira , R. I. Mackie , E. C. Gabazza , I. Cann , Microorganisms 2021, 9, 1126; 34067445 10.3390/microorganisms9061126 PMC8224763

[127] Y. Heshiki , R. Vazquez‐Uribe , J. Li , Y. Ni , S. Quainoo , L. Imamovic , J. Li , M. Sørensen , B. K. C. Chow , G. J. Weiss , A. Xu , M. O. A. Sommer , G. Panagiotou , Microbiome 2020, 8, 28. 32138779 10.1186/s40168-020-00811-2 PMC7059390

[128] G. Q. Gao , T. Ma , T. Zhang , H. Jin , Y. L. Li , L. Y. Kwok , H. P. Zhang , Z. H. Sun , Frontiers in Immunology 2021, 12, 663986.34122422

[129] a) M. Vétizou , J. M. Pitt , R. Daillère , P. Lepage , N. Waldschmitt , C. Flament , S. Rusakiewicz , B. Routy , M. P. Roberti , C. P. M. Duong , V. Poirier‐Colame , A. Roux , S. Becharef , S. Formenti , E. Golden , S. Cording , G. Eberl , A. Schlitzer , F. Ginhoux , S. Mani , T. Yamazaki , N. Jacquelot , D. P. Enot , M. Bérard , J. Nigou , P. Opolon , A. Eggermont , P.‐L. Woerther , E. Chachaty , N. Chaput , et al., Science 2015, 350, 1079; 26541610 10.1126/science.aad1329 PMC4721659

[130] J. Cheng , J. Hu , F. Geng , S. Nie , Food Science and Human Wellness 2022, 11, 1101.

[131] N. O. Kaakoush , Frontiers in Cellular and Infection Microbiology 2015, 5, 84. 26636046 10.3389/fcimb.2015.00084 PMC4653637

[132] a) J. Luo , C. Zhang , R. Liu , L. Gao , S. Ou , L. Liu , X. Peng , Journal of Functional Foods 2018, 47, 127;

[133] a) S. Wu , K.‐J. Rhee , E. Albesiano , S. Rabizadeh , X. Wu , H.‐R. Yen , D. L. Huso , F. L. Brancati , E. Wick , F. McAllister , F. Housseau , D. M. Pardoll , C. L. Sears , Nat. Med. 2009, 15, 1016; 19701202 10.1038/nm.2015 PMC3034219

[134] Y. Wu , J. Wu , T. Chen , Q. Li , W. Peng , H. Li , X. Tang , X. Fu , Dig. Dis. Sci. 2018, 63, 1210. 29508166 10.1007/s10620-018-4999-2

[135] J. Q. Liang , Y. Zeng , E. Y. T. Lau , Y. Sun , Y. Huang , T. Zhou , Z. Xu , J. Yu , S. C. Ng , F. K. L. Chan , Cells 2023, 12, 1244. 37174650 10.3390/cells12091244 PMC10177585

[136] a) L. Cassani , A. Gomez‐Zavaglia , J. Simal‐Gandara , Food Research International 2020, 129, 108852; 32036930 10.1016/j.foodres.2019.108852

[137] a) F. Centurion , S. Merhebi , M. Baharfar , R. Abbasi , C. Zhang , M. Mousavi , W. Xie , J. Yang , Z. Cao , F. M. Allioux , G. F. S. Harm , J. Biazik , K. Kalantar‐Zadeh , M. A. Rahim , Adv. Funct. Mater. 2022, 32, 2200775;

[138] a) X. Wang , Z. Cao , M. Zhang , L. Meng , Z. Ming , J. Liu , Sci. Adv. 2020, 6, eabb1952 ; 32637620 10.1126/sciadv.abb1952 PMC7314526

[139] a) K. Yang , X. Wang , R. Huang , H. Wang , P. Lan , Y. Zhao , Adv. Sci. 2022, 9, 2104089; 10.1002/advs.202104089 PMC9165482 35403829

[140] Z. Cao , X. Wang , Y. Pang , S. Cheng , J. Liu , Nat. Commun. 2019, 10, 5783. 31857577 10.1038/s41467-019-13727-9 PMC6923387

[141] D. W. Zheng , R. Q. Li , J. X. An , T. Q. Xie , Z. Y. Han , R. Xu , Y. Fang , X. Z. Zhang , Adv. Mater. 2020, 32, 2004529. 10.1002/adma.202004529 33006175

[142] L. A. Bosnea , T. Moschakis , C. G. Biliaderis , Food Funct. 2017, 8, 554. 27935607 10.1039/c6fo01019a

[143] a) C. Xu , M. A. Gantumur , J. L. Sun , J. H. Guo , J. G. Ma , Z. M. Jiang , W. Wang , J. Zhang , Y. Ma , J. C. Hou , D. J. McClements , Food Hydrocolloids 2024, 149, 109588;

[144] a) G. Multhoff , M. Molls , J. Radons , Frontiers in Immunology 2012, 2, 98; 22566887 10.3389/fimmu.2011.00098 PMC3342348

[145] F. R. Greten , S. I. Grivennikov , Immunity 2019, 51, 27. 31315034 10.1016/j.immuni.2019.06.025 PMC6831096

[146] a) H. Zhao , L. Wu , G. Yan , Y. Chen , M. Zhou , Y. Wu , Y. Li , Signal Transduction Targeted Ther. 2021, 6, 263; 10.1038/s41392-021-00658-5 PMC8273155 34248142

[147] F. J. Rodrigues , M. F. Cedran , J. L. Bicas , H. H. Sato , Food Research International 2020, 137, 109682. 33233258 10.1016/j.foodres.2020.109682

[148] T. Huq , C. Fraschini , A. Khan , B. Riedl , J. Bouchard , M. Lacroix , Carbohydr. Polym. 2017, 168, 61. 28457464 10.1016/j.carbpol.2017.03.032

[149] F. Centurion , A. W. Basit , J. Liu , S. Gaisford , M. A. Rahim , K. Kalantar‐Zadeh , ACS Nano 2021, 15, 18653. 34860008 10.1021/acsnano.1c09951

[150] X. Fu , Z. Liu , C. Zhu , H. Mou , Q. Kong , Crit. Rev. Food Sci. Nutr. 2018, 59, S130. 30580556 10.1080/10408398.2018.1542587

[151] Z. Zhang , M. Gu , X. You , D. A. Sela , H. Xiao , D. J. McClements , Food Hydrocolloids 2021, 116, 106634.

[152] a) X. Ding , Y. Xu , Y. Wang , L. Xie , S. Liang , D. Li , Y. Wang , J. Wang , X. Zhan , Carbohydr. Polym. 2022, 289, 119438; 35483851 10.1016/j.carbpol.2022.119438

[153] L. Niu , Y. Liu , N. Li , Y. Wang , L. Kang , X. Su , C. Xu , Z. Sun , W. Sang , J. Xu , H. Guo , S. Shen , Int. J. Pharm. 2024, 652, 123810. 38244648 10.1016/j.ijpharm.2024.123810

[154] N. Li , L. Niu , Y. Liu , Y. Wang , X. Su , C. Xu , Z. Sun , H. Guo , J. Gong , S. Shen , J. Nanobiotechnol. 2024, 22, 241. 10.1186/s12951-024-02506-4 PMC11089779 38735933

[155] M. Zhang , E. Viennois , M. Prasad , Y. Zhang , L. Wang , Z. Zhang , M. K. Han , B. Xiao , C. Xu , S. Srinivasan , D. Merlin , Biomaterials 2016, 101, 321. 27318094 10.1016/j.biomaterials.2016.06.018 PMC4921206

[156] P. K. Deol , P. Khare , D. P. Singh , G. Soman , M. Bishnoi , K. K. Kondepudi , I. P. Kaur , Int. J. Biol. Macromol. 2017, 105, 81. 28690172 10.1016/j.ijbiomac.2017.06.117

[157] Q. Chen , J. Qiao , M. Cao , Z. Han , X. Zeng , X. Zhang , Mater. Today 2023, 63, 32.

[158] Q. Luan , W. Zhou , H. Zhang , Y. Bao , M. Zheng , J. Shi , H. Tang , F. Huang , J. Agric. Food Chem. 2017, 66, 339. 29224351 10.1021/acs.jafc.7b04754

[159] Q. Luan , H. Zhang , J. Wang , Y. Li , M. Gan , Q. Deng , L. Cai , H. Tang , F. Huang , Food Hydrocolloids 2023, 142, 108804.

[160] H. Pan , F. Pei , G. Ma , N. Ma , L. Zhong , L. Zhao , Q. Hu , J. Food Eng. 2022, 334, 111170.

[161] M. Li , X. Hou , L. Lin , F. Jiang , D. Qiao , F. Xie , Int. J. Biol. Macromol. 2023, 243, 125217. 37285881 10.1016/j.ijbiomac.2023.125217

[162] J. Qiu , S. Xiang , M. Sun , M. Tan , J. Agric. Food Chem. 2023, 71, 18842. 37978937 10.1021/acs.jafc.3c05898

[163] W. C. Huang , W. J. Wang , W. Wang , Y. N. Hao , C. H. Xue , X. Z. Mao , Engineering 2023, 34, 187.

[164] L. B. Peres , R. S. dos Anjos , L. C. Tappertzhofen , P. E. Feuser , P. H. H. de Araújo , K. Landfester , C. Sayer , R. Muñoz‐Espí , Eur. Polym. J. 2018, 101, 341.

[165] X. S. Qin , Z. G. Luo , X. G. Li , Food Hydrocolloids 2021, 113, 106460.

[166] S. Teigelkamp , R. S. Bhardwaj , J. Roth , G. Meinardus‐Hager , M. Karas , C. Sorg , J. Biol. Chem. 1991, 266, 13462. 2071612

[167] J. Yang , M. Peng , S. Tan , S. Ge , L. Xie , T. Zhou , W. Liu , K. Zhang , Z. Zhang , J. Liu , J. Shi , ACS Cent. Sci. 2023, 9, 1327. 37521784 10.1021/acscentsci.3c00227 PMC10375893

[168] L. Qiu , R. Shen , L. Wei , S. Xu , W. Xia , Y. Hou , J. Cui , R. Qu , J. Luo , J. Cao , J. Yang , J. Sun , R. Ma , Q. Yu , J. Nanobiotechnol. 2023, 21, 344. 10.1186/s12951-023-02097-6 PMC10517557 37741962

[169] M. Han , W. Lei , J. Liang , H. Li , M. Hou , Z. Gao , Carbohydr. Polym. 2024, 324, 121472. 37985038 10.1016/j.carbpol.2023.121472

[170] a) F. Wu , J. Liu , Adv. Drug Delivery Rev. 2022, 188, 114443; 10.1016/j.addr.2022.114443 35817214

[171] a) X. Luo , H. Song , J. Yang , B. Han , Y. Feng , Y. Leng , Z. Chen , Colloids Surf., B 2020, 189, 110818; 10.1016/j.colsurfb.2020.110818 32018138

[172] X. Kuang , Y. Liu , H. Luo , Q. Li , F. Wu , C. Fan , J. Liu , J. Am. Chem. Soc. 2023, 145, 26932. 37988674 10.1021/jacs.3c10015

[173] A. G. Alkushi , A. Abdelfattah‐Hassan , H. Eldoumani , S. T. Elazab , S. A. M. Mohamed , A. S. Metwally , E. S. El‐Shetry , A. A. Saleh , N. A. ElSawy , D. Ibrahim , Sci. Rep. 2022, 12, 5116. 35332200 10.1038/s41598-022-08915-5 PMC8948303

[174] B. Deng , S. Lin , Y. Wang , M. Zhang , Y. Shen , P. Zhou , A. Shen , L. Wang , F. Ding , J. Liu , Adv. Mater. 2024, 37, 2412783. 10.1002/adma.202412783 39568244

[175] H. R. Jia , Y. X. Zhu , Z. Chen , F. G. Wu , ACS Appl. Mater. Interfaces 2017, 9, 15943. 28426936 10.1021/acsami.7b02562

[176] W. Geng , L. Wang , N. Jiang , J. Cao , Y. X. Xiao , H. Wei , A. Yetisen , K. X. Y. Yang , B. L. Su , Nanoscale 2018, 10, 3112. 29393952 10.1039/c7nr08556g

[177] X. Zhou , Q. Guo , M. Guo , B. Li , W. Peng , D. Wang , D. Ming , B. Zheng , J. Controlled Release 2021, 338, 742. 10.1016/j.jconrel.2021.09.009 34517041

[178] X. Xu , M. Zhang , X. Liu , M. Chai , L. Diao , L. Ma , S. Nie , M. Xu , Y. Wang , F. Mo , M. Liu , iScience 2023, 26, 107167. 37456845 10.1016/j.isci.2023.107167 PMC10338235

[179] K. Zhang , L. Zhu , Y. Zhong , L. Xu , C. Lang , J. Chen , F. Yan , J. Li , J. Qiu , Y. Chen , D. Sun , G. Wang , K. Qu , X. Qin , W. Wu , Adv. Sci. 2022, 10, 2205422. 10.1002/advs.202205422 PMC9896077 36507607

[180] H. Xu , Y. Wang , G. Liu , Z. Zhu , M. A. Shahbazi , R. L. Reis , S. C. Kundu , X. Shi , M. Zu , B. Xiao , Adv. Sci. 2024, 12, 2410011. 10.1002/advs.202410011 PMC11831460 39739630

[181] a) G. Fan , J. Cottet , M. R. Rodriguez‐Otero , P. Wasuwanich , A. L. Furst , ACS Appl. Bio Mater. 2022, 5, 4687; 10.1021/acsabm.2c00136 35535998

[182] A. Xie , H. Ji , Z. Liu , Y. Wan , X. Zhang , H. Xiong , S.‐P. Nie , H. Wan , ACS Nano 2023, 17, 14775. 37477584 10.1021/acsnano.3c02914

[183] X. Yang , J. Yang , Z. Ye , G. Zhang , W. Nie , H. Cheng , M. Peng , K. Zhang , J. Liu , Z. Zhang , J. Shi , ACS Nano 2022, 16, 4041. 35230097 10.1021/acsnano.1c09681

[184] Y. Lee , K. Sugihara , M. G. Gillilland , S. Jon , N. Kamada , J. J. Moon , Nat. Mater. 2019, 19, 118. 31427744 10.1038/s41563-019-0462-9 PMC6923573

[185] L. M. Zhu , T. T. Yu , W. Wang , T. Xu , W. J. Geng , N. Li , X. J. Zan , Adv. Mater. 2024, 36, 2308728. 10.1002/adma.202308728 38241751

[186] P. Zhu , L. Meng , Y. Shu , Y. Xu , W. Liu , Y. Bi , J. Xu , L. Meng , Y. Li , Carbohydr. Polym. 2024, 329, 121797. 38286561 10.1016/j.carbpol.2024.121797

[187] L. Yin , Z. Meng , Y. Zhang , K. Hu , W. Chen , K. Han , B.‐Y. Wu , R. You , C.‐H. Li , Y. Jin , Y.‐Q. Guan , J. Controlled Release 2018, 271, 31. 10.1016/j.jconrel.2017.12.013 29274436

[188] Y. Luo , C. De Souza , M. Ramachandran , S. Wang , H. Yi , Z. Ma , L. Zhang , K. Lin , J. Controlled Release 2022, 352, 371. 10.1016/j.jconrel.2022.10.030 36309096

[189] Q. Song , H. Zhao , C. Zheng , K. Wang , H. Gao , Q. Feng , H. Zhang , Z. Zhang , Y. Zhang , L. Wang , Adv. Mater. 2021, 31, 2104994.

[190] Q. Song , C. Zheng , J. Jia , H. Zhao , Q. Feng , H. Zhang , L. Wang , Z. Zhang , Y. Zhang , Adv. Mater. 2019, 31, 1903793. 10.1002/adma.201903793 31490587

[191] W. Hou , J. Li , Z. Cao , S. Lin , C. Pan , Y. Pang , J. Liu , Small 2021, 17, 2101810. 10.1002/smll.202101810 34365713

[192] M. Yang , H. Shen , S. Zhong , Z. Xu , X. Liu , W. Wu , C. Mao , M. Yang , Aggregate 2024, 5, 515.

[193] G. M. Luz , J. F. Mano , Compos. Sci. Technol. 2010, 70, 1777.

[194] Z. M. Geng , X. Y. Wang , F. Wu , Z. P. Cao , J. Y. Liu , Sci. Adv. 2023, 9, ade0997. 10.1126/sciadv.ade0997 PMC10022893 36930714

[195] W. Chen , Y. Wang , M. Qin , X. Zhang , Z. Zhang , X. Sun , Z. Gu , ACS Nano 2018, 12, 5995. 29786420 10.1021/acsnano.8b02235

[196] C. Pan , J. Li , W. Hou , S. Lin , L. Wang , Y. Pang , Y. Wang , J. Liu , Adv. Mater. 2021, 33, 2007379. 10.1002/adma.202007379 33629757

[197] J. Liu , Y. Wang , W. J. Heelan , Y. Chen , Z. Li , Q. Hu , Sci. Adv. 2022, 7, abf0677. 10.1126/sciadv.abp8798 PMC9651739 36367930

[198] J. Yang , G. Zhang , X. Yang , M. Peng , S. Ge , S. Tan , Z. Wen , Y. Wang , S. Wu , Y. Liang , J. An , K. Zhang , J. Liu , J. Shi , Z. Zhang , Chem. Eng. J. 2022, 446, 137204.

[199] J. Liu , W. Li , Y. Wang , Y. Ding , A. Lee , Q. Hu , Nano Today 2021, 41, 101291.

[200] W. F. Song , W. Q. Yao , Q. W. Chen , D. Zheng , Z. Y. Han , X. Z. Zhang , ACS Cent. Sci. 2022, 8, 1306. 36188344 10.1021/acscentsci.2c00533 PMC9523781

[201] S. Xie , L. Zhao , X. Song , M. Tang , C. Mo , X. Li , J. Controlled Release 2017, 268, 390. 10.1016/j.jconrel.2017.10.041 29101053

[202] N. Liao , B. Luo , J. Gao , X. Li , Z. Zhao , Y. Zhang , Y. Ni , F. Tian , Biotechnol. Lett. 2018, 41, 263. 30535881 10.1007/s10529-018-02634-6

[203] C. E. West , M. Dzidic , S. L. Prescott , M. C. Jenmalm , Allergol. Int. 2017, 66, 529. 28865967 10.1016/j.alit.2017.08.001

[204] A. Atia , A. Gomaa , I. Fliss , E. Beyssac , G. Garrait , M. Subirade , J. Microencapsulation 2016, 33, 89. 26805512 10.3109/02652048.2015.1134688

[205] J. Ma , C. Xu , H. Yu , Z. Feng , W. Yu , L. Gu , Z. Liu , L. Chen , Z. Jiang , J. Hou , Food Hydrocolloids 2021, 111, 106381.

[206] H. K. Makadia , S. J. Siegel , Polymers 2011, 3, 1377. 22577513 10.3390/polym3031377 PMC3347861

[207] F. Ajalloueian , P. R. Guerra , M. I. Bahl , A. M. Torp , E. T. Hwu , T. R. Licht , A. Boisen , Food Hydrocolloids 2022, 123, 107112.

[208] Z. Zhang , Y. Huang , R. Wang , R. Dong , T. Li , Q. Gu , P. Li , Int. J. Biol. Macromol. 2024, 260, 129624. 38262550 10.1016/j.ijbiomac.2024.129624

[209] H. Yu , W. Liu , D. Li , C. Liu , Z. Feng , B. Jiang , Polymers 2020, 12, 1565. 32679713 10.3390/polym12071565 PMC7407523

[210] K. Feng , M. Y. Zhai , Y. Zhang , R. J. Linhardt , M. H. Zong , L. Li , H. Wu , J. Agric. Food Chem. 2018, 66, 10890. 30260640 10.1021/acs.jafc.8b02644

[211] P. Wen , T. G. Hu , L. Li , M. H. Zong , H. Wu , Food Funct. 2018, 9, 5999. 30382268 10.1039/c8fo01216d

[212] Y. Zhang , C. Zhang , J. Wang , Y. Wen , H. Li , X. Liu , X. Liu , Crit. Rev. Food Sci. Nutr. 2023, 65, 1023. 38032153 10.1080/10408398.2023.2287185

[213] S. Kumar , J. Dutta , P. K. Dutta , J. Koh , Int. J. Biol. Macromol. 2020, 160, 470. 32464212 10.1016/j.ijbiomac.2020.05.192

[214] M. H. Land , K. Rouster‐Stevens , C. R. Woods , M. L. Cannon , J. Cnota , A. K. Shetty , Pediatrics 2005, 115, 178. 15629999 10.1542/peds.2004-2137

[215] T. Fang , S. Liu , Small 2023, 20, 2308146. 10.1002/smll.202308146 38054771

[216] J. An , E.‐M. Ha , Journal of Microbiology 2022, 60, 735.35781627

